# Multidimensional fidelity of the EPIC-Neck exercise programme for chronic non-specific neck pain: a UK feasibility trial process evaluation

**DOI:** 10.1136/bmjopen-2026-121944

**Published:** 2026-10-20

**Authors:** Jonathan Price, Alison Rushton, Natalie Ives, Marilyn Bradbury, Colin J Greaves, EPIC-Neck feasibility trial collaborators, Apurba Subedi

**Affiliations:** 1 Musculoskeletal Physiotherapy Services, Birmingham Community Healthcare NHS Foundation Trust, Aston, UK; 2 Research and Innovation, Birmingham Community Healthcare NHS Foundation Trust, Aston, UK; 3 School of Sport, Exercise and Rehabilitation Sciences, University of Birmingham, Birmingham, UK; 4 School of Physical Therapy, Western University Faculty of Health Sciences, London, Ontario, Canada; 5 Institute of Applied Health Research, College of Medical and Dental Sciences, University of Birmingham, Birmingham, UK

**Keywords:** Musculoskeletal disorders, Exercise, Behavior, REHABILITATION MEDICINE, Neck Pain, Intervention Fidelity, Process Evaluation

## Abstract

**Objectives:**

To evaluate multidimensional fidelity of the EPIC-Neck programme (Exercise Prescription Improved through Co-design).

**Design:**

Mixed-methods fidelity evaluation embedded within a randomised controlled feasibility study.

**Setting:**

UK National Health Service (NHS) musculoskeletal outpatient services.

**Participants:**

Physiotherapists delivering and adults with chronic non-specific neck pain receiving the EPIC-Neck programme.

**Intervention:**

EPIC-Neck is an individualised, evidence- and theory-informed exercise programme combining optimised exercise prescription and behaviour change techniques to support four performance objectives (POs): perform prescribed exercise (PO1), progress/regress exercise (PO2), use exercise to manage flare-ups (PO3) and engage in general exercise (PO4).

**Fidelity evaluation:**

Fidelity was evaluated in four domains: training, delivery, receipt and enactment. Quantitative data were questionnaires on physiotherapist confidence pretraining/post-training (1–5 Likert scale) and training satisfaction (training), a 17-item delivery fidelity checklist applied to audio-recorded appointments (0–5; 3=acceptable) (delivery) and patient-reported confidence for undertaking POs 1–3 (receipt). Quantitative data were analysed descriptively.

Qualitative data were physiotherapists’ interviews on training and delivery and patient interviews on PO understanding, confidence and completion (receipt, enactment). Interviews underwent inductive-deductive thematic analysis.

Quantitative and qualitative data were integrated through narrative weaving for training/receipt and connected sequentially for delivery.

**Results:**

24 participants were randomised to EPIC-Neck; 22 contributed quantitative delivery data, 18 contributed quantitative receipt data, and 12 were interviewed. Seven physiotherapists delivered EPIC-Neck, and five were interviewed. Physiotherapist confidence improved after training (median 3/5 to 4/5), but qualitative data indicated they remained underprepared. Mean delivery fidelity was below the acceptable threshold (2.4/5, SD 1.3), with the poorest delivery for social support, problem-solving and PO2–PO4 (<2/5). Participants generally understood and enacted prescribed neck exercises, but receipt and enactment were weaker for complex/resisted exercises and longer-term self-management behaviours (PO2–PO4).

**Conclusion:**

Although training improved confidence, it did not fully prepare physiotherapists for the delivery of more complex components. Overall delivery fidelity was below the acceptable threshold, indicating that EPIC-Neck was not consistently delivered as intended. Receipt and enactment were stronger for performing neck exercises than for longer-term self-care behaviours. Intervention and training refinement are required before a definitive trial.

**Trial registration number:**

ISRCTN81746901.

**STRENGTHS AND LIMITATIONS OF THIS STUDY**

- This study used a theory-informed, mixed-methods fidelity evaluation, guided by the National Institutes of Health Behaviour Change Consortium framework, enabling assessment across training, delivery, receipt and enactment rather than relying on single-domain or proxy measures.
- Direct observation of intervention delivery using audio-recorded consultations and a structured fidelity checklist provided a robust and objective assessment of delivery quality in routine NHS practice, strengthening internal validity.
- Integration of quantitative and qualitative data using domain-specific mixed-methods designs (convergent and explanatory sequential) allowed exploration of how training and delivery influenced receipt and enactment, enhancing interpretive depth.
- Enactment fidelity was assessed qualitatively at discharge only, which may not fully capture longer-term self-management behaviours, particularly general exercise engagement beyond the treatment period.
- Some fidelity measures (eg, confidence questionnaires) were purpose-designed rather than formally validated, reflecting programme theory but potentially limiting comparability with other studies.

## Background

 Neck pain is a growing global health concern. Global Burden of Disease modelling indicates that the global age-standardised years lived with disability rate attributable to neck pain has increased in recent years and is projected to rise by 35% (from 267.348 per 100 000 in 2019 to 360.034 per 100 000) by 2044.^1^ Chronic non-specific neck pain contributes substantially to this burden because symptoms are often recurrent or persistent and are associated with disability, psychological distress and healthcare use.^2–6^

Guidelines recommend physiotherapist-prescribed neck exercises, yet evidence shows only modest effects that decline over time.^5 7–18^ These suboptimal outcomes have been linked to poor adherence, limited individualisation, implementation barriers and insufficient attention to biopsychosocial factors.^10 19–30^ Neck exercise must be optimised to address biopsychosocial factors, improve adherence, allow individualisation and be implementable in practice.

To address these challenges, complex intervention development frameworks offer a structured, evidence-based process for intervention optimisation.^31–33^ Using these principles, the EPIC-Neck programme (Exercise Prescription Improved through Co-design), a codeveloped, theory- and evidence-informed exercise programme, was developed using Intervention Mapping.^33 34^ The programme was designed to maximise engagement with and the effectiveness of neck exercise for people with chronic non-specific neck pain. EPIC-Neck combines individualised, physiotherapist-prescribed biopsychosocial exercise with behaviour change strategies and patient-facing resources to support exercise-based self-management.

Given the multiple interacting components within EPIC-Neck, understanding whether the programme can be delivered, received and enacted as intended is an essential step before definitive effectiveness testing.^32 35^ The UK Medical Research Council recommends that feasibility studies evaluate fidelity to identify implementation uncertainties that may influence intervention effectiveness and to inform refinement prior to a definitive trial.^32 35^ Fidelity refers to the extent to which an intervention is implemented as intended.^36^ Without adequate fidelity evaluation, it is difficult to determine whether observed outcomes reflect the intervention itself or variation in its implementation.^37^

Despite this, fidelity remains underevaluated and inconsistently reported within musculoskeletal research.^24 38–40^ This evaluation was guided by the National Institutes of Health Behaviour Change Consortium (NIH-BCC) framework because it is a well-established and highly cited framework for behavioural interventions, has been used in multidimensional fidelity evaluations and provides coverage of domains relevant to complex intervention evaluation.^41–43^ The NIH-BCC framework comprises five domains: design, training, delivery, receipt and enactment, aligning with the EPIC-Neck programme theory by assessing whether physiotherapists were trained to deliver the intervention, whether EPIC-Neck was delivered as intended and whether participants understood and enacted the intended exercise behaviours.^41^ Therefore, this process evaluation aimed to establish the multidimensional fidelity of the EPIC-Neck programme and identify refinements before progressing to a full-scale effectiveness trial.

### Aims and objectives

Aim: to evaluate the multidimensional fidelity of the EPIC-Neck programme.

Objectives:

- Training fidelity: assess the extent to which training developed physiotherapists’ capability (skills, knowledge and confidence) to deliver EPIC-Neck as intended and identify areas for refinement.
- Delivery fidelity: determine the extent to which EPIC-Neck was delivered as intended and identify barriers/facilitators to delivery.
- Receipt fidelity: explore whether participants were confident in and understood EPIC-Neck’s performance objectives (POs).
- Enactment fidelity: estimate the extent to which participants performed EPIC-Neck’s POs and identify any barriers/facilitators to enactment.

## Methods

### Study design

This mixed-methods process evaluation was embedded within the EPIC-Neck randomised controlled feasibility study, which aimed to determine the feasibility of a definitive trial evaluating the clinical and cost-effectiveness of EPIC-Neck compared with usual exercise care for adults with chronic non-specific neck pain. In the parent feasibility study, patients attending UK NHS musculoskeletal outpatient physiotherapy services were randomised to EPIC-Neck or usual exercise care (physiotherapists’ routine practice).^44 45^ Protocol for the parent feasibility study is in online supplemental file 6. The present study reports the nested multidimensional fidelity evaluation of the EPIC-Neck intervention. Usual exercise care appointments were included only to examine whether EPIC-Neck components appeared in usual care.

### EPIC-Neck programme

Full details of the EPIC-Neck programme, development processes and theoretical underpinnings are published elsewhere.^34^ In brief, EPIC-Neck integrates individualised biopsychosocial exercise prescription with structured behaviour change support.^34^ Physiotherapists prescribed individualised exercise using a biopsychosocial exercise prescription framework, with exercise selection and dosage guided by intended outcomes, key mediators and tailoring strategies (online supplemental file 1, Figure S1). Behaviour change was supported using a facilitation guide that included techniques such as goal setting, self-monitoring, engaging social support and action planning, delivered through supported problem-solving and patient-centred communication (online supplemental file 2).^46–48^ Behaviour change support focused on helping patients achieve four sequential POs:

- PO1—perform physiotherapist-prescribed neck exercises.
- PO2—independently adapt and progress (or regress) neck exercises.
- PO3—use neck exercises to manage flare-ups.
- PO4—engage in long-term general exercise, with or without secondary prevention neck exercises.

PO4 comprises two exercise behaviours: engagement in general exercise, which was intended for all participants, and secondary-prevention neck exercise, which was conceptualised as an optional shared-decision-making behaviour because evidence for recurrence prevention remains uncertain.^34^

#### Delivery

EPIC-Neck was delivered face-to-face by trained physiotherapists, with follow-up appointments used to review progress, modify exercises and support performance objective achievement. Participants received paper and digital materials to support exercise completion and behaviour change between appointments. Discharge was determined collaboratively, based on progress towards goals, performance objective achievement and the participant’s ability to problem-solve independently.

#### Physiotherapist training

Physiotherapists delivering EPIC-Neck completed 1-day face-to-face training using lectures, practical activities and case-study role-play.^49 50^ Training was delivered by JP, the lead developer of EPIC-Neck, and was designed to align with NHS service capacity and physiotherapist availability.^34^ Ongoing physiotherapist support was provided through training materials, crib sheets, self-reflection checklists, optional site-specific top-up sessions, clinical champions, email updates and peer support.^49 51^ Training was designed to address anticipated physiotherapist barriers to intervention delivery, informed by physiotherapist workshops, the practical experience of the study team and relevant literature.^52–54^ Training techniques mapped to delivery barriers are reported in online supplemental file 3, Table S1.

### Patient participants

Adults (≥18 years) with chronic (≥3 months) non-specific neck pain were recruited from NHS community-based and secondary care musculoskeletal services in Berkshire, Birmingham and Nottingham. Non-specific neck pain was defined as pain or discomfort in the neck and/or shoulder girdle, with or without referred arm pain, for which no specific cause could be identified.^55 56^ Exclusion criteria included neck pain due to trauma (eg, whiplash), a specific diagnosis (eg, rheumatoid arthritis, osteoarthritis) and neurological involvement (eg, radiculopathy, myelopathy). Full eligibility criteria are reported elsewhere.^44^ All participants randomised to the EPIC-Neck intervention who attended appointments and/or returned questionnaires contributed to quantitative fidelity evaluation (delivery/receipt). A purposive subsample (capturing diversity in age, sex, ethnicity, neck pain-related disability and deprivation) was invited to participate in qualitative interviews to explore receipt and enactment. Written informed consent for all fidelity procedures was obtained as part of the feasibility study.

### Physiotherapists

Physiotherapists were recruited from participating NHS musculoskeletal services and consented to all fidelity-related procedures. Only physiotherapists who completed EPIC-Neck training and delivered the programme were eligible for interviews.

### Multidimensional fidelity evaluation

Fidelity evaluation of the EPIC-Neck programme was guided by the NIH-BCC framework covering training, delivery, receipt and enactment, using a mixture of qualitative and quantitative methods (table 1).^36 41 57 58^ Design fidelity was addressed during intervention development, where the theoretical mechanisms, active components and intended implementation of EPIC-Neck were specified using Intervention Mapping methods and reported previously.^34^ This process evaluation therefore focused on empirically assessing training, delivery, receipt and enactment fidelity.

**Table 1:** Fidelity domain data collection, analysis and integration

Fidelity domain	Operational definition	Quantitative collection and analysis	Qualitative collection and analysis	Integration
Training	Extent to which training developed physiotherapist capability (knowledge, skills and confidence) to deliver EPIC-Neck	Physiotherapist pretraining/post-training confidence ratings (32 components, Likert 1–5) Physiotherapist post-training satisfaction survey Analysis: median, IQR, proportions	Semistructured one-to-one interviews with physiotherapists at the end of the study Analysis: deductive+inductive thematic analysis	Convergent; analysed separately and merged via narrative weaving
Delivery	The degree to which physiotherapists administered EPIC-Neck components and the quality of their delivery.	Audio-recorded appointments scored against a 17-item checklist (0–5 Dreyfus scale; 0=absent, 3=acceptable, 5=perfect delivery) Analysis: overall study, component, participant and physiotherapist level means±SD	Semistructured one-to-one interviews with physiotherapists at the end of the study Analysis: deductive+inductive thematic analysis	Explanatory sequential; interviews followed checklist scoring to explain quantitative findings through narrative weaving.
Receipt	Participant understanding of and confidence in the four POs	Participant confidence completing PO1-3 questionnaire (one item per PO, Likert 1–5) at 6 months postrandomisation Analysis: median, IQR, proportions	Semistructured one-to-one interviews with participants at discharge Analysis: deductive+inductive thematic analysis	Convergent; analysed separately and merged via narrative weaving and PO-specific joint display
Enactment	Extent to which participants performed the four POs and used patient-facing resources	Not applicable	Semistructured one-to-one interviews with participants at discharge Analysis: deductive+inductive thematic analysis	Not applicable

.EPIC, Exercise Prescription Improved through Co-design; IQR, Interquartile Range; PO, Performance Objective; SD, Standard Deviation.

### Data collection

#### Quantitative

##### Training

Physiotherapists completed a questionnaire immediately pretraining and post-training, rating confidence in delivering key components on a 5-point Likert scale (1=not confident, 3=somewhat confident, 5=completely confident) and a post-training survey evaluating training satisfaction, strengths/limitations and suggested refinements. These questionnaires were study-specific measures developed to align with the EPIC-Neck logic model and fidelity objectives and were not formally validated.

##### Delivery

All EPIC-Neck appointments were audio-recorded and scored against a predefined fidelity checklist covering 17 intended programme components (online supplemental file 3, table S3).^53 59^ The checklist was developed by JP based on the EPIC-Neck logic model, intervention materials and fidelity literature and was reviewed by CG, a health psychologist with expertise in behaviour change interventions. Delivery quality of checklist items was scored using the Dreyfus clinical competence scale (0–5), where scores represent increasing levels of competence, with 3 indicating acceptable delivery and 5 indicating expert/perfect delivery.^60^ Consistent with previous fidelity research using this approach, a score of ≥3/5 was used as the threshold for acceptable delivery fidelity.^52 53 61^ Two researchers independently coded two appointments and discussed discrepancies. This process was repeated until at least 75% of scores were within one point of each other, at which point a single researcher coded the remaining recordings.^52 62^

##### Receipt

Confidence completing POs 1–3 was assessed at 6 months postrandomisation using a 3-item questionnaire, comprising one item for each performance objective, with an anchored 1–5 Likert scale (1=very unconfident, 3=no opinion, 5=very confident).^42 63^

##### Enactment

Enactment fidelity was assessed qualitatively through participant interviews. Quantitative enactment measurement was considered during study development but was not included because meaningful enactment varied by performance objective and context. For example, PO2 involved nuanced decisions about adapting, progressing or regressing exercises; PO3 was only applicable when participants experienced flare-ups; and PO4 involved decision-making about general exercise and optional secondary-prevention neck exercise. Based on the patient and public involvement and engagement (PPIE) input, proposed quantitative performance-objective measures were removed to reduce participant burden and because they risked being too crude to capture the complexity of enactment in this context.

### Qualitative

Semistructured interviews were conducted with participants postdischarge and with physiotherapists at study end. Physiotherapist interviews explored training experiences, preparedness, ideas for refining training (training fidelity) and explanations for quantitative delivery-fidelity scores, focusing on lower-scoring components (delivery fidelity).^41 64^ Patient interviews explored understanding, confidence (receipt) and completion of POs (enactment), in addition to barriers/facilitators to these outcomes.^42^ Interview topic guides were informed by the EPIC-Neck logic model, quantitative training and delivery analyses and PPIE feedback (online supplemental file 4).

Interviews were conducted by JP by telephone, Microsoft Teams or face-to-face; lasted up to 90 min and were audio-recorded and transcribed verbatim. JP (chief investigator and lead intervention developer) had existing professional relationships with some physiotherapists (none with participants) and did not deliver the intervention. Rapport, reflective listening and confidentiality were emphasised. Notes were taken but no reflexive journal was kept. A structured topic guide supported consistency and minimised bias.

### Data analysis

#### Quantitative

Descriptive statistics (mean, SD, median, IQR and proportions) were used. As an exploratory analysis, overall pretraining/post-training confidence was compared using a Wilcoxon signed-rank test, based on each physiotherapist’s mean confidence score across all training components. Missing data were not imputed, and missed appointments were excluded from delivery-fidelity means. Analyses were performed in Microsoft Excel (V.2512, Microsoft, Redmond, Washington) and IBM SPSS Statistics (V.30, IBM, Armonk, New York).

Delivery fidelity was assessed at participant, physiotherapist and study levels. For each participant, one-off items used the maximum score across appointments; all other items used the mean. These item scores were averaged to generate participant-level and then study-level fidelity scores (mean ± SD), with ≥3 indicating acceptable delivery. Item-level means across participants described fidelity to each intervention component. Physiotherapist-level scores were derived when participants were treated exclusively by one physiotherapist. Usual-care appointments were also audio-recorded and scored using the same checklist to compare practice and support validation of training and design fidelity.

#### Qualitative

Due to resource limitations, JP coded and analysed all interview transcripts. No formal independent double-coding or inter-coder reliability checks were conducted. Physiotherapist interviews underwent inductive and deductive thematic analysis, informed by the delivery-fidelity checklist and EPIC-Neck logic model, to explore preparedness, training satisfaction, optimisation and delivery-related barriers and facilitators.^65^ Patient interviews were analysed using thematic analysis inductively and deductively using the logic model to identify themes related to performance-objective understanding, confidence, completion and associated barriers and facilitators.^65^ As secondary-prevention exercise was an optional shared-decision-making component and could not reasonably be enacted at discharge, qualitative enactment analysis for PO4 focused on general exercise only. Receipt analysis for PO4 examined participants’ understanding of general exercise and their understanding of the potential benefits and risks of secondary prevention exercise.

Themes and coding decisions were reviewed with the study team to enhance credibility, with disagreements resolved through discussion. Member-checking was not undertaken, as interviews focused primarily on fidelity assessment rather than the coconstruction of meaning. NVivo 12 Plus (QSR International) supported data management, coding and audit trail maintenance.

#### Integration

Mixed-methods integration varied by fidelity domain and followed Creswell and Plano Clark recommendations (table 1).^57 58^ Training and receipt fidelity were examined using a convergent design, with quantitative and qualitative data analysed separately and then merged through narrative weaving to identify areas of agreement, complementarity or divergence. For receipt fidelity, a PO-specific joint display integrated quantitative and qualitative receipt data with qualitative enactment findings. Delivery fidelity used an explanatory sequential design, in which qualitative interviews followed checklist scoring and were connected to the quantitative results to explain component-level delivery scores, with integration represented through narrative weaving. Enactment fidelity was assessed using qualitative data alone.

### Patient and public involvement and engagement (PPIE)

The protocol, intervention development, study materials and dissemination were informed by a PPIE group (n=17) (online supplemental file 5—Guidance for Reporting Involvement of Patients and the Public 2 - Short Form (GRIPP2-SF)).^66^ Two PPIE members were part of the Study Management Group.

## Results

### Patient participant characteristics

24 participants were randomised to EPIC-Neck and 12 to Usual Exercise Care (figure1). All EPIC-Neck intervention participants who attended at least one appointment were included in the delivery fidelity analysis. 18 out of 24 EPIC-Neck intervention participants returned quantitative receipt data; the remaining six did not return the 6-month questionnaire despite follow-up. 17 participants were invited to interviews, and 12 took part. Two were not contactable, and three declined due to not wanting to use a professional interpreter (n=2) or a lack of time (n=1). Participant characteristics are in table 2.

**Figure 1:**
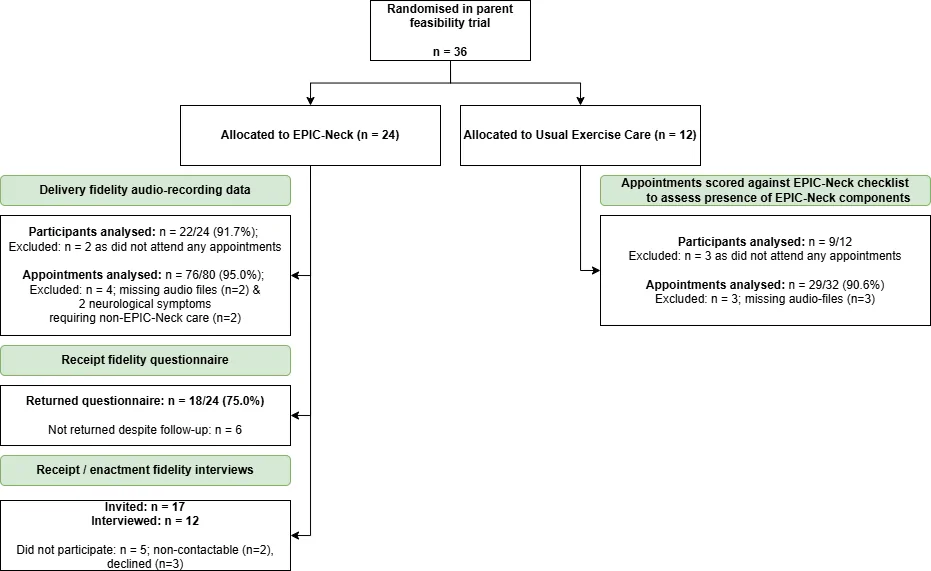
Participant contribution to fidelity evaluation datasets. EPIC, Exercise Prescription Improved through Co-design.

**Table 2:** Characteristics of EPIC-Neck participants randomised, returning receipt questionnaires and completing receipt/enactment

Demographic characteristics	Randomised to EPIC-Neck n=24		Returned receipt questionnaire at 6 months n=18		Completed receipt/enactment interviews n=12	
	n	%	n	%	n	%
**Sex**						
Female	11	45.8	8	44.4	4	33.3
Male	13	54.2	10	55.6	8	66.7
**Age**						
18–30	3	12.5	2	11.1	1	8.3
31–40	7	29.2	4	22.2	3	25.0
41–50	3	12.5	2	11.1	2	16.7
51–60	7	29.2	6	33.3	3	25.0
61+	4	16.7	4	22.2	3	25.0
**Ethnicity**						
**Asian or Asian British**	**9**	**37.5**	**7**	**38.9**	**3**	**25.0**
Indian	2	8.3	2	11.1	0	0.0
Pakistani	7	29.2	5	27.8	3	25.0
**Black or Black British**	**3**	**12.5**	**2**	**11.1**	**2**	**16.7**
African	1	4.2	0	0.0	0	0.0
Caribbean	1	4.2	1	5.6	1	8.3
Other	1	4.2	1	5.6	1	8.3
**Mixed**	**1**	**4.2**	**1**	**5.6**	**1**	**8.3**
White & Black Caribbean	1	4.2	1	5.6	1	8.3
**White**	**10**	**41.7**	**7**	**38.9**	**5**	**41.7**
British	9	37.5	7	38.9	5	41.7
Irish	1	4.2	0	0.0	0	0.0
**Other**	**1**	**4.2**	**1**	**5.6**	**1**	**8.3**
Arab	1	4.2	1	5.6	1	8.3
**IMD decile**						
Most deprived (deciles 1–3)	19	79.2	14	77.8	10	83.3
Middle (deciles 4–7)	4	16.7	4	22.2	2	16.7
Least deprived (deciles 8–10)	1	4.2	0	0.0	0	0.0
**Neck pain-related disability (NDI)**						
No disability	3	12.5	3	16.7	2	16.7
Mild disability	8	33.3	6	33.3	5	41.7
Moderate disability	12	50.0	8	44.4	5	41.7
Severe disability	1	4.2	1	5.6	0	0.0
Complete disability	0	0.0	0	0.0	0	0.0

Questionnaire and interview columns are subsets of the EPIC-Neck randomised group. Percentages are presented alongside n values for transparency and should be interpreted cautiously where subgroup denominators are small. Ethnicity categories were retained to support reporting of sample inclusivity. Bold entries indicate demographic headings and parent categories; indented entries indicate subcategories.

IMD, Index of Multiple Deprivation; NDI, Neck Disability Index.

### Physiotherapist characteristics

Nine of 10 consenting physiotherapists completed EPIC-Neck training and provided pretraining/post-training confidence and training survey data. Of these, seven delivered the EPIC-Neck programme; one trained physiotherapist left the service before delivering the intervention, and one did not deliver because no eligible participants were booked into their diary. Five of these participated in semistructured interviews, contributing qualitative training and delivery fidelity data (two declined due to sick/bereavement leave). Physiotherapist characteristics are in table 3.

**Table 3:** Physiotherapist characteristics

	Trained: confidence and survey data n=9		Delivered EPIC-Neck n=7		Interviews n=5	
	n	%	n	%	n	%
**Sex**						
Female	5	56	3	43	3	60
Male	4	44	4	57	2	40
**Age**						
20–29	1	11	1	14	1	20
30–39	2	22	2	29	1	20
40–49	3	33	2	29	1	20
50+	3	33	2	29	2	40
**Highest qualification**						
Bachelor’s degree	6	67	6	86	5	100
Master’s degree	3	33	1	14	0	0
**Years of experience treating chronic non-specific neck pain**						
0–2	3	33	2	29	2	40
3–5	1	11	1	14	0	0
6–10	2	22	2	29	2	40
11+	3	33	2	29	1	20
**Postgraduate training areas**						
Neck pain assessment and treatment	6	67	5	71	3	60
Exercise prescription	4	44	4	57	3	60
Behaviour change	3	33	2	29	2	40
Psychologically informed practice	1	11	1	14	1	20
Patient-centred communication techniques	4	44	3	43	3	60

### Training

#### Quantitative

Quantitative training data are in online supplemental file 3, table S2 and date S1. Overall confidence increased from ‘somewhat confident’ (median=3, IQR=2) to ‘fairly confident’ (median=4, IQR=1). An exploratory Wilcoxon signed-rank test of physiotherapists’ overall mean confidence scores across all components indicated a statistically significant increase from baseline to post-training (Z=−2.55, p=0.011). Median confidence increased for 22/32 EPIC-Neck components and remained unchanged with reduced variability (lower IQR) for 6/32 components. Four components showed no change in median or IQR scores (person-centred communication, prescribing for neuromuscular dysfunction, progressing exercise and regressing exercise). The lowest post-training confidence remained for prescribing exercise for pain self-efficacy, mental well-being, social functioning/relationships, engaging social support and patient-centred communication (all median = 3). No component reached ‘completely confident’ (median=5). Post-training survey scores indicated high satisfaction (89%–100% rating the training as good/excellent), with comments praising role-play and case studies but noting the high content load in a single day and a need for more practice time.

#### Qualitative

Six themes were constructed from interview data (online supplemental file 3, data S2). Training improved physiotherapists’ capability (skills, knowledge and confidence), but physiotherapists did not feel fully capable of applying the programme in routine practice (Theme 1). Role-play, case discussions and practical components were described as helpful for capability development, but physiotherapists wanted additional opportunities to practise (Theme 2). Support from experts and peers facilitated learning, although the absence of formal, individualised delivery feedback was viewed as a limitation (Theme 3). The volume of content within a single-day format, limited practice time and the long gap between training and intervention delivery were identified as barriers to capability development, with physiotherapists suggesting that training should be spread across sessions and include more opportunities for practice and feedback (Themes 4–5). Although self-directed content and refresher learning were considered beneficial, they were rarely used (Theme 6).

#### Integration

Quantitative and qualitative findings agreed that training improved physiotherapists’ capability but did not fully prepare them for programme delivery. Both datasets indicated that practical components supported learning, yet additional practice was needed to develop implementation-ready skills. There was partial agreement that the single-day, high-volume format limited consolidation and preparedness.

### Delivery

#### Quantitative

EPIC-Neck participants attended a mean of 3.3 appointments (SD 1.7; range 0–7) over 16.9 weeks (SD 8.6; range 0–36). Delivery fidelity was scored for 76/80 appointments; four were excluded due to missing audio (n=2) or because participants reported neurological symptoms requiring non-EPIC-Neck care (n=2). Initial piloting of the delivery checklist (two researchers scoring two appointments each) yielded 67% agreement. Following discussion, agreement increased to 84% and then 82% (across a further four and then three appointments, respectively), before the remaining recordings were scored by a single researcher (JP).

Overall delivery fidelity was below the predefined acceptability threshold (≥3) (mean 2.4, SD 1.3). Participant mean scores ranged from 1.3 to 3.2, with three participants (14%) receiving the intervention at an acceptable standard (≥3). Component scores ranged from 0.9 to 3.5, with six components (35%) being delivered to an acceptable standard (≥3) and five components (29%) scoring <2 (table 4). Techniques delivered well or poorly for all components are summarised in online supplemental file 3, table S4. Mean physiotherapist delivery fidelity was 2.3 (SD 1.2; range 1.6–2.8).

**Table 4:** Appointment audio-recording delivery fidelity scores by EPIC-Neck component

Component	EPIC-Neck			Usual exercise care		
	N	Mean (SD)	95% CI	N	Mean (SD)	95% CI
01—agree outcome goals	22	2.0 (1.3)	1.5 to 2.6	9	0.9 (0.9)	0.2 to 1.6
02—actively involve the patient	22	3.1 (0.7)	2.8 to 3.4	9	1.2 (0.4)	0.9 to 1.5
03—improve motivation to perform exercise (PO1)—enhance perceived importance and safety	22	2.1 (1.0)	1.6 to 2.5	9	1.4 (0.6)	1.0 to 1.9
04—improve motivation to perform exercise (PO1)—improve self-efficacy	22	3.5 (0.8)	3.1 to 3.9	9	3.0 (1.1)	2.1 to 3.9
05—improve motivation to perform exercise (PO1)—engage social support	22	1.8 (1.1)	1.4 to 2.3	9	0.4 (0.5)	0.0 to 0.8
06—set a plan to perform exercise (PO1)	22	2.3 (1.0)	1.9 to 2.7	9	1.0 (0.0)	1.0 to 1.0
07—encourage self-monitoring (PO1)	22	3.3 (0.9)	2.9 to 3.8	9	0.0 (0.0)	0.0 to 0.0
08—review progress	19	2.7 (0.5)	2.5 to 3.0	7	1.6 (0.3)	1.3 to 1.9
09—support problem solving	18	1.3 (0.7)	1.0 to 1.7	7	0.8 (0.5)	0.3 to 1.2
10—introduce adapting and progressing (or regression) exercise (PO2)	22	1.6 (1.1)	1.2 to 2.1	9	0.2 (0.4)	−0.1 to 0.6
11—introduce flare-up exercise (PO3)	22	1.5 (1.0)	1.1 to 2.0	9	0.1 (0.3)	−0.1 to 0.4
12—introduce general exercise±secondary prevention exercise (PO4)	22	0.9 (0.9)	0.5 to 1.3	9	0.1 (0.3)	−0.1 to 0.4
13—evaluate self-management and discharge suitability	21	2.0 (1.2)	1.4 to 2.5	9	0.8 (0.9)	0.1 to 1.5
14—identify short-term outcomes and exercise objectives (PO1)	22	3.1 (0.8)	2.8 to 3.5	9	1.0 (0.0)	1.0 to 1.0
15—home exercise programme—normalising movement (PO1)	22	3.5 (0.7)	3.2 to 3.8	9	2.0 (1.0)	1.2 to 2.8
16—home exercise programme—improving tolerance (PO1)	22	2.7 (1.6)	2.0 to 3.5	9	2.2 (0.7)	1.7 to 2.7
17—exercise review (PO1)	19	3.0 (1.2)	2.4 to 3.5	7	2.2 (1.1)	1.2 to 3.1
	–	2.4 (1.3)	2.3 to 2.5	–	1.1 (1.0)	0.9 to 1.3

Colour coding applies to the EPIC-Neck column only. Green (3.0–5.0)—acceptable delivery of EPIC-Neck component; amber (2.0–2.9)—minor delivery concerns; red (0.0–1.9)—major delivery concerns. Usual exercise care scores represent concordance with EPIC-Neck components and should not be interpreted as indicating poor quality of usual exercise care.

Usual exercise care appointments contained few EPIC-Neck components, suggesting limited contamination between study arms. When scored against the EPIC-Neck checklist, usual exercise care had a mean concordance score of 1.1 (SD 1.0; range 0.6–1.5). These descriptive differences suggest that usual exercise care rarely incorporated EPIC-Neck components. Only one component (improving self-efficacy) was delivered in a manner consistent with the EPIC-Neck protocol (table 4). Full participant-by-component fidelity scores for both study arms are provided in online supplemental file 3, table S5 and S6.

#### Qualitative

Six themes were generated from physiotherapist interview data to describe barriers and facilitators to delivery fidelity (online supplemental file 3, data S3). Delivery was shaped by how easily components could be integrated with routine practice (Theme 1); physiotherapist capability (Theme 2); the perceived relevance and comprehensiveness of intervention components (Theme 3); patient-level factors (such as beliefs, expectations and communication barriers) (Theme 4); physiotherapist- and patient-facing resources (Theme 5) and organisational pressures (Theme 6).

#### Integration

Qualitative findings explained the quantitative pattern of delivery scores. Components delivered acceptably (≥3) were those physiotherapists described as similar to, or easy to integrate into, routine practice, such as communication techniques, history-taking activities and self-efficacy strategies. Training was perceived to have effectively developed capability in these areas, and several components were supported by physiotherapist- and patient-facing resources that prompted or structured delivery. In contrast, components that were delivered poorly (<2) (social support, problem-solving, PO2–4) required greater deviation from usual practice and increased cognitive effort, contributing to superficial or inconsistent delivery. Physiotherapists described capability gaps in these components and sometimes overestimated their performance in comparison to observed delivery. Some components were intentionally delivered superficially or abandoned when perceived as making patients uncomfortable or resistant, while others were deprioritised when symptoms improved or when service pressures encouraged early discharge. For social support-related content, low delivery sometimes reflected passive reliance on written materials rather than active, in-session discussion. Detailed component explanations are provided in online supplemental file 3, table S4.

### Receipt

Receipt findings are summarised in table 5.

**Table 5:** Integrated receipt and enactment fidelity findings from confidence ratings and interviews

Performance objective	Receipt (quantitative)	Receipt (qualitative)	Enactment	Integration interpretation
PO1: performing exercise	High confidence (94%)	Most understood; lower for complex tasks	Most enacted, but selectively	Agreement: good receipt translated into enactment, but complexity limited full implementation
PO2: adapting and progressing (or regressing) exercise	High confidence (89%)	Partial understanding of progression	Some enacted; inconsistent adaptation	Partial agreement: confidence overestimated understanding, limiting enactment
PO3: managing flare-ups with exercise	High confidence (89%)	Inconsistent/incomplete understanding	Few enacted appropriate strategies	Divergence: poor understanding prevented enactment despite high confidence
PO4: engage in general exercise with or without secondary prevention exercise*	No data	Limited understanding of general exercise or secondary prevention exercise benefits and risks	Minimal enactment of general exercise	Low receipt associated with low enactment

Quantitative receipt data are the proportion rating themselves as confident or very confident.

* PO4 comprised two related behaviours: engagement in general exercise, which was intended for all participants, and secondary-prevention neck exercise, which was optional and based on shared decision-making. As interviews occurred at discharge, enactment analysis for PO4 focused on general exercise only; only understanding of the benefits and risks of secondary-prevention exercise was assessed within qualitative receipt.

#### Quantitative

Patient participants were confident performing PO1–PO3 at 6 months. Median confidence scores were 4.00 for PO1 (IQR 0.75), 4.00 for PO2 (IQR 1.00) and 4.00 for PO3 (IQR 0.00), with 94% (17/18), 89% (16/18) and 89% (16/18) of participants, respectively, rating themselves as confident or very confident (4–5/5).

#### Qualitative

Interview findings generated PO-specific themes describing participant understanding, confidence and barriers/facilitators to receipt (online supplemental file 3, data S4).

##### PO1: performing prescribed exercise

Most participants understood and felt confident performing exercises, but several reported lower confidence and understanding for complex or resisted exercises (Theme 1). Safety was broadly understood, but some reported persistent fear of causing harm, which undermined confidence (Theme 2). Participants described several sources that supported their understanding of how to perform the exercises, but most identified physiotherapist demonstrations as the most helpful (Theme 3).

##### PO2: adapting and progressing/regressing exercises

Most participants understood how to adapt, progress or regress exercises, often citing repetition changes, but gaps remained in knowledge of alternative strategies (Theme 1). Symptom responses and perceived exercise difficulty guided decision-making (Theme 2). Participants reported that their understanding was shaped by both prior exercise experience and physiotherapist guidance (Theme 3).

##### PO3: self-managing flare-ups with exercise

Understanding of exercise-based flare-up management varied considerably. Some participants’ understanding was consistent with the intervention protocol, while other participants’ understanding relied on non-exercise strategies (eg, seeking healthcare support), and some reported no understanding (Theme 1). Participants reported that physiotherapists were key in developing understanding (Theme 2).

##### PO4: general exercise and understanding of the benefits and risks of secondary-prevention exercise

Most participants expressed a desire to engage in general exercise but didn’t know how or have an action plan (Theme 1). Most participants did not understand the benefits and risks of secondary-prevention exercise (Theme 2).

### Integration

#### PO1

Datasets agreed that participants were confident and understood how to perform their prescribed exercise programme. Qualitative data complemented this by showing that confidence and understanding were generally good, but were relatively lower for technically complex or resisted movements, and by highlighting physiotherapist demonstrations as a key facilitator of understanding.

#### PO2

Datasets partially agreed that participants were confident and understood how to adapt, progress or regress their exercises. Quantitative data indicated confidence, while qualitative findings showed that understanding was only partial and typically limited to adjusting repetitions. Qualitative data also complemented these findings by highlighting physiotherapists as key facilitators of understanding.

#### PO3

Datasets diverged regarding participants’ confidence and understanding of how to self-manage flare-ups with exercise. Although quantitative data indicated confidence, qualitative accounts revealed inconsistent or incomplete understanding, with some participants relying on non-exercise strategies.

#### PO4

Integration was not applicable, as only qualitative data were collected for PO4.

### Enactment

Interview findings generated PO-specific themes describing participant enactment and barriers/facilitators (online supplemental file 3, data S5).

#### PO1

Most participants reported completing their prescribed neck exercise programme but reducing or abandoning exercises that were complex (eg, four-point kneeling, scapular protraction and resisted exercises), painful or that were difficult to integrate into daily routines (Theme 1). Behavioural strategies such as piggybacking, prompts and routines supported completion (Theme 2). Symptom improvements reinforced engagement, whereas symptom worsening reduced completion (Theme 4).

#### PO2

Adaptation and progression of exercises were inconsistent, with some participants applying principles effectively and others reporting missed opportunities or no attempts to adjust (Theme 1). Fear of pain or harm and the time burden of additional repetitions acted as barriers (Theme 2). Symptom improvement, time-saving strategies (eg, increasing load while reducing repetitions), physiotherapist guidance and occasional use of exercise diaries facilitated progression (Theme 3).

#### PO3

Among participants who experienced flare-ups, few used exercise strategies (Theme 1). Participants who engaged with the workbook valued it and used it repeatedly to guide flare-up management (Theme 2).

#### PO4

As secondary-prevention exercise was unlikely to have been enacted by the time of discharge and is an optional, shared decision-making component, enactment was assessed only for general exercise. No participants started or resumed general exercise routines because of the intervention (Theme 1). Fear of pain or reinjury, time and mental well-being acted as key barriers, and participants typically lacked strategies to overcome them (Theme 1).

## Discussion

Fidelity varied across domains and intervention components. Although some prescribed exercise components were delivered successfully, overall delivery fidelity was below the predefined acceptable threshold, indicating that EPIC-Neck was not consistently delivered as intended. Prescribed exercises were generally well received and enacted by patients, although selectively. However, receipt and enactment for supporting exercise behaviours (flare-up management and general exercise) were weaker, a pattern that was consistent with poorer delivery of these components. Taken together, these findings suggest that EPIC-Neck requires optimisation of intervention content, training and implementation support before it can be considered ready for a definitive trial or wider delivery within routine NHS physiotherapy services.

### Patients engaged with exercise selectively

Most participants reported completing their prescribed exercise programme (PO1). This pattern of enactment was broadly aligned with the comparatively stronger receipt of PO1 and the relatively higher delivery scores for exercise-related and self-efficacy components. Although enactment was assessed qualitatively and not through quantitative adherence measures, these findings provide preliminary support for the underlying programme theory that behaviour change support may facilitate engagement with prescribed exercise. Future evaluations should include quantitative adherence measures to test this more robustly.^67^

However, enactment was selective, with participants favouring easier and less painful exercises, while more complex motor-control or resisted exercises were commonly reduced, avoided or under-dosed. This may reflect weaker receipt and delivery of components related to challenging exercises (eg, the delivery fidelity component ‘Improving tolerance’) and limited discussion of exercise benefits and safety.

Enactment also appeared shaped by convenience, with exercises that could be integrated into daily life more readily adopted. This aligns with evidence that habit formation and routine integration facilitate behavioural adoption.^68 69^ Poorer delivery of action planning (delivery fidelity component ‘Set a plan’) and problem-solving may have limited support for integrating less easily adopted exercises.

These findings highlight a tension between engagement and clinical effectiveness. The exercises with the least engagement are those that potentially provide the greatest reductions in pain and disability.^24^ This suggests that EPIC-Neck refinement should enhance strategies to improve engagement with more complex or resisted exercises while leveraging strategies to facilitate their integration into daily routines.

### Lower fidelity for supporting behaviours (PO3–4)

No participants started or resumed general exercise (PO4), and few experiencing flare-ups used appropriate exercise-based strategies (PO3). This contradicts the programme theory and may undermine the development of longer-term outcomes.^34 70 71^ Reduced enactment of these supporting exercise behaviours may partly reflect the inconsistent and/or incomplete understanding identified in the evaluation of receipt and the relatively poor delivery of intervention components designed to support PO3 and PO4 (<2). However, timing of assessment should also be considered. Interviews were conducted at discharge, after a mean intervention period of 16.9 weeks; however, PO4 may reasonably be introduced later in treatment, reducing the time available for participants to start or resume general exercise before the interview. Although this timeframe may have allowed initial attempts to be identified for some participants, it was unlikely to capture whether general exercise behaviours were sustained over time.^72^ Longer-term follow-up is therefore needed to determine whether participants initiate and sustain PO4 enactment over time.

These findings suggest a cross-domain pattern in which poorer delivery may help explain weaker receipt and enactment, although causal relationships between fidelity domains cannot be inferred. This is consistent with participants’ description of physiotherapists as central to developing their understanding. Although the sequential influence of delivery on receipt and enactment has been theorised, few studies have demonstrated this empirically, largely because most assess only a single fidelity domain rather than using multidimensional fidelity approaches.^36 41 42 73 74^ This supports the importance of improving delivery quality and evaluating whether delivery improvements are accompanied by stronger receipt and enactment in future work.

### Delivery barriers reflected clinician behaviour change and training design

Overall, few EPIC-Neck components were delivered acceptably (≥3), with a range of barriers explaining delivery challenges. However, poorer delivery was concentrated in components that were novel, complex, skill-intensive and less aligned with routine clinical practice, echoing findings from fidelity evaluations of other complex musculoskeletal interventions.^75–77^ Variability (indicated by a high SD in fidelity scores) was also evident within some components, particularly ‘Improving tolerance’, which likely reflected differences in physiotherapists’ confidence and clinical reasoning around progressive strengthening/loading, as well as patient-level variation in readiness, symptoms and tolerance. This suggests that physiotherapists need to develop new skills and change their own behaviour to integrate EPIC-Neck components into routine consultations.^78–82^

These delivery challenges align with findings from the evaluation of training fidelity. Although perceived confidence increased after training, physiotherapists did not appear to have developed sufficient skills to change their practice. Qualitative feedback highlighted the need for additional practice, individualised feedback and implementation support, mirroring reports from training evaluation of other complex musculoskeletal interventions.^83^ These findings suggest that the single-day, high-volume training model may have been insufficient for skill consolidation and behavioural adoption. This is consistent with evidence from staged training models, and feedback from physiotherapists during interviews, that training involving repeated practice, feedback and staged skill development may better support clinician behaviour change and delivery of complex interventions.^81 82 84^ However, the optimal format, duration and intensity of training required for EPIC-Neck warrant further investigation and should be designed with key stakeholders to ensure fit with the NHS context.

It is important to acknowledge that optimisation of training alone may not be sufficient to enhance delivery to an acceptable standard. Findings also indicated that factors relating to EPIC-Neck design and complexity influenced delivery. Optimisation should therefore examine whether the behavioural change components and overall programme structure can be simplified without undermining the intervention theory.^85^ Components with lower delivery, receipt or enactment fidelity, particularly social support, problem-solving and behaviours linked to PO2–PO4, may require redesign, clearer operationalisation or repositioning within the programme. Future refinement should clarify which components are essential versus adaptable, reduce avoidable cognitive and practical burden and specify how behavioural change components can be tailored while preserving the intended mechanisms of action.^85–87^

### Strengths and limitations

A key strength of this study was its mixed-methods design, enabling a deeper evaluation of fidelity than questionnaires alone and allowing relationships across fidelity domains to be explored. Conducting the study in routine NHS settings with real patients and physiotherapists provided insight into actual delivery and enactment rather than hypothetical implementation. Evaluation across multiple fidelity domains provided valuable evidence on how domains may sequentially interact.

Several limitations should be considered. Beyond the initial intervention mapping process, no further independent assessment of design fidelity was undertaken. Enactment was assessed qualitatively at discharge, with no quantitative or objective measures, limiting assessment of longer-term behaviour maintenance. However, interviews provided insight into enactment, adaptation, barriers and facilitators. Future evaluations should consider combining qualitative methods with quantitative measures of adherence or enactment, such as the Adherence To Exercise for Musculoskeletal Pain Tool (ATEMPT).^67^

The receipt questionnaires and delivery-fidelity checklist were purpose-designed for EPIC-Neck and NIH-BCC fidelity domains but were not externally validated. Although the delivery-fidelity checklist was reviewed by CG, final scoring was undertaken by JP, introducing potential bias. Calibration was conducted with a second researcher before single coding, but no formal inter-rater reliability statistic was calculated.

Physiotherapists were aware that appointments were being audio-recorded, which may have altered behaviour and overestimated delivery fidelity, although recording enabled direct assessment rather than reliance on self-report.^36^ Finally, interviews were conducted and analysed by JP, who had existing professional relationships with some physiotherapists, and no formal independent double-coding was undertaken. Independent interviewing and coding would have strengthened the evaluation, although credibility was supported through structured topic guides, confidentiality procedures, team review of coding decisions and an audit trail.

## Conclusion

This multidimensional fidelity evaluation showed that the fidelity of the EPIC-Neck intervention varied across domains and components. Receipt and enactment were relatively stronger for performing prescribed neck exercises than for longer-term condition-management behaviours (PO2-4), although enactment was selective and declined for more challenging exercises. These patterns appeared to be linked, at least in part, to suboptimal delivery of several components that required physiotherapists to develop new skills and change existing practice.

EPIC-Neck requires optimisation before progression to a definitive trial. Refinements should focus on strengthening physiotherapist training and support, including candidate strategies such as staged learning, additional practice and regular feedback, to help clinicians develop and maintain the skills required for delivery. Optimisation should also consider simplifying the intervention design and clarifying essential versus adaptable components, while preserving the intended mechanisms of action. Repeat fidelity evaluation is needed before progressing to a definitive trial evaluating long-term effectiveness and engagement.

## Supplementary material

10.1136/bmjopen-2026-121944 online supplemental file 1

10.1136/bmjopen-2026-121944 online supplemental file 2

10.1136/bmjopen-2026-121944 online supplemental file 3

10.1136/bmjopen-2026-121944 online supplemental file 4

10.1136/bmjopen-2026-121944 online supplemental file 5

10.1136/bmjopen-2026-121944 online supplemental file 6

## Data Availability

Data are available upon reasonable request.
