# Supplementary material for: Multidimensional fidelity of the EPIC-Neck exercise programme for chronic non-specific neck pain: a UK feasibility trial process evaluation
Source: BMJ Open. 2026 Oct 20;16(10):e121944. doi: 10.1136/bmjopen-2026-121944 (PMC13684230; doi:10.1136/bmjopen-2026-121944)
Supplement: Supplementary file 1 [file bmjopen-16-10-s001.pdf]

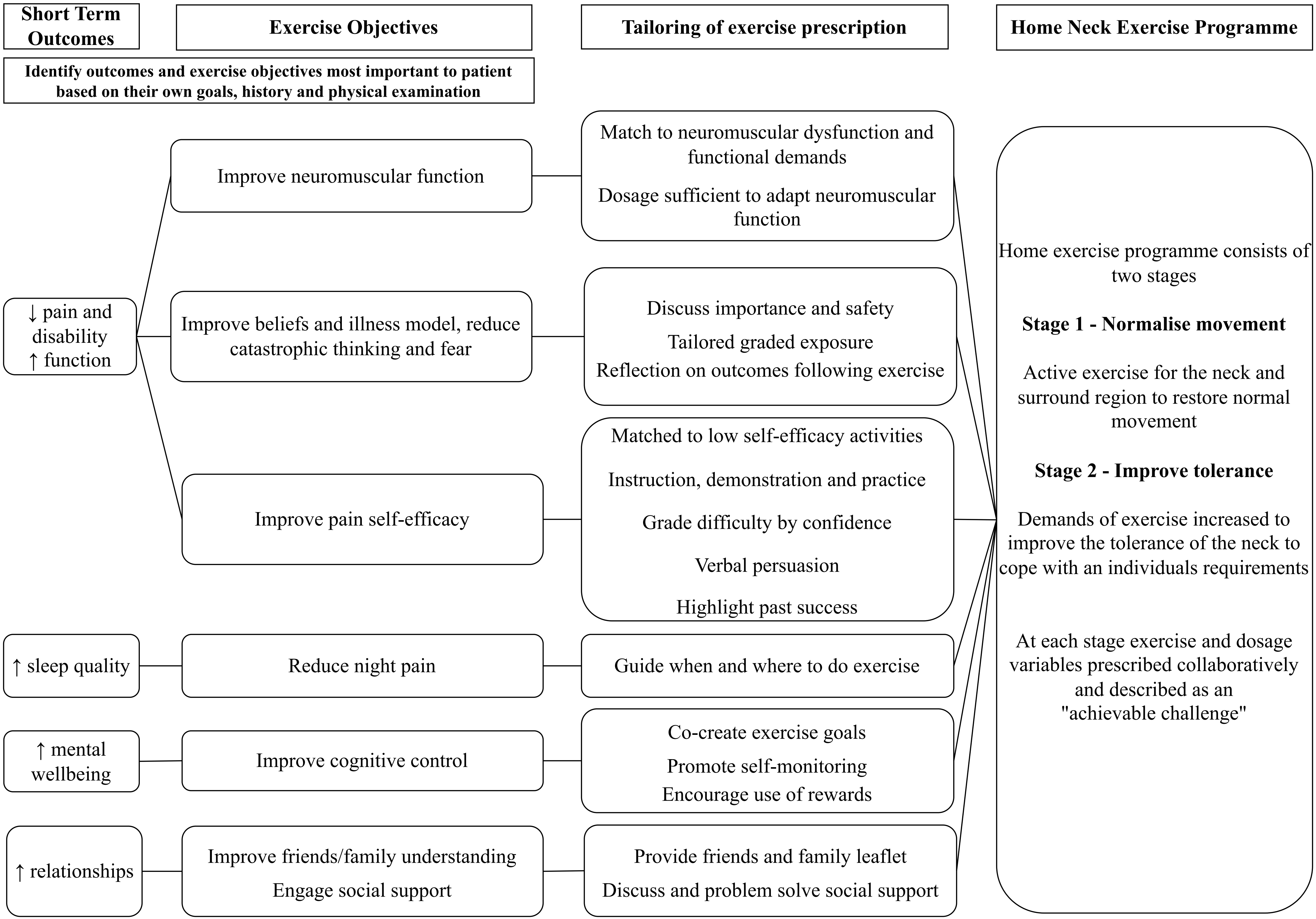

Short Term Outcomes
Exercise Objectives
Tailoring of exercise prescription
Home Neck Exercise Programme
Identify outcomes and exercise objectives most important to patient based on their own goals, history and physical examination
↓ pain and disability ↑ function
Improve neuromuscular function
Match to neuromuscular dysfunction and functional demands
Dosage sufficient to adapt neuromuscular function
Improve beliefs and illness model, reduce catastrophic thinking and fear
Discuss importance and safety
Tailored graded exposure
Reflection on outcomes following exercise
Improve pain self-efficacy
Matched to low self-efficacy activities
Instruction, demonstration and practice
Grade difficulty by confidence
Verbal persuasion
Highlight past success
↑ sleep quality
Reduce night pain
Guide when and where to do exercise
↑ mental wellbeing
Improve cognitive control
Co-create exercise goals
Promote self-monitoring
Encourage use of rewards
↑ relationships
Improve friends/family understanding
Engage social support
Provide friends and family leaflet
Discuss and problem solve social support
Home exercise programme consists of two stages
Stage 1 - Normalise movement
Active exercise for the neck and surround region to restore normal movement
Stage 2 - Improve tolerance
Demands of exercise increased to improve the tolerance of the neck to cope with an individuals requirements
At each stage exercise and dosage variables prescribed collaboratively and described as an "achievable challenge"
