# Supplementary material for: Multidimensional fidelity of the EPIC-Neck exercise programme for chronic non-specific neck pain: a UK feasibility trial process evaluation
Source: BMJ Open. 2026 Oct 20;16(10):e121944. doi: 10.1136/bmjopen-2026-121944 (PMC13684230; doi:10.1136/bmjopen-2026-121944)
Supplement: Supplementary file 2 [file bmjopen-16-10-s002.pdf]

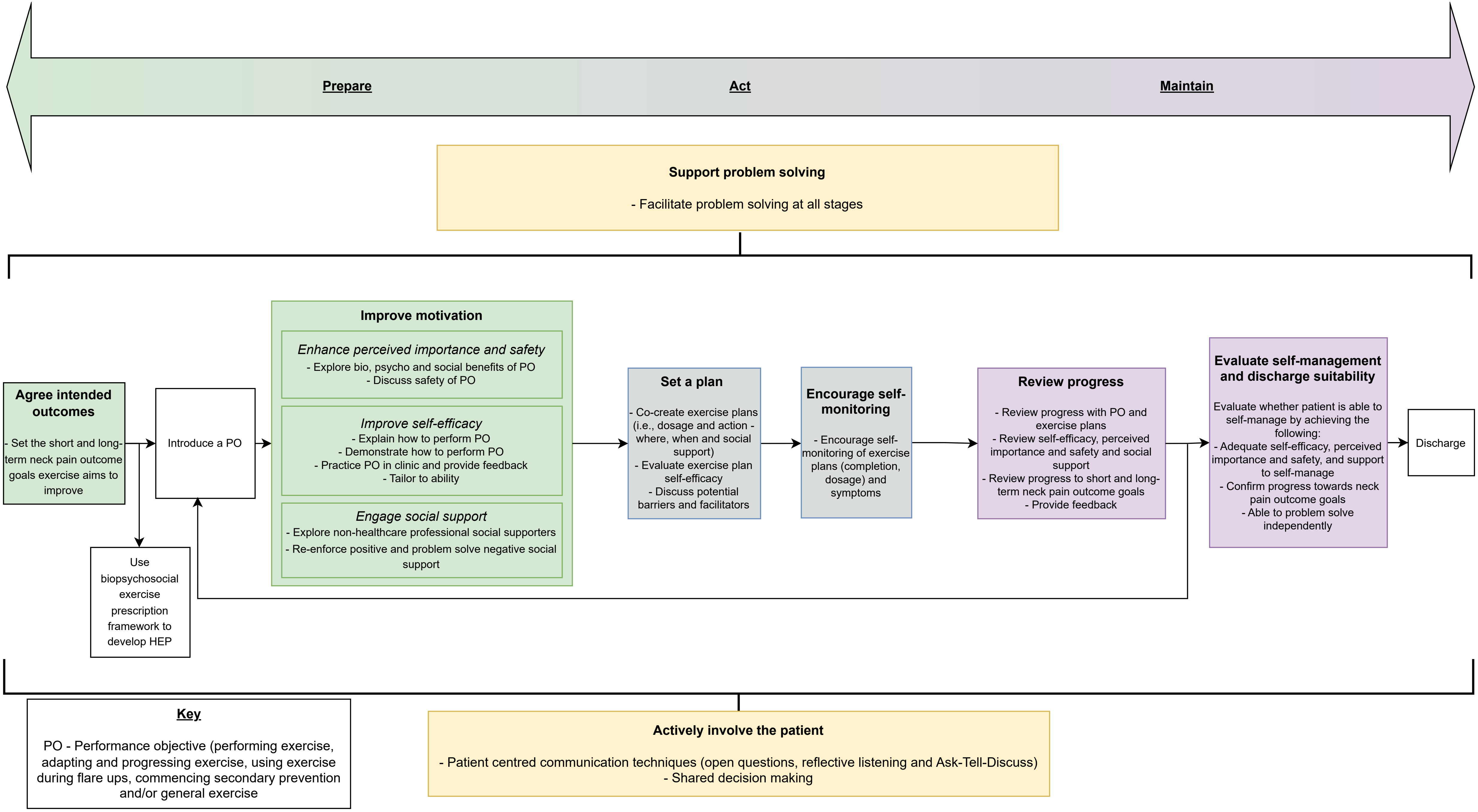
Prepare
Act
Maintain
Support problem solving
- Facilitate problem solving at all stages
Agree intended outcomes
- Set the short and long-term neck pain outcome goals exercise aims to improve
Introduce a PO
Use biopsychosocial exercise prescription framework to develop HEP
Improve motivation
Enhance perceived importance and safety
- Explore bio, psycho and social benefits of PO
- Discuss safety of PO
Improve self-efficacy
- Explain how to perform PO
- Demonstrate how to perform PO
- Practice PO in clinic and provide feedback
- Tailor to ability
Engage social support
- Explore non-healthcare professional social supporters
- Re-enforce positive and problem solve negative social support
Set a plan
- Co-create exercise plans (i.e., dosage and action - where, when and social support)
- Evaluate exercise plan self-efficacy
- Discuss potential barriers and facilitators
Encourage self-monitoring
- Encourage self-monitoring of exercise plans (completion, dosage) and symptoms
Review progress
- Review progress with PO and exercise plans
- Review self-efficacy, perceived importance and safety and social support
- Review progress to short and long-term neck pain outcome goals
- Provide feedback
Evaluate self-management and discharge suitability
Evaluate whether patient is able to self-manage by achieving the following:
- Adequate self-efficacy, perceived importance and safety, and support to self-manage
- Confirm progress towards neck pain outcome goals
- Able to problem solve independently
Discharge
Key
PO - Performance objective (performing exercise, adapting and progressing exercise, using exercise during flare ups, commencing secondary prevention and/or general exercise
Actively involve the patient
- Patient centred communication techniques (open questions, reflective listening and Ask-Tell-Discuss)
- Shared decision making
