# Supplementary material for: Multidimensional fidelity of the EPIC-Neck exercise programme for chronic non-specific neck pain: a UK feasibility trial process evaluation
Source: BMJ Open. 2026 Oct 20;16(10):e121944. doi: 10.1136/bmjopen-2026-121944 (PMC13684230; doi:10.1136/bmjopen-2026-121944)
Supplement: Supplementary file 3 [file bmjopen-16-10-s003.pdf]

Table S1 - Physiotherapist barriers and facilitators and training strategies

Determinant	Determinant Definition	Workshop	Literature	Strategy
Digital literacy	Physiotherapist poor digital literacy meaning difficulty with using web dashboard to set patients up on EPIC-Neck app	-	(1)	Provide a walkthrough (1 - written with pictures and 2 - video) of how to use EPIC-Neck dashboard to set up participants and create/modify exercise programmes.
Skills	Physiotherapists lacking skills in delivering behaviour change techniques, person-centred counselling, and assessing/prescribing exercise for neck pain	YES	(1-7)	Practice delivering key aspects of the intervention during training. Produce a physiotherapy manual explaining how to deliver the intervention.
Existing practice	A physiotherapist existing practice and routine being different to the EPIC-Neck programme	YES	(2, 4, 6)	Arrange for peer support or champions to provide feedback and monitoring on delivery. Prompt physios to create a plan to practise components of EPIC-Neck with other chronic musculoskeletal patients. Prompt identification of 1 technique they are uncomfortable with and outline when and how often they will practice it. Prompt practising the components of EPIC-Neck that are different to their existing routine with all chronic MSK patients who may not be on the trial. Provide summary crib sheets to be visible during clinic to prompt delivery. Encourage using workbook in clinic to support discussing and delivery of change techniques. Provide summary crib sheets to be visible during clinic to prompt delivery. Encourage using workbook in clinic to support

Determinant	Determinant Definition	Workshop	Literature	Strategy
				discussing and delivery of change techniques. Send monthly bulletins updating progress and summarising 1 or 2 key points of intervention.
Patient complexity	Complex or challenging patients making assessment and application of EPIC-Neck more difficult	YES	(3)	Encourage physiotherapist to prioritise one or two key points for complex patients. Advised it is OK to not complete whole intervention in one go, and it is designed to be delivered over a series of appointments. EPIC-Neck is flexible and can be individualised to suit physiotherapist and patient needs. Provide summary crib sheets to be visible during clinic to prompt delivery. Encourage using workbook in clinic to support discussing and delivery of change techniques. Send monthly bulletins updating progress and summarising 1 or 2 key points of intervention.
Memory	Physiotherapist forgetting how or when to deliver the intervention	YES	-	EPIC-Neck programme designed to limit the amount the physiotherapist actually needs to do in clinic. Physiotherapists can utilise workbook where possible. Provide summary crib sheets to be visible during clinic to prompt delivery. Encourage using workbook in clinic to support discussing and delivery of change techniques. Send monthly bulletins updating progress and summarising 1 or 2 key points of intervention.
Too much information	Large volume of content and tasks	-	(3-5, 7)	Limit the amount the physiotherapist actually needs to do and utilise workbook where possible. Focus training on the key components within the Biopsychosocial Framework and exercise behaviour change model. Provide summary crib sheets to be visible during clinic to prompt delivery. Encourage using workbook in clinic to support discussing and delivery of change

Determinant	Determinant Definition	Workshop	Literature	Strategy
				techniques. Send monthly bulletins updating progress and summarising 1 or 2 key points of intervention.
Knowledge	Physiotherapists existing knowledge in behaviour change techniques and exercise prescriptions for neck pain	YES	(1-7)	Explain the proposed health benefits of EPIC-Neck versus usual exercise care. Explain the proposed social and environmental benefits of EPIC-Neck versus usual exercise care both for the participant and the healthcare system. Training and therapist workbook explaining how to deliver the intervention.
Burnout	Physiotherapist physical and emotional exhaustion due to work and personal life pressures	YES	-	Provide resources for physiotherapists for further support with mental well-being.
Emotional response	Physiotherapist feels embarrassed, anxious, scared, frustrated etc. when delivering components of EPIC-Neck	YES	(2, 6, 7)	Encourage physiotherapists to start off asking simple questions relating to social and mental well-being aspects. As confidence improves, they can explore in more depth. Explain its normal for physiotherapists to initially feel embarrassed, anxious, scared or frustrated when delivering components of the EPIC-Neck programme such as discussions around mental well-being and social support. Explain that the patient group who developed this content felt these were acceptable and important to discuss. Provide resources for further support with mental well-being.
Self-efficacy	Physiotherapist confidence in delivering EPIC-Neck	YES	(4, 6, 7)	Link aspects of the intervention to skills they already do well. Practice delivering key aspects of the intervention during training. Tell the physios they can successfully deliver the intervention.

Determinant	Determinant Definition	Workshop	Literature	Strategy
				Training and therapist workbook explaining how to deliver the intervention. Video demonstrations and in person demonstrations of how to deliver key aspects of the intervention.<br>Within training session include a problem-solving section. Ask physiotherapist to identify some barriers to delivering the EPIC-Neck intervention and then identify solutions. Work in pairs/groups for peer support in idea generation.
Perception of benefit	Physiotherapists perception of the benefit of EPIC-Neck	-	(2, 4-7)	Explain the proposed emotional benefits of EPIC-Neck versus usual exercise care. Explain the proposed health benefits of EPIC-Neck versus usual exercise care. Explain the proposed social and environmental benefits of EPIC-Neck versus usual exercise care both for the participant and the healthcare system.
Professional identity	Physiotherapists perception of their role and conflicts with aspects of the EPIC-Neck intervention	YES	(4-7)	Education that patient group felt biggest barrier was not being heard or listened to. Encouraging the use of patient centred counselling. Encourage discussing cases with peers who already utilise mental wellbeing/social evaluations
Communication barriers	Communication barriers between clinician and patient	YES	-	Encourage physiotherapist to direct the participant to the workbook and encourage them to use the support of family members when reading it. Encourage the use of interpreters in line with service guidelines. Use person centred counselling style to improve communication.
Environment	Issues with the space where EPIC-Neck will be delivered e.g., not enough space, too noisy	-	(5, 6)	Intervention designed to be delivered in routine clinical environment.

Determinant	Determinant Definition	Workshop	Literature	Strategy
Resources	Inadequate access and quality of resources to support intervention delivery	YES	(3, 5)	Provide links for any online resources. Provide the workbooks. Limiting the requirements for equipment not already routinely available in NHS clinical practice.
Tailoring	Ability to tailor the EPIC-Neck intervention to suit physiotherapist, organisation, and environmental factors	-	(5)	EPIC-Neck intervention designed to be delivered flexibly to suit site, person and organisational needs.
Time	Limited time and multiple other priorities	YES	(1, 4-7)	Encourage physiotherapists to prioritise most important outcomes/mediators/BCTs if limited on time. Can individualise EPIC-Neck intervention to meet time constraints. Can deliver other components at other appointments. Provide summary crib sheet to assist with prioritising key components
Organisation/social norm	Differences between EPIC-Neck intervention and organisations and colleagues' normal practice	-	(2, 4, 6)	Ask line managers to support staff with change in delivery style. Utilise "champion" roles within services to encourage and support staff with delivery. Encourage peer to peer feedback or discussions throughout the project
Organisational support	Availability of support from an organisation or colleagues in delivering EPIC-Neck	-	(6)	Ask line managers to support staff with change in delivery style. Utilise "champion" roles within services to encourage and support staff with delivery. Encourage peer to peer feedback or discussions throughout the project. Suggest a WhatsApp group/email chain for staff delivering EPIC-Neck for troubleshooting and support

Determinant	Determinant Definition	Workshop	Literature	Strategy
Patients' expectations, beliefs, and engagement	Patients' expectations, beliefs, and engagement	YES	(3, 6, 7)	Education that patient group felt biggest barrier was not being heard or listened to. Encouraging the use of patient centred counselling. Intervention designed to specifically evaluation patient barriers, beliefs, expectations etc.
Therapeutic alliance	Therapeutic alliance between the physiotherapist and patient	YES	(6)	Education that patient group felt biggest barrier was not being heard or listened to. Encouraging the use of patient centred counselling. Intervention delivered using patient centred counselling style.

Abbreviations: HCP: Healthcare Professional

1. O'Shea A, Drennan J, Littlewood C, Slater H, Sim J, McVeigh JG. Barriers and facilitators related to self-management of shoulder pain: a systematic review and qualitative synthesis. Clin Rehabil. 2022;36(11):1539-62.

2. Shah S, Prakash V. Limited evidence exists on determinants of physiotherapists' adherence to high value interventions in nonspecific low back pain management: A scoping review. Musculoskeletal Care. 2022;20(3):442-53.

3. Toomey E, Matthews J, Hurley DA. Using mixed methods to assess fidelity of delivery and its influencing factors in a complex self-management intervention for people with osteoarthritis and low back pain. BMJ Open. 2017;7(8):e015452.

4. Babatunde FO, MacDermid JC, MacIntyre N. A therapist-focused knowledge translation intervention for improving patient adherence in musculoskeletal physiotherapy practice. Arch Physiother. 2017;7(1):1.

5. Daw P, Withers TM, van Zanten J, Harrison A, Greaves CJ. A systematic review of provider-and system-level factors influencing the delivery of cardiac rehabilitation for heart failure. BMC Health Serv Res. 2021;21(1):1267.

6. van Dijk H, Koke AJA, Elbers S, Mollema J, Smeets R, Wittink H. Physiotherapists Using the Biopsychosocial Model for Chronic Pain: Barriers and Facilitators-A Scoping Review. Int J Environ Res Public Health. 2023;20(2):1634.

7. Gervais-Hupe J, Filleul A, Perreault K, Hudon A. Implementation of a biopsychosocial approach into physiotherapists' practice: a review of systematic reviews to map barriers and facilitators and identify specific behavior change techniques. Disabil Rehabil. 2023;45(14):2263-72.

TRAINING FIDELITY

Table S2 - Physiotherapist confidence delivering components of EPIC-Neck intervention

	EPIC-Neck – Pre-training (n=9)	EPIC-Neck – Post-training (n=9)	Usual Exercise Care (n=9)
**EPIC-Neck component**	Median (IQR)	Median (IQR)	Median (IQR)
**Exercise prescription**			
Assessing neuromuscular dysfunction	4.0 (1.0)	4.0 (0.0)	4.0 (0.0)
Prescribing for neuromuscular dysfunction	4.0 (0.0)	4.0 (0.0)	4.0 (0.0)
Determining exercise dosage	4.0 (1.0)	4.0 (0.0)	4.0 (0.0)
Progressing exercise	4.0 (1.0)	4.0 (1.0)	4.0 (0.0)
Regressing exercise	4.0 (0.0)	4.0 (0.0)	4.0 (0.0)
Assessing catastrophic thinking and fear avoidance	3.0 (1.0)	4.0 (0.0)	4.0 (1.0)
Prescribing for catastrophic thinking and fear avoidance	3.0 (2.0)	4.0 (1.0)	4.0 (1.0)
Assessing pain self-efficacy	3.0 (1.0)	4.0 (0.0)	4.0 (1.0)
Prescribing for pain self-efficacy	2.0 (1.0)	3.0 (1.0)	4.0 (1.0)
Assessing sleep	3.0 (1.0)	4.0 (0.0)	4.0 (1.0)
Prescribing for sleep	2.0 (1.0)	4.0 (1.0)	3.0 (1.0)
Assessing mental wellbeing	3.0 (0.0)	4.0 (0.0)	4.0 (0.0)
Prescribing for mental wellbeing	2.0 (1.0)	3.0 (1.0)	3.0 (2.0)
Assessing social function and relationships	3.0 (2.0)	4.0 (0.0)	4.0 (1.0)
Prescribing for social function and relationships	1.0 (1.0)	3.0 (1.0)	3.0 (0.0)
Overall confidence	3.0 (1.0)	4.0 (1.0)	4.0 (0.0)
**Exercise prescription total**	3.0 (2.0)	4.0 (1.0)	4.0 (1.0)
**Performance objectives**			
Supporting adherence to neck exercise	3.0 (1.0)	4.0 (0.0)	4.0 (1.0)
Supporting independent exercise modification and progression/regression	3.0 (1.0)	4.0 (0.0)	4.0 (0.0)
Supporting flare-up management	4.0 (1.0)	4.0 (0.0)	4.0 (1.0)

	EPIC-Neck – Pre-training (n=9)	EPIC-Neck – Post-training (n=9)	Usual Exercise Care (n=9)
Discussing pros/cons of secondary prevention	3.0 (1.0)	4.0 (1.0)	4.0 (1.0)
Supporting general physical activity	3.0 (1.0)	4.0 (0.0)	4.0 (0.0)
**Performance objectives total**	3.0 (1.0)	4.0 (0.0)	4.0 (1.0)
**Behaviour change**			
Discussing physical benefits of neck exercise	4.0 (1.0)	4.0 (0.0)	4.0 (0.0)
Discussing psychological/emotional benefits of neck exercise	3.0 (2.0)	4.0 (1.0)	4.0 (0.0)
Discussing social/environmental benefits of neck exercise	2.0 (2.0)	4.0 (1.0)	4.0 (1.0)
Setting/reviewing outcome goals	3.0 (0.0)	4.0 (0.0)	4.0 (1.0)
Setting/reviewing exercise adherence and dosage	4.0 (1.0)	4.0 (0.0)	4.0 (1.0)
Providing feedback	4.0 (1.0)	4.0 (0.0)	4.0 (1.0)
Creating action plans	3.0 (2.0)	4.0 (1.0)	4.0 (0.0)
Facilitating independent problem-solving	3.0 (0.0)	4.0 (0.0)	4.0 (0.0)
Grading exercise	3.0 (2.0)	4.0 (1.0)	4.0 (0.0)
Engaging social support	2.0 (1.0)	3.0 (1.0)	4.0 (2.0)
Using person-centred communication	3.0 (2.0)	3.0 (2.0)	4.0 (2.0)
**Behaviour change total**	3.0 (2.0)	4.0 (1.0)	4.0 (0.0)
**Overall total**	3.0 (2.0)	4.0 (1.0)	4.0 (1.0)

Abbreviations: IQR – Inter quartile range

Footnote: 1 = Not confident, 2 = Slightly confident, 3 = Somewhat confident, 4 = Fairly confident, 5 = Completely confident

Data S1 - Training satisfaction survey findings

Exercise training

Question 1.1 - Did the training cover an appropriate amount of material?

8/9 attendees (89%) felt the content was appropriate amount of material in relation to exercise covered in the training. 1 felt there was too little and commented:

> *"A section on the actual exercises used in the study would have been useful."*

Additional comments:

> *"Role plays very helpful. Could perhaps do with even more practice to consolidate further."*
>
> *"A lot to cover, perhaps a second day seeing more exercises and full physical assessment maybe useful. Otherwise very good."*
>
> *"Good content, well delivered, simple concepts and application"*

Question 1.2 - Rate how useful the training was to enable you to assess and prescribe exercise for people with chronic non-specific neck pain using the EPIC-Neck programme.

8/9 attendees (89%) felt the training was good (n=4) or excellent (n=4) in its usefulness in enabling their ability to assess and prescribe exercise. 1 attended rated it as fair.

Behaviour change training

Question 2.1 - Did the training cover an appropriate amount of material?

9/9 attendees (100%) felt there was an appropriate amount of material in relation to behaviour change covered in the training.

Question 2.2 - Rate how useful the training was to enable you to use behaviour change techniques as part of the EPIC-Neck programme.

9/9 attendees (100%) felt the training was good (n=6) or excellent (n=3) in its usefulness in enabling their ability to use behaviour change techniques.

General feedback

Question 3.1 - What are your thoughts on the mixture of learning methods used?

All comments were positive towards the mixture of learning methods. Selected examples:

> *"Really useful using case studies and role play additionally to formal teaching"*
>
> *"Good mixture of discussion, teaching, practical elements and role play. Could benefit from further role play and practical elements. Perhaps something to bring everything together at the end."*

Question 3.2 - Were you satisfied with the delivery of the training?

9/9 attendees (100%) were satisfied (n=3) or very satisfied (n=6) with the training.

Question 3.3 - What did you enjoy most about the training?

There were positive comments about the training include the learning environment, practical components, attendee numbers and opportunities for discussion:

“The practical part with specific assessment techniques.”

“Time to discuss implementation of the program and how we will approach this in practice.”

Question 3.4 - What did you enjoy least about the training?

Elements of the training people did not enjoy were around the time spent on exercise teaching, and volume of training in a day. Some attendees felt more emphasis and time should have been spent on the exercises themselves:

“Would have liked more info on the exercises.”

“A lot to cover, perhaps a second day seeing more exercises and full physical assessment maybe useful. Otherwise very good.”

“All good. Challenge and enjoyed practice. Needs a brief run through of an example patient to observe full flow”

Question 3.5 - Is there anything we could improve about the training?

Suggestions:

“Exercises used in the study. Another session would be useful before the start of the study to do more practice.”

“Even more practical and opportunity to practice exercises as some of these are unfamiliar/ not ones I typically use. Something to bring it all together at the end e., g video of how an assessment may look in practice”

“More time on practical aspects of the assessment and exercises.”

“More time spent on practicing, demonstration, explanation and coaching of specific exercises would be useful”

“More specific problem-based learning with context or including personal experiences.”

“A lot to cover, perhaps a second day seeing more exercises and full physical assessment maybe useful. Otherwise very good.”

Data S2 - Training fidelity interview themes and illustrative quotes

Theme 1 – Physiotherapists reported that training enhanced their capability (skills, knowledge, and confidence) but they did not feel fully capable of applying the programme in practice.

Physiotherapists valued the training and reported gaining new skills and knowledge, but many did not feel fully prepared to apply the approach in clinical practice, often describing uncertainty and lack of confidence once delivery began.

> *"I think at the end of the training I was like 'Woah…I'm not sure I know what I'm doing'…so I was shying away like 'ohh give that participant to someone else.''" PT6 - 0-2 years' experience*

Theme 2 – Practical examples and rehearsal supported capability building but needed expansion

Role-play, case discussions, and practical components were valued for helping to translate theory into practice. However, physiotherapists wanted to see real-life examples of both optimal and suboptimal delivery, and to spend more time practising exercises and behavioural strategies.

> *"You could have a mock-up of right this is one… what's not going well here? How might you have done it? That might be good. Or a good example to see something done well" PT5 - 6-10 years' experience*

Theme 3 – Support from experts and peers facilitated capability development, but individualised feedback on delivery was missing and essential

Physiotherapists valued opportunities to learn from experts and peers, describing case discussions and informal supervision as helpful for clarifying concepts and problem-solving. However, these opportunities were limited and often ad hoc. Engagement with peer support also varied, with some finding it motivating while others struggled to sustain participation. Across accounts, a consistent gap was the lack of individualised feedback on their own delivery with patients. Physiotherapists felt this feedback was essential for building confidence, ensuring fidelity, and reinforcing correct application of the programme in practice.

> *"I did find the group chat quite helpful as well. Once you had your experience, you could feedback and you could look at others' experiences. That helped but as we felt a little bit more confident, and it was running more smoothly it petered out, so I didn't use it as much" PT2 - 11-20 years' experience*

> *"If I'd got feedback pretty soon after appointments it would have validated what I was doing. I thrive off that quick criticism or feedback. It would have made me more confident, but it would also stop me from doing things just because it's the path of least resistance for me…" PT3 - 0-2 years' experience*

Theme 4 – High content volume and limited time reduced consolidation and confidence

The single-day format and breadth of material left some feeling rushed or overwhelmed. Participants consistently suggested spreading content across sessions or providing staged learning.

> *"I felt like I had to kind of switch a lot of things around all at once and I couldn't make that change quickly… It needs to spread out, so you have a chance to*

implement some of it. Then you receive more and then you implement more.” PT1 - 6-10 years' experience

Theme 5 – Limited practice opportunities and gaps between training and delivery constrained capability development

Long delays before seeing patients and limited opportunity to apply training in real clinical settings weakened retention and preparedness. Participants suggested immediate practice opportunities with routine patients with transferable concepts.

> *“You just need to see a lot of patients…maybe we could implement things with routine patients from an earlier point rather than waiting for a research patient… because a lot of the assessment, communication side of things, was completely transferable to anyone.” PT1 - 6-10 years' experience*

Theme 6 – Self-directed learning and refresher training were recognised as useful but rarely prioritised

While physiotherapists acknowledged that self-directed learning, reflection, and refresher sessions could reinforce skills and maintain confidence, these were rarely undertaken. Competing clinical priorities and limited time meant learning outside the initial training day, or patient contact was deprioritised.

> *“Having that time to read it and go back to the basics. I think I should have taken more time, but that would have involved carving out some time…you also emailed about a refresher, but at the time I had so much coming at me I was like ‘hmm I’ll pass on that’” PT6 - 0-2 years' experience*

DELIVERY FIDELITY

Table S3 - Delivery fidelity checklist

Interventional Techniques	Perfect - 5	Acceptable - 3	Scoring Notes
<u>**Intervention components to maximise engagement**</u>			
**Agree outcome goals:** The short and long-term neck pain outcome goals exercise aims to improve are determined *(Max score)*<br>***Notes:*** *Score 0 at follow-up if no new goals are set-up if no new goals are set*			
1. Set short (≤3 months) and long-term (12 months) outcome goals linked to current restrictions or limitations.	Goals verbally agreed for 3 and 12 months<br>Goals verbally and explicitly linked to patient's restrictions/limitations.<br>Goals set collaboratively using ASK-TELL-DISCUSS. DISCUSS is essential for a 5.	Goals verbally agreed and linked to restrictions/limitations.<br>Physio suggests goals before asking patient's preference.	Physio-suggested goals not explicitly linked to restrictions → max 2.<br>Goals not verbally agreed & explicitly linked → max 2.
2. Ensure outcome goals are measurable.	Goals are measurable and include clear targets (e.g., "walk 20 minutes without pain").	Goals are measurable but lack specific targets (e.g., "walk more").	
3. Encourage recording outcome goals in the workbook.	Physio physically shows the patient where in the workbook/wall chart to record the goals	Physio mentions recording but does not physically direct.	
**Actively involve the patient:** Patient centred communication style used throughout to encourage patients' active involvement in the appointment and decision making *(Mean score)*			
1. Predominant use of open questions to elicit information.	Predominant use of open questions.	Some open questions but reliance on closed questions.	Only 1–2 open questions → max 2.

Interventional Techniques	Perfect - 5	Acceptable - 3	Scoring Notes
Closed questions are permitted where necessary.	Closed questions only when necessary.	Open question immediately followed by closed before patient responds.	
2. Use of Ask-Tell-Discuss techniques to help with information exchange. Needs to be all at the same time when discussing a concept/idea/barrier.	Full ASK-TELL-DISCUSS used in a single exchange.<br>ASK and DISCUSS prompt use open questions.	ASK-TELL used but no discussion.	TELL only → max 1.<br>DISCUSS prompted with closed question → max 4.
3. Use of reflective listening e.g., repetition, paraphrasing and reflecting feelings	Reflective listening immediately after important information is provided.<br>Includes summarisation at key points.	Summarisation/Reflective listening used intermittently, but key points may be missing.	
4. Shared decision making used throughout appointment	Open questions used to prompt patient's input in decision-making or to confirm a physio decision is acceptable (e.g., physio sets dosage and asked for patient opinion [open question])	Closed questions used to confirm acceptance of physio's decision. To score 3 must be used regularly throughout the appointment. Physio decision making based on information provided by patient throughout appointment or prior appointment.	Fully physio-led decisions (no patient input) → score 0.<br>1–2 closed-question confirmations ("is that ok?") → max 2.
**Improve motivation to perform exercise (PO1) - Enhance perceived importance and safety** - Physiotherapist helps to develop /enhance a patient's understanding of the importance and safety of performing exercise *(Max score)*			
1. Explore and seek to enhance the patient's perception of the bio, psycho, and social (relationships & social activities) benefits of	Uses ASK-TELL-DISCUSS to explore that patient's perception of bio, psycho and social benefits.	Physio states the bio, psycho and social benefits of exercise. All 3 essential to score a 3.	Only bio discussed → max 1.<br>Two of the three domains discussed → max 2.

Interventional Techniques	Perfect - 5	Acceptable - 3	Scoring Notes
performing exercise and of reducing neck pain using Ask-Tell-Discuss.	Discuss after telling is essential to score a 5.<br>Physio links benefits back to key findings from history or patient goals		
2. Discuss /explain the safety of performing exercise.	Use of ASK-TELL-Discuss to explore safety of exercise.<br>Discuss is essential to score a 5.	States that exercises are safe but does not explore patient concerns or thoughts in relation to safety.	
**Improve motivation to perform exercise (PO1) - Improve self-efficacy** - Physiotherapists uses techniques to help improve a patient's self-efficacy for performing exercise *(Mean score)*<br>***Notes:** Score NA if no new or modified exercises; NA if review identifies no issues.*			
1. Provide accurate verbal or written instruction on how to perform exercise.	Instructions clearly understandable from audio.<br>Exercises are explicitly part of programme and formally "set."	Instructions provided but may still be unclear from audio.	Audio quality prevents evaluation → score 3.
2. Physio to physically demonstrate how to perform exercise.	100% of prescribed exercises demonstrated by physiotherapist. Acceptable to use video to demonstrate exercises.	The majority (≥66%) of exercises prescribed demonstrated by the physiotherapist.	Printouts/static images used → max 2.
3. Patient practices exercise in clinic and physio provides feedback.	Patient practices all exercises (full dosage not required).<br>Detailed feedback provided with reinforcement or corrections.	Patient practices most exercises (≥66%).<br>Brief feedback given (e.g., "good job").	No feedback → max 2.

Interventional Techniques	Perfect - 5	Acceptable - 3	Scoring Notes
4. Tailor exercise to patients' ability and goals.	Exercise position/action linked to goal and physio explicitly states/demonstrates how<br>Exercises should be achievable for patients to perform.<br>Physio may change position/exercise if too difficult or too easy.<br>Sense checked with patient that exercise is suitable for ability through questioning (open or closed permitted)	Exercise appears reasonable for patients' ability. If states dosage to be manipulated to be a challenge, then score 3.<br>Must be practiced in clinic to score a 3.<br>Physio does not sense check with patient<br>Physio does not explicitly and verbally link to goals	No practice → score 0.<br>No practice but gives reps/adjustments as "challenge" → max 1.<br>No goals set → max 4.
5. Direct to Module 2 of the workbook for more detail.	Physio physically shows the patient the exercise library highlighting exercise within their programme.	Physio states there is an exercise library, and exercises can be found in there.	
**Improve motivation to perform exercise (PO1) - Engage social support** - Physiotherapists supports patient in engaging social support to assist with performing exercise programme *(Max score)*<br>***Notes:*** *Social-support discussion must relate to the exercise programme (not overall neck-pain management) → otherwise score 0.*			
1. Explore existing social support (both positive and negative).	Use of open questioning to explore what social support potentially exists to support with the exercise programme (can be when exercise prescription or at review appointments)	Use of closed questioning to explore social support with exercise (can be when exercise prescription or at review appointments)	

Interventional Techniques	Perfect - 5	Acceptable - 3	Scoring Notes
	Follow-up or further questioning is completed to elicit detail and full understanding of support to identify barriers or facilitators	Little to no follow-up questioning to understand barriers or facilitators	
2. Re-enforce positive social support. This may include informational/planning support, emotional support /approval, or practical support (e.g. helping the patient to make time for exercising).	Physio uses reflective listening skills to summarise positive social support with respect to exercise and re-enforces	Physio simply states that any support is positive, without the use of any reflective listening	No positive support identified → score NA.<br>Social support not evaluated → score 0.
3. Problem solves negative social support.	Physio uses reflective listening skills to summarise key points regarding the negative social support.<br>ASK-TELL-DISCUSS to facilitate problem solving<br>Discuss essential to score a 5<br>A menu of options provided when using TELL<br>Re-enforce positive solutions provided by patient using reflective listening techniques	Physio ASKs patient for solutions with how to improve social support (not how social support can help exercise).<br>Physio TELLs a solution. No options provided. No option to discuss. .	Physio only tells solutions → max 2.<br>DISCUSS prompted with a closed question → max 4.<br>No negative support identified → score NA.<br>Social support not evaluated → score 0.
4. Provide equipment as required.	5 if equipment given or patient has alternatives at	-	No equipment required → score NA.

Interventional Techniques	Perfect - 5	Acceptable - 3	Scoring Notes
	home (e.g., something to use as a weight [bag etc])		
5. Direct to the friends and family leaflet.	Physio physically shows the patient the friends and family leaflet and explains its benefits<br>If a social support is present physio provides them with the leaflet and explains its benefits	Physio states there is a friends and family leaflet. Benefits not explained.	Supporter present but not shown leaflet → max 4.
**Set a plan to perform exercise (PO1)** - Physiotherapist assists patient with planning exercise (i.e., dosage and action plans) *(Max score)*<br>***Notes:*** *Score NA if no new or modified exercises; NA if review identifies no issues.*			
1. Make an exercise dosage plan including frequency, reps, sets, position, and intensity of effort.	Dosage clearly articulated and clearly understood from audio-recordings<br>Minimum of frequency, reps, sets described.	Dosage given but may be unclear or provided in a fragmented way.<br>Only 2/3 of frequency, reps, sets are described	Only one dosage element provided → max 2.<br>Dosage written but not fully discussed → max 3.<br>Modification/progression of existing exercises only → score NA.
2. Co-create an exercise action plan with the patient, including where, when, and how social support will be engaged (either verbally or written).	ASK-TELL-DISCUSS used to outline where, when and how social support will be used<br>Discuss is essential for a 5.	Physio states, where, when and how social support will be used<br>No ASK or DISCUSS	Where/when/social support not all covered → max 2–3 (depending on detail).<br>Patient suggests only a single item with little physio reinforcement → score 1.

Interventional Techniques	Perfect - 5	Acceptable - 3	Scoring Notes
3. Evaluate self-efficacy with completing exercise dosage and action plans.	Open questioning use to evaluate confidence in completing the exercise programme and plan/Uses a 0-10 scale	Uses a closed question to evaluated confidence	Only brief closed-question check ("is that ok?") → max 2.<br>Confidence not asked explicitly → max 2.
4. Discuss potential barriers and facilitators (can be completed at any appointment).	Use physios open questioning to identify any potential barriers to completing exercise.<br>Further detail and questioning used to understand barrier/facilitators.<br>Reflective listening techniques used to summarise and highlight key barriers<br>If problems identified, problem solving scored elsewhere (Problem solving).	Physio uses closed questioning to identify any potential barriers/facilitators.<br>Reflective listening techniques used but maybe weak<br>If problems identified, problem solving scored elsewhere (Problem solving)	Pre-emptive exploration of barriers/facilitators only; actual barriers after implementation in "Reviewing progress & Problem solving"<br>Closed-question exploration → max 3.<br>Patient offers barrier/facilitator but not acknowledged → max 2.
5. Direct to workbook to record exercise dosage goals and exercise action plan.	Physio physically shows the patient where to record their exercise programme and dosage plan (can be exercise diary) AND action plan.	Physio states there is space for them in the resources to record their exercise dosage and action plan. Action plan essential for a 3.	Score recording of completion in "Self-monitoring"
**Encourage self-monitoring (PO1)** - Physiotherapist encourages patient to self-monitor exercise *(Max score)*<br>***Notes:*** *Diary review scored under "Review progress".*			

**Interventional Techniques**	**Perfect - 5**	**Acceptable - 3**	**Scoring Notes**
1. Discuss the benefits of self-monitoring exercise completion, dosage, and symptoms (gives feedback on progress, provides a basis for discussion with the PT at the next appointment).	Physio uses ASK-TELL-DISCUSS to explore the benefits of self-monitoring<br>Explicitly links to goals or exercise objectives/functional limitations	Physio explicitly states the benefits of self-monitoring. Needs to be more than "it's a good idea".	
2. Direct to workbook (exercise diary/wall chart/app) for self-monitoring.	Physio physically shows the patient where to record their exercise completion and explains how to use it.	Physio states there is space for them in the resources to record their exercise completion	"There is space to record" only (no physical direction) → max 3.<br>Encouraging use (but no physical showing) → max 4.
**Review progress** - Physiotherapist reviews progress *(Mean score)*<br>***Notes:*** *Scored as NA for initial appointment; Understanding progress only → actual barriers/facilitators are scored under Support problem solving.*			
1. Review of short and long-term neck pain outcomes (the initial outcome goals set and any other benefits or harms noticed by the patient).	Open questioning to elicit changes in patient symptoms and goals.<br>Detail/follow-up questions to explore/understand changes (or harms)<br>Reflective listening used to highlight key changes	Closed questioning used to elicit changes<br>Weak or limited follow-up to gain understanding of changes/progress<br>No reflective listening	
2. Review of performance objectives and exercise plans.	Open questioning to elicit progress with exercise and other performance objectives (once introduced).	Closed questioning to elicit progress/compliance with exercise and other performance objectives (once introduced).	PO must be evaluated at the next session after it was introduced → needed for score 3.

Interventional Techniques	Perfect - 5	Acceptable - 3	Scoring Notes
	Detail/follow-up questions asked to understand detail behind how exercise or performance objectives are being performed/engaged with/understand exercise.<br>Reflective listening used to highlight key changes and any potential barriers/positives stuff	Short or minimal questioning to understand detail behind how exercise or performance objectives are being performed/engaged with/understand exercise.<br>No reflective listening	
3. Review motivation (importance, safety, self-efficacy and social support)	Open questioning to review understanding of benefits and safety of performance objectives/exercise<br>Open questioning (or a 0-10 scale) to review confidence in completing performance objectives/exercise<br>Open questioning to review completion of action plan and social support<br>Reflective listening to summarise and highlight any key issues/re-enforce positive changes	Close questioning used and only 2/3 explored out of<br>1. Benefits/safety<br>2. Confidence<br>3. Action plan/social support<br>Limited reflective listening	
4. Provide feedback on progress with neck pain outcome goals and performance objectives.	Uses a formal summary to recap.<br>Progress with symptoms/goals,	Absence of a formal summary<br>Physio states someone is doing well or not doing well	

Interventional Techniques	Perfect - 5	Acceptable - 3	Scoring Notes
	POs/exercise and motivation factors all mentioned.<br>Strong reflective listening that highlights and emphasises key findings (both positive and negative)	(symptoms/goals AND exercise/POs).<br>Misses feedback on motivational factors<br>Weak reflective listening with weak highlighting of key findings (both positive and negative)	
**Support problem solving** - Physiotherapist supports problem solving throughout intervention delivery. Most commonly used when reviewing progress *(Mean score)*<br>***Notes:*** *Initial appointment = NA unless barriers raised; NA if "Review Progress" item 2 scores ≥3 and no issues reported with exercise*			
1. Discuss barriers to exercise or performance objective completion.	If when reviewing progress issues with exercise/PO identified, barriers to completion explored using open questioning.<br>Good follow-up questioning to gain depth to understanding of all barriers<br>Strong reflective listening skills	If when reviewing progress issues with exercise/PO identified, barriers to completion explored using closed questioning.<br>Limited depth to understanding barrier(s). May only explore one barrier.<br>No reflective listening	If issues with PO/exercise plan are unknown due to Reviewing Progress Point 2 → max 3.<br>Patient mentions barrier but physio does not follow up → max 2.
2. Facilitate problem solving.	Uses ASK-TELL-DISCUSS to facilitate problem solving<br>Discuss essential to score a 5<br>A menu of options provided when using TELL	Physio ASKs patient for solutions.<br>Physio TELLs a solution. No options provided. No option to discuss.	Problem-solving relating to flare-ups → score in PO3.<br>Physio stating a single solution and patient challenges/declines → max 1<br>DISCUSS prompted with closed question → max 4.

Interventional Techniques	Perfect - 5	Acceptable - 3	Scoring Notes
	Re-enforce positive solutions provided by patient using reflective listening techniques		
3. Direct to FAQ of the workbook.	Physio physically shows the patient the FAQ sections and used to facilitate discussion.	Physio states there is an FAQ section and states it is to support problem solving.	
**Introduce adapting and progressing (or regression) exercise (PO2)** - Physiotherapist discusses adapting and progressing exercise *(Max score)*			
1. Discuss the bio, psycho, and social (relationships & social activities) benefits and safety of adapting and progressing (or regressing) exercise.	Physio uses ASK-TELL-DISCUSS to explore that patient's perception of bio, psycho and social benefits of progressing or regressing exercise. Discuss after telling is essential to score a 5.<br>Physio links benefits back to key findings from history or patient goals<br>Use of ASK-TELL-Discuss to explore safety of exercise.<br>Discuss is essential to score a 5.	Physio states the bio, psycho and social benefits of progress or regressing exercise. All 3 essential to score a 3.<br>Physio states the exercises are safe to complete. No use of ASK-TELL-DISCUSS	Only biological benefits discussed → max 1.<br>Two of three domains discussed → max 2.
2. Instruct and demonstrate options of how to adapt and progress (or regress) exercise.	Verbal instructions must be clear enough to be understood the exercise via audio-recording.<br>Demonstrates any changes that reasonably require a	Instructions for progression only.<br>Verbal instruction provided, may still be unclear from audio-recording.	Verbal instruction unclear (from audio) → score 3.<br>Demonstration not required when only reps/weight change (already loaded).

Interventional Techniques	Perfect - 5	Acceptable - 3	Scoring Notes
	demonstration (e.g., changing position, or adding a weight to an unweighted exercise)	Demonstration not provided when reasonably could have been (e.g., no weight to dumbbell)	
3. Co-create a tailored plan to patients' ability and goals, outlining how and when to adapt and/or progress exercise, and how they can use social support for support (either verbally or written).	Plan clearly linked to ability and goals<br>Physio provides options of how to progress/regress exercise and patient choose which they prefer.<br>Physio articulates when they need to adapt exercise (use of achievable challenge acceptable but not essential).<br>ASK-TELL-DISCUSS used to explore how social support can be used	Plans unclear how linked to ability or goals<br>Physio states how to progress or regress exercise (no patient choice)<br>Physio articulates when they need to adapt exercise (use of achievable challenge acceptable but not essential).<br>Social support not discussed	"How" here is the co-creation of a plan using options to try and deciding. The instructions of how are scored above.<br>Same plan described for only one exercise without generalisation → max 3.
4. Discuss potential barriers (including self-efficacy) and facilitators to adapting or progressing exercise (can be completed at any stage).	Use physios open questioning to identify any potential barriers to progressing/regressing exercise.<br>Further detail and questioning used to understand barrier/facilitators.<br>Reflective listening techniques used to	Physio uses closed questioning to identify any potential barriers/facilitators.<br>No follow-up questioning<br>Reflective listening techniques used to summarise and highlight key barriers<br>If problems identified, problem solving scored elsewhere (Problem solving)	Pre-emptive exploration of barriers/facilitators only; actual barriers after implementation in "Reviewing progress & Problem solving"

Interventional Techniques	Perfect - 5	Acceptable - 3	Scoring Notes
	summarise and highlight key barriers<br>If problems identified, problem solving scored elsewhere (Problem solving).		
5. Practice adapting and/or progressing exercise in clinic and provide feedback.	Patient practices the modifications to progress/ regression (includes repetition changes)<br>Detailed feedback provided either using either positive re-enforcement if done well, or to correct technique	Patient practices any modifications.<br>Feedback is brief (e.g., well done, good)	Only brief/non-specific feedback (“good”, “well done”) → max 3.
6. Direct to Module 3 of the workbook for more detail.	Physio physically shows the patient the relevant module and uses it as a guide to discuss the performance objective.	Physio states there is a section in the workbook on the performance objective.	
**Introduce flare-up exercise (PO3)** - Physiotherapist discuss flare-ups and how they can be self-managed with exercise *(Max score)*			
1. Explore the benefits and safety of self-managing a flare-up with exercise.	Physio links benefits back to key findings from history or patient goals<br>Physio uses ASK-TELL-DISCUSS to explore that patient’s perception of the benefits of managing a flare-up with exercise. Discuss	Physio states any benefits of managing a flare-up with exercise.<br>Physio states that managing a flare-up with exercise independently is safe to complete. No use of ASK-TELL-DISCUSS	

Interventional Techniques	Perfect - 5	Acceptable - 3	Scoring Notes
	after telling is essential to score a 5.<br>Use of ASK-TELL-Discuss to explore safety managing a flare-up independently with exercise. Discuss is essential to score a 5.		
2. Co-create a flare-up exercise plan including which exercises and when to seek healthcare professional support. Plan how to use social support (either verbally or written).	Uses open questioning to prompt problem solving for anticipated flare-ups.<br>Physio provides the options of 1. Regress 2. Flare-up programme 3. Restart old exercises (any other options do not score a 5)<br>Use of ASK-TELL-DISCUSS to explore social support. Discuss essential for a 5.<br>Physio states when to seek HCP support.	Physio states/tells a single option for flare-up exercise. May or may not be the 3 options in the workbook/to score a 3.<br>Physio states/tells how to engage social support	No non-HCP social support discussion → max 2.
3. Use any feedback when reviewing progress to reinforce self-efficacy (if a flare-up plan was implemented) or to problem-solve (if something didn't go according to plan).	Open questioning to explore how exercise was used the manage the flare-up<br>Use of reflective listening to highlight key barriers, or issues/Use of reflective listening to highlight what was done well	Closed questioning reliance to explore how exercise was used<br>No summaries or reflective listening.<br>Physio states how to problem-solve exercise for flare-ups	No flare-up between appointments → score NA.<br>Only non-exercise modalities discussed → max 2.<br>Reviewing progress/problem solving here is related to the success of managing a flare-up (outcome). Any barriers/facilitators to

Interventional Techniques	Perfect - 5	Acceptable - 3	Scoring Notes
	Re-enforce and praise any positive actions.<br>Use of ASK-TELL-DISCUSS to problem solve how exercise was implemented for the flare-ups		implementation of the exercise scored under Reviewing Progress/Problem Solving.
4. Direct to the Module 4 of workbook for more detail.	Physio physically shows the patient the relevant module and uses it as a guide to discuss the performance objective.	Physio states there is a section in the workbook on the performance objective.	
**Introduce general exercise +/- secondary prevention exercise (PO4)** - Physiotherapist encourages general exercises and discusses concept of secondary prevention exercise *(Max score)*			
1. Discuss bio, psycho, and social (relationships & social activities) benefits of secondary prevention and general exercise.	Physio states the pros and cons of secondary prevention exercise<br>ASK-TELL-DISCUSS to elicit views of bio, psycho and social benefits of general exercise. Discuss and all 3 domains essential for a 5.	Physio states the pros and cons of secondary prevention exercise<br>Physio states the bio, psycho and social benefits of general exercise.	Two of three domains discussed → max 2.
2. Set long-term general exercise goals.	Goals set that are measurable.<br>Can be behavioural (exercise 5 times per week) or outcome (lose weight)	Goals set that are not measurable<br>Can be behavioural (exercise 5 times per week) or outcome (lose weight)	

Interventional Techniques	Perfect - 5	Acceptable - 3	Scoring Notes
3. Set a general exercise +/- secondary prevention exercise plan.	Collaborative decision between patient and physio as to whether to engage with secondary prevention exercise<br>ASK-TELL-DISCUSS to set an action plan of what, where, when and who can support general exercise. Discuss essential for a 5.<br>ASK-TELL-DISCUSS how to integrate secondary prevention exercise if opting in. Discuss essential for a 5.	Collaborative decision between patient and physio as to whether to engage with secondary prevention exercise<br>ASK-TELL to set an action plan of what and who can support general exercise (where and when not essential for a 3)<br>Physio states how to integrate secondary prevention exercise if opting in.	
4. Encourage self-monitoring.	Physio uses ASK-TELL-DISCUSS to explore the benefits of self-monitoring	Physio explicitly states they should self-monitoring general exercise	
5. Direct to Module 5 of the workbook for more detail.	Physio physically shows the patient the relevant module and uses it as a guide to discuss the performance objective.	Physio states there is a section in the workbook on the performance objective.	
**Evaluate self-management and discharge suitability** - Physiotherapists evaluates whether a patient is able to self-manage and therefore suitable for discharge *(Max score)*			
1. Initiate conversations regarding discharge.	Physio asks patient opinion on discharge using open questions.	Physio states they are ready for discharge	

Interventional Techniques	Perfect - 5	Acceptable - 3	Scoring Notes
2. Evaluate and determine adequate motivation (importance, safety, self-efficacy and social support) in using performance objectives.	Physio uses open questioning to confirm patient understand the benefits of all 4 performance objectives<br>Physio uses open questioning to confirm patient has adequate social support for exercise (not self-management of neck pain)<br>Physio uses open questioning to confirm self-efficacy with completing all 4 performance objectives	Closed questioning to confirm patient understands the benefits all 4 performance objectives<br>Closed questioning to confirm patient has adequate social support for exercise (not self-management of neck pain)<br>Closed questioning to confirm self-efficacy with all 4 performance objectives.	
3. Evaluate and confirm progress towards short and long-term neck pain outcome goals.	Physio explicitly asks patient to re-evaluate and confirm their progress towards their goals	Physio states patient is progressing towards goals (patient not asked)	No goals set but symptom changes used → max 3
4. Evaluate patient's ability to review progress and problem solve independently post-discharge.	Physio uses open questions to hypothesis what patient would do if any exercise related problems occurred post-discharge<br>Patient answers appropriately.	Physio states the patient is able to problem solve independently	Specifically related to exercise, and use of exercise to manage symptoms.<br>Closed-question check ("can your problem-solve?") → max 4.
**<u>Intervention components to maximise effectiveness</u>**			
**Identify short-term outcomes and exercise objectives (PO1)** - Use of history taking and physical examination to identify appropriate short-term outcomes and specific exercise objectives *(Max score)*			

Interventional Techniques	Perfect - 5	Acceptable - 3	Scoring Notes
1. Explore each potential short-term outcome (pain, disability/functional limitations, sleep, mental well-being and relationship impact) in history taking.	All domains explored with use of open questioning<br>Reflective listening used to highlight key findings within each domain<br>Summary used at the end of history to reflect each of the domains, and to highlight which domain(s) are a priority.	All domains explored with reliance on closed questioning	
2. Explore exercise objectives (fear/catastrophising and pain self-efficacy) for pain and disability.	All objectives explored with use of open questioning<br>Reflective listening used to highlight key findings within each objective<br>Summary used at the end of history to reflect each of the objective(s), and to highlight which if any are a priority.	All objectives explored with reliance on closed questioning	
3. Use physical examination techniques where required to identify and define exercise objective.	Clear and appropriate use of cervical, shoulder, scapular neuromuscular function examination articulating key findings to patient<br>Clear and appropriate use of graded exposure or self-efficacy examination where required	Basic neuromuscular function examination. May miss some areas or components<br>Brief use of graded exposure or self-efficacy examination where required	Neuro/myotomal testing only → max 2.<br>No cervical neuromuscular function evaluation → max 4.

Interventional Techniques	Perfect - 5	Acceptable - 3	Scoring Notes
**Home exercise programme – Normalising movement (PO1)** - Home exercise programme prescribed *(Max score)*<br>***Notes:*** *Score NA on follow-up if previously prescribed and no new exercises.*			
1. Exercise programme utilises active exercise for the neck or surrounding regions aiming to restore movement that maybe dysfunctional due to any reason (if appropriate)	Appropriate exercises prescribed<br>Purpose of exercises clearly articulated as restoring normal movement	Largely appropriate exercises prescribed<br>Purpose of exercises not articulated to patient	Passive stretches relied on → max 2.
2. Exercises tailored to goal, examination findings and exercise objectives	Exercises matched to goals<br>Exercises clearly and appropriately matched to examination findings<br>Exercises tailored to appropriate exercise objectives<br>Physio explicitly states how they are linked to goals and examination findings	Exercises matched to examination findings but not necessarily tailored to goals or exercise objectives.<br>Link between exercises and examination findings may not be explicitly clear or expert	No goals set → max 4.
3. Exercise and dosage prescribed collaboratively to be an "achievable challenge".	Exercise dosage clearly set by physiotherapists<br>Exercise dosage reasonable to affect the desired target<br>Open questioning used to determine acceptability to patient<br>Benchmarked against achievable challenge	Exercise dosage set but potentially unclear<br>Exercise dosage may not be appropriate/effective for desired target<br>Some attempts to benchmark against "achievable challenge" or patients' effort.	Fully self-directed ("achievable challenge" only) and not contextualised as fear/catastrophising → max 2.
**Home exercise programme – Improving tolerance (PO1)** - Home exercise programme prescribed *(Max score)*			

Interventional Techniques	Perfect - 5	Acceptable - 3	Scoring Notes
Notes: *Score NA on follow-up if previously prescribed and no new exercises.*			
1. Exercise programme utilises active exercises for the neck or surrounding regions aiming to progressively improve the tolerance of the neck to cope with increasing demands that are not just physical (if appropriate)	Appropriate exercises prescribed<br>Purpose of exercises clearly articulated as improving tolerance	Largely appropriate exercises prescribed<br>Purpose of exercises not articulated to patient	
2. Exercises tailored to goal, examination findings and exercise objectives	Exercises matched to goals<br>Exercises clearly and appropriately matched to examination findings<br>Exercises tailored to appropriate exercise objectives<br>Physio explicitly states how they are linked to goals and examination findings	Exercises matched to examination findings but not necessarily tailored to goals or exercise objectives.<br>Link between exercises and examination findings may not be explicitly clear or expert	No goals set → max 4.
3. Exercise and dosage prescribed collaboratively to be an “achievable challenge”.	Exercise dosage clearly set by physiotherapists and reasonable to affect the desired target<br>Open questioning used to determine acceptability to patient	Exercise dosage set but potentially unclear or may not be appropriate/effective for desired target<br>Some attempts to benchmark against “achievable challenge” or patients’ effort.	Fully self-directed dosage (without physio guidance) → max 2.

**Interventional Techniques**	**Perfect - 5**	**Acceptable - 3**	**Scoring Notes**
	Benchmarked against achievable challenge		
**Exercise Review** (PO1) - At follow-up appointments exercises reviewed for appropriateness and modified, progressed or regressed as needed *(Mean score)*<br>***Notes:*** *Initial appointment = NA.*			
1. At follow-up appointments exercises reviewed for continued appropriateness, and modified, progression/regressed as needed	Physio evaluates appropriateness of exercise verbally<br>Patients instructed to demonstrate all exercises<br>Repeated or further physical examination used to confirm suitability of exercises.<br>Appropriate modifications made based on demonstration, subjective and physical exam findings. If no changes must state, no changes.	Physio evaluates appropriateness of exercises verbally only<br>Modification made based on history/subjective findings only. If not, changes must state, no changes.	Appropriateness not asked (verbally or via demonstration) → max 2.<br>At discharge: no review but patient states exercise OK → max 4.<br>Significant new exercise added at discharge but not practiced → max 1. Technique modification at discharge → score 5.

68 Footnote: NA scored when component or intervention not eligible for scoring

Table S4 - EPIC-Neck component delivery fidelity explanations

What was done well from appointment audio-recordings	What was done poorly from appointment audio-recordings	Interview derived barriers	Interview derived facilitators
**Agree outcome goals: 2.0 (1.3)**			
Regularly asked patients whether they had goals.	Goals rarely co-developed using ASK–TELL–DISCUSS.<br>Goals rarely measurable.<br>Goals not explicitly agreed or time-bound.	**Intervention factors:** Low priority; theory–practice mismatch.<br>**Patient factors:** Low engagement; limited understanding; perceived preferences/expectations; unfamiliar concepts.<br>**Physiotherapist capability:** Low confidence; low skill; overestimated fidelity.<br>**Practice integration:** Extra effort required; superficial delivery; time-consuming.	NA
**Actively involve the patient: 3.1 (0.7)**			
Open questioning used occasionally.<br>Shared decision making attempted regularly.	ASK–TELL–DISCUSS rarely applied.<br>Reflective listening used inconsistently.	**Patient factors:** Communication or language barriers; low engagement; perceived preferences.<br>**Physiotherapist capability:** Forgetfulness; low confidence with difficult conversations/silence.<br>**Practice integration:** More detailed than routine practice; time-consuming.	**Physiotherapist capability:** Training quality<br>**Practice integration:** Similar to routine practice.
**Improve motivation to perform exercise (PO1) - Enhance perceived importance and safety: 2.1 (1.0)**			

What was done well from appointment audio-recordings	What was done poorly from appointment audio-recordings	Interview derived barriers	Interview derived facilitators
Biological benefits of exercise regularly stated.	Psychological and social benefits rarely discussed.<br>Exercise safety rarely communicated.<br>ASK–TELL–DISCUSS rarely used for safety/benefits.	No interview derived explanations	NA
**Improve motivation to perform exercise (PO1) - Improve self-efficacy: 3.5 (0.8)**			
Verbal instructions consistently provided.<br>Patients regularly practised exercises in clinic.<br>Exercise difficulty regularly tailored.<br>Supporting resources regularly used.	Exercise demonstrations inconsistent.<br>Feedback after practice brief or absent.	**Practice integration:** Not routine practice (demonstrations).	**Practice integration:** Integrated into routine practice; similar to routine practice.
**Improve motivation to perform exercise (PO1) - Engage social support: 1.8 (1.1)**			
Friends and family leaflet consistently provided.<br>Equipment provided when needed.	Social support rarely discussed.<br>Positive social support rarely reinforced.<br>Negative social support rarely problem-solved.	**Intervention factors:** Low priority; selective use based on presentation.<br>**Patient factors:** Low engagement; low perceived relevance/value; social support; perceived comfort.<br>**Physiotherapist capability:** Forgetfulness; low confidence; low skill.	NA

What was done well from appointment audio-recordings	What was done poorly from appointment audio-recordings	Interview derived barriers	Interview derived facilitators
		**Practice integration:** Not routine practice; superficial delivery to reduce effort.<br>**Resources:** Perceived sufficiency of leaflets without discussion.	
**Set a plan to perform exercise (PO1): 2.3 (1.0)**			
Exercise dosage regularly described.	Action plans discussed irregularly.<br>Social support rarely included in action plans.<br>ASK–TELL–DISCUSS rarely used for action planning.<br>Self-efficacy and barriers rarely evaluated.	**Intervention factors:** Selective use based on presentation.<br>**Practice integration:** Not routine practice (action plans).	NA
**Encourage self-monitoring (PO1): 3.3 (0.9)**			
Benefits of self-monitoring regularly stated.<br>Exercise diary regularly explained.	ASK–TELL–DISCUSS rarely used for self-monitoring discussions.	No interview derived explanations	**Resources:** Prompts and structure
**Review progress: 2.7 (0.5)**			
Open questions with follow-up used consistently to elicit symptom change.	PO1 exercise completion rarely explored in depth.<br>Performance objectives 2–4 rarely explored.<br>Motivation factors rarely revisited.	No interview derived explanations	NA

What was done well from appointment audio-recordings	What was done poorly from appointment audio-recordings	Interview derived barriers	Interview derived facilitators
	Reflective listening used sparingly.		
**Support problem solving: 1.3 (0.7)**			
No consistent techniques observed.	Problems inconsistently explored in depth.<br>ASK–TELL–DISCUSS rarely used for problem solving.<br>Problem-solving resources not used.	**Organisational factors:** Pressure to discharge; service design.<br>**Patient factors:** Perceived preferences; perceived comfort.<br>**Physiotherapist capability:** Forgetfulness; low confidence (silence); overestimated fidelity.<br>**Practice integration:** Hard to change habits; not routine practice; extra effort required; superficial delivery.	NA
**Introduce adapting and progressing (or regression) exercise (PO2): 1.6 (1.1)**			
How and when to progress exercise regularly stated.<br>Patients regularly directed to the workbook chapter.	Regression rarely discussed.<br>ASK–TELL–DISCUSS rarely used for progression/regression decisions.<br>Bio-psycho-social benefits of progression/regression not discussed.<br>Social support for progression/regression not discussed.	No interview derived explanations	NA

What was done well from appointment audio-recordings	What was done poorly from appointment audio-recordings	Interview derived barriers	Interview derived facilitators
	Self-efficacy and barriers in progression/regression not discussed.		
**Introduce flare-up exercise (PO3): 1.5 (1.0)**			
Flare-up strategies regularly stated.<br>Patients regularly directed to workbook chapter	ASK–TELL–DISCUSS rarely used for flare-up plans.<br>Benefits and safety of self-managing flare-ups rarely discussed.<br>Non-HCP social support not discussed.<br>HCP support rarely discussed.<br>Feedback/problem-solving after flare-ups absent.	**Intervention factors:** Selective use based on presentation.<br>**Physiotherapist capability:** Overestimated delivery fidelity.	NA
**Introduce general exercise +/- secondary prevention exercise (PO4): 0.9 (0.9)**			
Patients occasionally directed to workbook chapter.	Benefits and safety of general exercise rarely discussed.<br>Secondary prevention pros/cons discussed irregularly.<br>General exercise goals and plans not discussed.	**Intervention factors:** Selective use based on presentation.<br>**Physiotherapist capability:** Overestimated delivery fidelity.<br>**Practice integration:** More detailed than routine practice.	NA
**Evaluate self-management and discharge suitability: 2.0 (1.2)**			

What was done well from appointment audio-recordings	What was done poorly from appointment audio-recordings	Interview derived barriers	Interview derived facilitators
Discharge suitability regularly evaluated using shared decision making.<br>Symptom changes regularly evaluated.	Goal progress rarely confirmed.<br>Motivation factors not confirmed.<br>Ability to problem-solve rarely confirmed.	**Intervention factors:** Theory–practice mismatch.<br>**Organisational factors:** Pressure to discharge; service design.<br>**Resources:** Inconsistent use.	NA
**Identify short-term outcomes and exercise objectives (PO1): 3.1 (0.8)**			
Pain, disability, sleep, and function regularly evaluated.<br>Scapulothoracic neuromuscular exams completed consistently.	Mental well-being and relationships rarely evaluated.<br>Fear/catastrophising and self-efficacy rarely evaluated.<br>Cervical neuromuscular exams limited and irregular.<br>Graded exposure exams largely absent.	**Intervention factors:** Low priority (non-pain/disability, motor control); selective use based on presentation.<br>**Patient factors:** Low engagement; perceived preferences; perceived expectations.<br>**Physiotherapist capability:** Forgetfulness; limited cognitive/emotional capacity; limited knowledge (fear, motor control); low confidence (non-pain/disability, fear, self-efficacy, motor control); low skill (non-pain/disability, motor control); overestimated fidelity; training quality (motor control).<br>**Practice integration:** More detailed than routine practice; not routine practice (non-pain/disability, motor control); reverting to easier routine practice (exercise objectives, fear).	**Intervention factors:** Intervention complexity (sleep, relationships).<br>**Physiotherapist capability:** Baseline skill (motor control); training quality (non-pain/disability, physical examination).<br>**Practice integration:** Integrated into routine practice; similar to routine practice.<br>**Resources:** Prompts and structure.

What was done well from appointment audio-recordings	What was done poorly from appointment audio-recordings	Interview derived barriers	Interview derived facilitators
**Home exercise programme – Normalising movement (PO1): 3.5 (0.7)**			
Normalising-movement exercises regularly prescribed.<br>Dosage consistently sufficient.<br>“Achievable challenge” occasionally used collaboratively.	Matching exercises to findings irregular.<br>Tailoring to objectives rare.<br>Exercise purpose and linkage rarely articulated.	**Intervention factors:** Low priority (non-pain/disability, tailoring).<br>**Patient factors:** Low engagement; perceived preferences.<br>**Physiotherapist capability:** Limited knowledge (relationships, motor control, tailoring); low confidence (non-pain/disability, fear, self-efficacy, motor control, tailoring); low skill (tailoring); overestimated fidelity; training quality (motor control).<br>**Practice integration:** Hard to change habits; not routine practice (motor control); superficial delivery to reduce effort.	**Intervention factors:** Intervention complexity (achievable challenge concept).<br>**Physiotherapist capability:** Training quality (achievable challenge concept).
**Home exercise programme – Improving tolerance (PO1): 2.7 (1.6)**			
Tolerance-building exercises occasionally prescribed.<br>Dosage sometimes sufficient.<br>“Achievable challenge” occasionally used collaboratively.	Matching exercises to findings irregular.<br>Tailoring to objectives rare.<br>Exercise purpose and linkage rarely articulated.	**Intervention factors:** Low priority (non-pain/disability, tailoring).<br>**Patient factors:** Low engagement; perceived preferences.<br>**Physiotherapist capability:** Limited knowledge (relationships, tailoring); low confidence (non-pain/disability, fear, self-efficacy, tailoring); low skill (tailoring); overestimated fidelity.	**Intervention factors:** Intervention complexity (achievable challenge concept).<br>**Physiotherapist capability:** Training quality (achievable challenge concept).

What was done well from appointment audio-recordings	What was done poorly from appointment audio-recordings	Interview derived barriers	Interview derived facilitators
		**Practice integration:** Hard to change habits; superficial delivery to reduce effort.	
**Exercise Review (PO1): 3.0 (1.2)**			
Exercises regularly reviewed verbally.<br>Physical examinations regularly completed to confirm suitability.<br>Exercises modified based on verbal review and physical examination.	Exercises rarely reviewed physically.<br>Exercises rarely removed if inappropriate.<br>Continuation of unchanged exercises rarely stated.	No interview derived explanations	No interview derived explanations

Scores <3 unacceptable delivery, 3+ acceptable delivery, 5 perfect delivery.

What was done well/poorly taken from audio-recordings delivery fidelity checklist

Interview extracts were used to identify facilitators and/or barriers to delivery. For items scored <3, only barriers were reported; for components scored 3–4, both facilitators and barriers were reported; and for components scored 5, only facilitators were reported. Where no relevant interview data were available, this is stated explicitly.

Table S5 – EPIC-Neck participant by component delivery fidelity scores

	001	002	004	005	007	008	010	011	012	015	017	018	019	020	022	023	025	028	029	030	031	032	033	034	**Mean**
**Discharge Outcome**	DNA	✓	✓	DNA	✓	✓	✓	✓	✓	✓	X	DNA	✓	✓	✓	✓	DNA	✓	✓	X	✓	✓	✓	✓	
**Total Attended Appointments**	1	3	5	4	3	5	2	5	7	4	0	1	5	4	5	4	3	4	3	0	4	3	3	2	**3.3**
**Total DNA**	1	0	1	2	1	0	1	0	1	0	0	1	0	1	0	1	3	0	1	1	0	1	0	0	**0.6**
**Total Appointments**	2	3	6	6	4	5	3	5	8	4	0	2	5	5	5	5	6	4	4	1	4	4	3	2	**4.0**
**Duration (weeks)**	3	14.6	22.6	29.9	16.6	16.1	9.6	18.4	29.4	14.7	-	10.6	20.1	19.6	24	22.1	36	9	24.3	-	10.6	7	8	6	**16.9**
**Physio**	PT3	PT2	PT4 PT3	PT2 PT3	PT4	PT3	PT4 PT3	PT3	PT7 PT1	PT1 PT3	-	PT4	PT2	PT7 PT1	PT1	PT1	PT2	PT5	PT2	-	PT6	PT6	PT3	PT2	
**Item**																									
**01**	2.0	4.0	1.0	2.0	0.0	3.0	0.0	1.0	2.0	3.0	-	0.0	2.0	5.0	3.0	2.0	0.0	2.0	3.0	-	2.0	2.0	3.0	3.0	**2.0**
**02**	3.5	2.5	3.0	2.8	2.5	4.1	3.5	3.8	2.4	4.1	-	2.0	2.1	3.5	2.9	2.2	2.5	3.5	2.5	-	3.2	4.0	4.0	2.8	**3.1**
**03**	4.0	1.0	3.0	2.0	1.0	2.0	3.5	2.5	3.5	3.5	-	1.0	1.0	2.0	1.0	1.5	1.0	2.5	1.0	-	2.5	2.5	2.5	1.0	**2.1**
**04**	4.0	3.0	3.5	5.0	2.5	3.7	2.0	3.5	4.0	4.0	-	1.0	3.0	4.0	4.0	3.3	4.0	3.7	3.0	-	4.0	4.0	3.3	4.0	**3.5**
**05**	1.0	1.0	1.0	2.0	2.0	4.0	0.0	3.0	2.0	1.0	-	1.0	1.0	4.0	3.0	3.0	1.0	1.0	2.0	-	2.0	2.0	2.0	1.0	**1.8**
**06**	2.0	3.0	1.0	3.0	1.0	4.0	1.0	2.0	2.0	4.5	-	1.0	3.0	2.0	2.0	3.0	3.0	3.0	1.0	-	2.0	2.0	2.0	3.0	**2.3**
**07**	3.5	3.0	3.5	4.0	2.5	4.5	2.5	3.0	4.0	4.0	-	3.0	4.0	3.0	4.0	3.0	3.0	4.0	4.0	-	3.5	3.5	0.0	4.0	**3.3**
**08**	-	2.0	2.6	2.5	2.8	3.0	4.0	2.4	2.5	3.7	-	-	2.5	2.5	2.8	2.7	2.0	2.8	2.2	-	2.7	-	3.0	3.0	**2.7**
**09**	-	1.0	2.0	1.2	1.2	1.3	3.0	1.0	1.0	1.7	-	-	1.0	0.7	1.0	2.0	1.0	2.5	1.0	-	0.0	-	1.5	-	**1.3**
**10**	2.0	2.0	3.0	1.0	1.0	3.0	0.0	3.0	3.0	3.0	-	1.0	2.0	3.0	1.0	2.0	0.0	0.0	1.0	-	1.0	0.0	2.0	2.0	**1.6**
**11**	0.0	1.0	3.0	1.0	1.0	3.0	0.0	2.0	2.0	3.0	-	1.0	2.0	1.0	2.0	3.0	2.0	0.0	2.0	-	2.0	0.0	1.0	2.0	**1.5**
**12**	0.0	1.0	1.0	2.0	1.0	0.0	0.0	1.0	0.0	1.0	-	0.0	1.0	2.0	2.0	3.0	0.0	0.0	1.0	-	2.0	0.0	0.0	1.0	**0.9**
**13**	0.0	1.0	3.0	0.0	2.0	2.0	5.0	2.5	2.0	2.5	-	0.0	2.0	2.0	3.0	3.0	0.0	2.5	2.0	-	2.0	-	3.0	2.0	**2.0**
**14**	3.0	3.0	5.0	2.0	4.0	2.0	4.0	3.0	4.0	4.0	-	3.0	2.0	4.0	3.0	3.0	2.0	3.0	3.0	-	4.0	3.0	3.0	2.0	**3.1**
**15**	3.0	2.0	4.0	3.0	4.0	4.0	3.0	4.0	4.0	4.0	-	2.0	3.0	4.0	3.0	4.0	3.0	4.0	4.0	-	4.0	4.0	4.0	4.0	**3.5**
**16**	0.0	3.0	4.0	0.0	3.0	4.0	0.0	5.0	4.0	4.0	-	2.0	3.0	4.0	4.0	4.0	0.0	3.0	3.0	-	3.0	0.0	4.0	3.0	**2.7**
**17**	-	2.0	3.5	1.0	1.5	4.8	1.0	3.8	3.0	3.0	-	-	3.0	3.7	4.0	4.0	1.0	5.0	2.5	-	3.0	-	3.5	3.0	**3.0**
**Mean**	2.0 (1.6)	2.1 (1.0)	2.8 (1.2)	2.0 (1.3)	1.9 (1.1)	3.1 (1.3)	1.9 (1.8)	2.7 (1.1)	2.7 (1.2)	3.2 (1.1)	-	1.3 (1.0)	2.2 (0.9)	3.0 (1.2)	2.7 (1.0)	2.9 (0.7)	1.5 (1.3)	2.5 (1.5)	2.2 (1.0)	-	2.5 (1.1)	2.1 (1.6)	2.5 (1.3)	2.6 (1.0)	2.0 (1.6)

Abbreviations: DNA – Did not attend; ✓ - Completed treatment; X - No treatment; PT – Physiotherapist; 01 - Agree outcome goals; 02 - Actively involve the patient; 03 - Improve motivation to perform exercise (PO1) - Enhance perceived importance and safety; 04 - Improve motivation to perform exercise (PO1) - Improve self-efficacy; 05 - Improve motivation to perform exercise (PO1) - Engage social support; 06 - Set a plan to perform exercise (PO1); 07 - Encourage self-monitoring (PO1); 08 - Review progress; 09 - Support problem solving; 10 - Introduce adapting and progressing (or regression) exercise (PO2); 11 - Introduce flare-up exercise (PO3); 12 - Introduce general exercise +/- secondary prevention exercise (PO4); 13 - Evaluate self-management and discharge suitability (PO1); 14 - Identify short-term outcomes and exercise objectives (PO1); 15 - Home exercise programme – Normalising movement (PO1); 16 - Home exercise programme – Improving tolerance (PO1); 17 - Exercise review (PO1)

Legend: Green (3.0–5.0) - Acceptable delivery; Amber (2.0–2.9) - Minor delivery concerns; Red (0.0–1.9) - Major delivery concerns; “-” No data

Table S6 – Usual exercise care participant by component delivery fidelity scores

	003	006	009	013	014	016	021	024	026	027	035	036	**Mean**
**Discharge Outcome**	X	✓	DNA	✓	X	✓	DNA	✓	DNA	X	✓	✓	
**Total Attended Appointments**	0	9	2	6	0	1	2	3	1	0	4	4	**2.7**
**Total DNA**	0	0	2	1	1	0	3	1	3	2	0	0	**1.1**
**Total Appointments**	0	9	4	7	1	1	5	4	4	2	4	4	**3.8**
**Duration (weeks)**	-	14.3	11.4	28.9	-	0	15	25	19	4	9	10.3	**13.7**
**Physio**	-	PT8	PT9	PT10	-	PT10	PT11	PT12	PT13	-	PT14	PT15	
**Item**													
**01**	-	0.0	1.0	1.0	-	0.0	0.0	1.0	1.0	-	1.0	3.0	0.9
**02**	-	1.4	1.0	1.3	-	1.0	1.0	1.2	1.0	-	1.0	2.2	1.2
**03**	-	1.5	1.0	2.5	-	1.0	1.0	1.0	1.0	-	2.0	2.0	1.4
**04**	-	2.0	4.0	2.5	-	1.0	2.5	3.5	3.0	-	4.0	4.5	3.0
**05**	-	0.0	0.0	0.0	-	0.0	1.0	1.0	1.0	-	0.0	1.0	0.4
**06**	-	1.0	1.0	1.0	-	1.0	1.0	1.0	1.0	-	1.0	1.0	1.0
**07**	-	0.0	0.0	0.0	-	0.0	0.0	0.0	0.0	-	0.0	0.0	0.0
**08**	-	1.4	2.0	1.5	-	-	2.0	1.5	-	-	1.5	1.2	1.6
**09**	-	0.2	1.0	0.8	-	-	1.0	1.0	-	-	1.3	0.0	0.8
**10**	-	0.0	0.0	1.0	-	0.0	1.0	0.0	0.0	-	0.0	0.0	0.2
**11**	-	0.0	0.0	0.0	-	0.0	0.0	0.0	0.0	-	0.0	1.0	0.1
**12**	-	0.0	0.0	1.0	-	0.0	0.0	0.0	0.0	-	0.0	0.0	0.1
**13**	-	1.5	0.0	2.0	-	0.0	0.0	0.0	0.0	-	1.5	2.0	0.8
**14**	-	1.0	1.0	1.0	-	1.0	1.0	1.0	1.0	-	1.0	1.0	1.0
**15**	-	3.0	2.0	3.0	-	0.0	3.0	1.0	2.0	-	2.0	2.0	2.0
**16**	-	1.0	2.0	3.0	-	3.0	3.0	2.0	2.0	-	2.0	2.0	2.2
**17**	-	1.1	2.0	2.0	-	-	1.0	4.0	-	-	3.0	2.0	2.2
**Mean**	-	0.9 (0.9)	1.1 (1.1)	1.4 (1.0)	-	0.6 (0.9)	1.1 (1.0)	1.1 (1.2)	0.9 (0.9)	-	1.3 (1.1)	1.5 (1.2)	1.1

Abbreviations: DNA – Did not attend; ✓ - Completed treatment; X - No treatment; PT – Physiotherapist; 01 - Agree outcome goals; 02 - Actively involve the patient; 03 - Improve motivation to perform exercise (PO1) - Enhance perceived importance and safety; 04 - Improve motivation to perform exercise (PO1) - Improve self-efficacy; 05 - Improve motivation to perform exercise (PO1) - Engage social support; 06 - Set a plan to perform exercise (PO1); 07 - Encourage self-monitoring (PO1); 08 - Review progress; 09 - Support problem solving; 10 - Introduce adapting and progressing (or regression) exercise (PO2); 11 - Introduce flare-up exercise (PO3); 12 - Introduce general exercise +/- secondary prevention exercise (PO4); 13 - Evaluate self-management and discharge suitability (PO1); 14 - Identify short-term outcomes and exercise objectives (PO1); 15 - Home exercise programme – Normalising movement (PO1); 16 - Home exercise programme – Improving tolerance (PO1); 17 - Exercise review (PO1)

Legend: Green (3.0–5.0) – Acceptable delivery of EPIC-Neck component/significant presence of EPIC-Neck; Amber (2.0–2.9) - Minor delivery concerns/some presence of EPIC-Neck; Red (0.0–1.9) - Major delivery concerns/no presence of EPIC-Neck; “-” No data

Data S3 - Delivery fidelity interview themes and illustrative quotes

Theme 1 - Alignment with routine practice shaped the ease of delivery (Practice integration)

Practice integration acted as a barrier and facilitator to delivery fidelity across nine and three checklist components respectively. Physiotherapists found delivery challenging when it was new (social support, facilitated problem-solving, action planning) or required more depth than routine practice (e.g., goal setting, non-pain/disability evaluation and motor control assessment/prescription). These components required disrupting established habits, requiring additional effort and time.

> *"I'm a relatively experienced clinician and I have patterns of how I do things and then this meant having to reframe some of that to make it fit, which almost threw me off" PT5 - 6-10 years' experience*

> *"I remember it was hard. Like when you learn to drive and you have to think about each step. But it's not quite like that because it's doing something which you did quite automatically without having to think too much and then making it something that you have to kind of consciously think about a lot. So that that was initially pretty tricky." PT1 - 6-10 years' experience*

Subsequently some physiotherapists skipped delivering components, reverted to easier established routines or delivered components superficially to reduce cognitive or practical burden. Conversely, components that aligned with, or were subsequently integrated into routine practice (demonstrating/practicing exercises, some history-taking elements, communication techniques) were delivered more consistently and with less effort.

> *Regarding communication techniques "I think for me that was something that was more integrated in my practise anyway, so it wasn't that hard." PT1 - 6-10 years' experience*

Overall fidelity was supported when the EPIC-Neck programme mirrored routine practice but was more challenging when it required deviation from established practices.

Theme 2 – Both recognised and unrecognised capability gaps shaped delivery quality (Physiotherapist capability)

Physiotherapist capability acted as both a barrier and facilitator to delivery across eight and three checklist components respectively. Limited knowledge, skills and confidence were all highlight as restricting delivery for elements such as goal setting, fear and non-pain/disability evaluation, social support engagement, facilitated problem solving, difficult conversations and motor control assessment.

> *"Getting the patient to set their own goals…I found that a difficult conversation to initiate" PT2 - 11-20 years' experience*

> *Regarding motor control examination "I wasn't really confident...it felt strange to me when I learned how to do it and then I never really got fully confident…maybe I just wasn't super confident in knowing or interpreting the results" PT3 - 0-2 years' experience*

Forgetfulness and reduced cognitive or emotional capacity during sessions further affected consistent delivery. Physiotherapists attributed the reduce capacity to training gaps (especially around motor control), limited opportunities for practice, and the absence of feedback. In contrast, physiotherapists felt the training had strengthened their capabilities particularly in communication skills and physical examination techniques. However, this perceived capability did not always match quantitative observations, with physiotherapists frequently overestimating how fully components (e.g., flare-ups, general exercise) had been delivered.

> *Regarding PO3/4 which was consistently scored low via audio-recording analysis "the whole journey of understanding flare ups, self-management, moving on to general exercise wasn't far away from what I did before so I was able to help the patient frame the journey." PT1 - 6-10 years' experience*

Overall, physiotherapists capability supported fidelity when adequate knowledge, skill and confidence were present but created barriers where it was insufficient or where unrecognised gaps persisted.

Theme 3 – Comprehensiveness and perceived relevance influenced selective delivery in practice (Intervention factors)

Intervention-related factors acted as both barriers and facilitators to fidelity across eight and two checklist components respectively. Physiotherapists described the comprehensiveness of the EPIC-Neck programme as positive but noted that its breadth made consistent delivery challenging.

> *"I feel like the content is very appropriate…but it's almost so comprehensive that you can get lost in it" PT5 - 6-10 years' experience*

Several components were assigned low priority or perceived as having limited clinical value, leading to selective delivery. At times this selectivity reflected patient characteristics or assessment findings (e.g., social support assessment, non-pain/disability evaluation), while at other times it reflected physiotherapists' own beliefs about the usefulness or relevance of particular components (e.g., goal setting, non-pain/disability evaluation).

> *"Some of the more in-depth questioning I will pick and choose who I do that with, based on what they've tried before in the symptom that they've got" PT3 - 0-2 years' experience*

Physiotherapists also highlighted tensions between key theoretical principles of EPIC-Neck (e.g., functional goals and behaviour-based discharge criteria) and what they felt "worked" in practice (e.g., symptom-based decisions).

> *"Sometimes you've got to shift things so you can still fit a standard appointment…I think as physio's we like to kind of think we know what the patient needs and we give them that goal as it's the least path of resistance is beneficial for us though. But it might not be beneficial to them on the long term." PT3 - 0-2 years' experience*

Overall, intervention features supported fidelity when they aligned with physiotherapists' clinical reasoning and perceived relevance, but constrained fidelity when viewed as complex, low value, or misaligned with patient characteristics.

Theme 4 – Physiotherapists adapted delivery based on patient engagement and perceptions (Patient factors)

Patient factors acted as a barrier across six checklist components. Physiotherapists reported adapting delivery based on their perceptions of patient characteristics, preferences, engagement, expectations, and understanding. Communication barriers, such as language differences or use of interpreters, influenced delivery, while perceived patient resistance or low engagement shaped how physiotherapists adjusted conversations or modified intervention components.

> *"I find it really difficult and uncomfortable to apply it to non-English speaking patients…I feel like so much of the training in general, talks about how we should ask certain questions, how we ask open-ended questions, and I don't know how well they are getting translated. It's so specific in our language but through an interpreter you can't be because you don't know what they're saying, you don't mandate them… obviously, we don't even know if they're any good. That's a challenge" PT3 - 0-2 years' experience*

> *"There's people who come with expectations that they're going to be told what to do because you're the professional, and then when you're trying to get out of them how safe do you feel doing these exercises, what the benefits, how often you're gonna do them and they are like well you tell me that's why I'm here" PT6 - 0-2 years' experience*

Some patients were thought to be unfamiliar with components like goal setting, struggled to articulate goals, or were perceived as uninterested in topics such as social support, leading physiotherapists to selectively adapt delivery. Overall, fidelity was negatively affected when there were communication barriers or physiotherapists perceived patients as resistant to the intervention components.

Theme 5 – Physiotherapist and patient-facing resources supported delivery when actively used (Resources)

Supporting resources influenced fidelity, acting as both barriers and facilitators across two checklist components each. Physiotherapist-facing resources, including crib sheets, proformas, and infographics, were accessible, easy to use, and provided prompts and structure for delivery, but inconsistent use and occasional forgetting to use them.

> *"The guides that we were given were helpful, definitely just having them somewhere visible, but even then, still I forgot. You know, sometimes would forget to glance at them and would forget things and do things and revert back to my old ways of interviewing or assessing." PT2 - 11-20 years' experience*

Patient-facing resources, such as workbooks, exercise libraries, and friends and family leaflets, helped structure delivery, prompted key content, and expanded exercise options, though physiotherapists sometimes perceived simply handing out materials as sufficient for fidelity.

> *"I used the workbook to highlight different sections, and it was a way that they could see, and I could see that we'd ticked off certain aspects of it… like we've addressed flare up management and you talked to me about this and we were able to implement this." PT1 - 6-10 years' experience*

Overall, resources supported fidelity when actively and consistently used but offered limited benefit when relied on passively.

Theme 6 - System design and pressures influencing optimal delivery (Organisational factors)

Organisational factors acted as a barrier across two checklist components. Physiotherapists described service structures and system pressures that constrained delivery, including high caseloads, short appointment times, and targets emphasizing rapid discharge. These pressures limited opportunities for sustained patient engagement, skill-building, and consistent application of the EPIC-Neck programme, shaping what was realistically achievable in practice.

> *"We discharge patients prematurely in the premise of efficiency… you're doing alright, off you go…It's just about trying to get people discharged…there's not enough emphasis on it being a long-term condition as this would be a massive shift in service delivery" PT5 - 6-10 years' experience*

Overall, fidelity was constrained where organisational demands conflicted with optimal delivery.

RECEIPT FIDELITY

Data S4 - Interview themes and illustrative quotes

PO1 – Performing exercise

Theme 1 – Most participants understood and felt confident, but complex or resisted exercises reduced clarity and confidence

Participants generally described understanding how to perform their exercises and feeling confident doing so. However, confidence and clarity were lower when exercises were more technically complex or required resistance, leading some participants to feel unsure about their technique.

> *“I understood how to do the exercises, but not straight away. Especially the band one that was difficult…The way you had the band around your head and then this action when you push it back. It was just something strange and unusual” P09: 30s, White, Mid IMD, Moderate disability*

Theme 2 – Safety was generally understood, but fear of harm persisted for some and could be significant

Most participants understood that the exercises were safe to perform. However, for a smaller number, fear of causing further pain or damage remained a significant concern undermining confidence and, at times, causing distress or avoidance. For some, fear eased as they gained experience and confidence with the exercises, but for others it persisted.

> *“I was really concerned about tackling the exercises initially thinking well this is just going to make it worse…I didn’t want to do too much and overdo it.” P02: 50s, Black, Low IMD, Mild disability*

> *“My worry was doing something seriously bad to myself and end up paralysed… or breaking my neck and I won’t be able to walk or lift my arm. But then I realised that exercise is not that actually that bad for you and I'm not gonna end up doing something really bad.” P11: 40s, White, Low IMD, Mild disability*

Theme 3 – Multiple sources supported understanding, but physiotherapist demonstrations were most valued

Participants described a range of supports that enhanced their understanding and confidence, including verbal explanations, written or pictorial instructions, practice, and feedback from their physiotherapist. Participants consistently emphasised that seeing the physiotherapist demonstrate exercises in person was an effective way to build clarity and confidence.

> *“He himself demonstrated how the movement should be done. Seeing it visually helped me. After going away, I thought, this is how he did it, I’ve got this image of what he did.” P06: 60+, Black, Low IMD, No disability*

PO2 - Adapting and progressing (or regressing) exercise

Theme 1: Most participants knew how to progress/regress exercises, but gaps remained

Participants generally understood how to make exercises harder or easier, most often by changing repetitions, but this knowledge was not universal.

“Set a certain number… say 10 then increase to 20 if you find it easier” P06: 60+, Black, Low IMD, No disability

“I don’t think I’ve necessarily made it harder… I don’t know if I knew how” P03: 30s, White, Low IMD, Moderate disability

Theme 2: Symptom response and exercise difficult prompted change

Participants understood that symptom responses and exercise difficulty were indicators to make exercise harder or easier.

“…if I felt worse, I would do lighter weights instead of not doing anything” P09: 30s, White, Mid IMD, Moderate disability

“…once it's started to reduce, you can increase the challenge” P10: 30s, Other, Low IMD, Mild disability

Theme 3: Understanding was shaped by prior experience and physiotherapist input

Understanding drew from general exercise experience and physiotherapist interactions.

“It's a bit like going to the gym. You need to increase your weight or reps in the gym to actually get physically stronger. And that same applies to the neck.” P08: 50s, Mixed, Mid IMD, Mild disability

“…the physio asked, ‘what do you need to do?’…He didn’t tell me, he guided me” P06: 60+, Black, Low IMD, No disability

PO3 - Self-managing flare-ups with exercise

Theme 1 - Understanding of flare-up management using exercise was inconsistent and often incomplete

Participants varied in their understanding of how to manage flare-ups with exercise. Some described accurate strategies aligned with the intervention, others reported approaches inconsistent with the protocol, and a few showed no understanding at all. While some participants intended to use exercise to manage flare-ups, others prioritised medication or seeking health care professional support

“…But I realise they work and if it did start to flare up again, I would go straight back to those exercises again” P01: 60+, White, Low IMD, Mild disability

“…you will get flare-ups but obviously it makes sense to just stop exercising” P02: 50s, Black, Low IMD, Mild disability

“What would you do if it got worse again? First of all, see the GP, definitely” P05: 40s, Asian, Low IMD, Moderate disability

Theme 2 – Physiotherapist input and workbook engagement shaped understanding

Physiotherapists were reported as influencing understanding, particularly by directing participants to the workbook for flare-up guidance

“…He did say to me it's in your book, read this section if you're having a flare-up…” P12: 80+, White, Low IMD, No disability

PO4 - Engage in general exercise and understand the benefits and risks of secondary prevention exercise

Theme 1 – Desire for general exercise was common, but actionable plans were lacking

Participants expressed interest in general exercise and recognised its benefits. Many did not know how to fit exercise into their daily lives or overcome personal barriers. Tailored action plans were lacking, leaving intentions largely aspirational

> *"There's always plans [laughs]… I find it hard to do half a day doing something and then think 'oh I'll go to the gym now'. So, I'd like to do it first thing in the morning but that isn't really feasible because it's too busy, you can't get on the equipment, and you can't get a car parking space" P01: 60+, White, Low IMD, Mild disability*

Theme 2 – Understanding of the risks and benefits of secondary prevention exercise was limited and decision-making lacked clear rationale

Participants showed limited understanding of the purpose, benefits, and risks of continuing neck-specific exercises once symptoms improved. Decisions to continue or stop were shaped by personal beliefs, convenience, or physiotherapist suggestion rather than by an informed understanding of secondary-prevention principles.

> *"It's become part of my routine now because if I don't, then there's chance it's going to get worse…its preventative as opposed to only doing it if I'm in discomfort because that taking steps backwards. Working on it day-to-day is hopefully gonna prolong or prevent you feeling the same as you were at the beginning." P09: 30s, White, Mid IMD, Moderate disability*

ENACTMENT FIDELITY

Data S5 - Interview themes and illustrative quotes

PO1 – Performing exercise

Theme 1 – Enactment was selective, shaped by feasibility, complexity and convenience

Participants generally enacted their prescribed exercises but adapted or prioritised them, performing those that were easier, less painful, or more convenient. Exercises that were simple to perform and easy to integrate into everyday routines (e.g., seated cervical retractions) were maintained most consistently, whereas those requiring specific positions, equipment, or higher technical challenge (e.g., four-point kneeling, scapular protraction, resisted exercises) were more often avoided. This flexibility helped maintain engagement but reduced fidelity to the prescribed programme and intended dosage.

> *"I cut them up instead of doing them all in one go, for example I could do the ones in sitting while I'm working" P10: 30s, Other, Low IMD, Mild disability*

> *"I found I didn't have the time to do them all in one day, so I spread them...I'm not doing the five of them every day" P12: 80+, White, Low IMD, No disability*

> *"Where you've got to get into the kneeling position onto the floor…you have to take time out for those, so you just leave them for another time and don't come back to them." P04: 50s, Asian, Low IMD, Moderate disability*

> *"It's easy for me to bring it into my day-to-day routine rather than having to stop work to do it. If I was having to stop and go away to do them it causes disruption which is a problem" P09: 30s, White, Mid IMD, Moderate disability*

> *"I've stopped doing the time consuming one, but I'm still doing the other ones that don't take much time. The one I found most challenging to do logistically...when I felt like I'd improved enough I stopped that one" P10: 30s, Other, Low IMD, Mild disability*

Theme 2 – Behavioural strategies such as piggybacking, prompts and routines supported enactment

Participants who established a regular routine for performing their exercises, or "piggybacked" them onto existing daily habits, described more consistent adherence. Prompts were also commonly reported such as using pain as a cue, placing visual reminders in the environment, or linking exercises to another activity.

> *"I do them before and after gym. It really helps me do my exercises." P07: 20s, Asian, Low IMD, Moderate disability*

> *"So do them at start of the day and leave the materials somewhere that I'm going to be able to see so I don't forget." P10: 30s, Other, Low IMD, Mild disability*

Theme 3 – Symptom responses reinforced or discouraged engagement

Participants were more likely to maintain exercise routines when they experienced symptom improvements, particularly immediate pain relief. Conversely, exercises perceived to worsen symptoms were often discontinued. In some cases, initial scepticism shifted once benefits became apparent.

"When the body responds well, that's good, it gives me more encouragement" P05: 40s, Asian, Low IMD, Moderate disability

"One of them actually aggravated the pain. I wonder if was actually putting too much pressure on my neck or whether I had got the technique right, but it didn't seem to make a difference, and I stopped doing that exercise." P02: 50s, Black, Low IMD, Mild disability

PO2 - Adapting and progressing (or regressing) exercise

Theme 1: Appropriate progression and/or regression was enacted inconsistently

Participants varied in applying progression principles. Some adjusted appropriately (typically via repetitions), others potentially reported missed opportunities to modify and other made no changes.

"I started off with 3 x 6 and I built it up to 3 x 20" P12: 80+, White, Low IMD, No disability

"Did you try and make the exercises harder yourself? No, I didn't" P05: 40s, Asian, Low IMD, Moderate disability

"I probably pushed myself too much...even if it's painful, I'm still trying…I would fight through pain…I don't actually know why…" P11: 40s, White, Low IMD, Mild disability

Theme 2: Fear and time limited change

Many hesitated to progress due to fear of pain or damage, particularly when symptoms had improved. Increasing repetitions was also seen as impractical or too time-consuming.

"I was thinking if I do 20, that could be pushing it and aggravate things. It's currently helping so I don't want to overdo it and exacerbate the pain." P02: 50s, Black, Low IMD, Mild disability

"It's just the time… when you're build up the reps of each one and trying to do that when we just come back from work it. It doesn't fit my lifestyle." P08: 50s, Mixed, Mid IMD, Mild disability

Theme 3: Benefits and physiotherapist guidance are key facilitators

Symptom improvement, reduced time and physiotherapist advice or exercise diaries facilitated change.

"Instead of adding a lot more sets, using that resistance band allowed me to condense the time and raise the challenge" P08: 50s, Mixed, Mid IMD, Mild disability

"The exercise diary made me think about whether this was achievable and a challenge" P02: 50s, Black, Low IMD, Mild disability

PO3 - Self-managing flare-ups with exercise

Theme 1 – Appropriate use of exercise strategies varied

Participants who experienced flare-ups did not consistently apply exercise strategies. Some followed physiotherapist/intervention protocol advice while others made no changes or used inappropriate strategies

> *"I was advised by the physio to reduce the number of reps I was doing, and then re-increase them, which is what I did" P08: 50s, Mixed, Mid IMD, Mild disability*

> *"Did you change the exercises when you had a flare-up? No… I just carried on and pushed through it." P11: 40s, White, Low IMD, Mild disability*

Theme 2 – The workbook was a key facilitator when used

Participants who engaged with the workbook valued it and used it repeatedly for flare-up management

> *"…I refer back to the handbook and check how to manage it…I've looked at the flare ups more than I've looked at any other part" P08: 50s, Mixed, Mid IMD, Mild disability*

PO4 - Engage in general exercise

Theme 1 – General exercise was rarely enacted due to barriers and limited problem-solving

No participant reported starting new or resuming prior general exercise routines as a result of the intervention. While common barriers were cited (time, anxiety etc) the fear of pain or re-injury was particularly influential. Participants lacked strategies to overcome barriers.

> *"I'd like to get back to the gym… I've not been for a while because of this neck problem… I was scared to do anything in case I made it worse" P11: 40s, White, Low IMD, Mild disability*

Patient-facing resources

Theme 1 – Engagement with patient-facing resources was limited, selective and often short-lived

Most resources were underused, except the exercise library. Use was selective and discontinued once routines were established or symptoms improved.

> *"I must admit, I've just been using the library. I haven't read the workbook if I'm brutally honest." P02: 50s, Black, Low IMD, Mild disability*

> *"I started to do it, but then it started to work so I stopped writing it down" P06: 60+, Black, Low IMD, No disability*

Theme 2 – The exercise library was valued but not fully optimised

The library acted as a reminder of exercises and technique. The use of pictures, text and video was well received. Providing a catalogue offered opportunity to attempt non-physio prescribed exercises but fear of causing harm limited experimentation, and the volume of exercises presented made it difficult to know which exercises they should be doing.

> *"I've referred back to the library to familiarise myself with which ones I was expected to do, and I just see the picture and I think ohh, that's the one" P08: 50s, Mixed, Mid IMD, Mild disability*

> *"I stuck with the ones we'd star…what if I do the others wrong… I'd just end up [expletive] myself up more because I'm not getting it right" P09: 30s, White, Mid IMD, Moderate disability*

Theme 3 – Physiotherapists are key to facilitating resources use

Engagement was facilitated when physiotherapists introduced and referenced them during appointments. Participants suggested integrated resources into consultations to increase engagement and emphasise key learning.

> *"I was introduced to the resources but maybe during the session if there was a more focus on what was there and what I can get from it…maybe if it was made part of the session plan" P03: 30s, White, Low IMD, Moderate disability*

Theme 4 – App engagement was minimal and dependent on physiotherapist facilitation

There was no evidence of app usage through the interviews. Some declined the app, but some expressed a preference for the app but not provided the opportunity to use it by their physiotherapist. This was due to physiotherapist preference and digital literacy.

> *"I wasn't given access to the app because the physio told me that she didn't use it, she used the workbook…having an app would have made things easier, having the Resources in my pocket all the time" P10: 30s, Other, Low IMD, Mild disability*
