# Supplementary material for: Multidimensional fidelity of the EPIC-Neck exercise programme for chronic non-specific neck pain: a UK feasibility trial process evaluation
Source: BMJ Open. 2026 Oct 20;16(10):e121944. doi: 10.1136/bmjopen-2026-121944 (PMC13684230; doi:10.1136/bmjopen-2026-121944)
Supplement: Supplementary file 4 [file bmjopen-16-10-s004.pdf]

INTERVIEW TOPIC GUIDES

Physiotherapist interview topic guide – fidelity and acceptability

Introduction and welcome

Physios were reminded of the purpose of the interview, the voluntary nature of participation, and confidentiality. They were informed there were no right or wrong answers and were encouraged to share honest views about their experiences of taking part in the EPIC-Neck study and completing the exercise programme.

Question 1 – General experience

"Tell me about your experience delivering the EPIC-Neck programme"

Question 2 – Effectiveness and refinement

"Do you think the EPIC-Neck programme will lead to more effective exercise and engage chronic non-specific neck pain patients more in their exercises?"

Optional prompts/follow-ups

What parts in particular do you think make the programme more effective and help with patient engagement?

In what ways should we adapt the programme to make it more effective and/or help with patient engagement?

Probe logic model

Question 3 – Coherence, Ethicality, Affective attitude

"In what ways does the EPIC-Neck programme align with your usual approach or beliefs relating to exercise prescription for chronic non-specific neck pain?"

Optional prompts/follow-ups

What aspects of the EPIC-Neck approach were added to your usual practice – how did it differ?

Were there any aspects that conflicted with how you would usually manage patients? Why do you think that was?

Do you feel motivated to continue with any aspects of the EPIC-Neck programme as part of your normal practice? If so, which bits. If not, why not?

Question 4 – Burden, Coherence, Cost

"How did you find delivering the EPIC-Neck programme alongside other clinical duties, assessments or treatment?"

Optional prompts/follow-ups

What specific challenges, if any, did you face when delivering the EPIC-Neck programme?

Question 5 – Training and Resources

"How well did the training, ongoing support and physiotherapist resources prepare you to deliver the EPIC-Neck programme confidently and effectively?"

Optional prompts/follow-ups

In what ways did your confidence change after having the chance to deliver the intervention with real patients?

What aspects of the training or support were most or least helpful?

Which resources were most helpful, or least helpful, and why? (Prompt crib sheets, infographics, therapist manual etc)

What changes could be made to make the training or physiotherapist resources to make them more effective?

Question 6 – Fidelity

"I'd like to discuss some of the audio-recordings we reviewed. We noticed some challenges with delivering [XXX] consistently. Why do you think this was the case?"

Optional prompts/follow-ups

How important do you think XXX is to the success of the EPIC-Neck programme?

What strategies could be used to help physiotherapists deliver XXX more consistently and effectively?

N.B – Repeat for key areas of poor fidelity delivery

Question 7 – Final refinement and comments

"Finally, if you were in my position, what changes or improvements would you make to the EPIC-Neck programme to make it more effective for patients, help them engage with it more effectively, or assist physiotherapists in delivering it more smoothly?"

Patient participant interview topic guide – fidelity and acceptability

Introduction

Participants were reminded of the purpose of the interview, the voluntary nature of participation, and confidentiality. They were informed there were no right or wrong answers and were encouraged to share honest views about their experiences of taking part in the EPIC-Neck study and completing the exercise programme.

Section 1 – Exercise programme general

Main question

“Can you tell me about you found the exercise programme the physiotherapists gave you for your neck pain?”

Optional prompts/follow-ups

“What sort of exercises were you doing”

“How many were you supposed to be doing?”

“Were you able to do your exercises as much as the physiotherapists suggested?”

Yes - “what helped you do you exercises?”

No - “what made it difficult for you to do your exercises?”

Probe logic model components

"Were there any parts of the exercise you found confusing or hard to follow?"

Yes - Which bits in particular? “What could have been done differently to help you understand better?”

No - Was there anything about what the physiotherapist said/did or the resources that helped you understand what you were doing

Section 2 - Acceptability

Opportunity Costs

"How did you find doing the exercises around other important things you need to do in your life?”

Optional prompts/follow-ups

"Did you find ways to manage or work around these challenges? What helped, if anything?"

“What helped you fit the exercises into your routine, if anything?"

Burden

“Was there anything about the exercises that made it feel too difficult or too much work?”

Optional prompts/follow-ups

“Can you give me some examples”

“Was there anything that made the exercises easier or more manageable for you?"

Safety

"Was there anything about the exercises that you were not confident about, or that made you feel unsure or even unsafe?"

Optional prompts/follow-ups

"What specifically made you feel unsure or unsafe? Can you give me an example?"

"Was there anything about the exercises, the resources or what the physiotherapists did with you that helped your confidence or made you feel safe?"

General acceptability

"What are your thoughts about the exercises overall? How did they feel for you?"

"Can you describe what you liked or didn't like about the exercises?"

Section 3 – Benefits

"Did the exercises help your neck pain or any other aspects of your health?"

Probe – Outcomes (pain, physical, psychological, sleep, relationships)

Optional prompts/follow-ups

"Could you tell me in what ways the exercise programme helped you?"

"What aspects of the exercises did you find helped you the most?"

"When did you start to see benefits?"

"Tell me about how confident you were at the beginning as to whether exercise would help, and tell me how that changes as treatment went on"

"Can you tell me more about what you expected from the exercises and how that might have been different from what you experienced?"

"Why do you think the exercises didn't help you?"

"What do you think could have made the exercises more effective for you?"

"Even though it didn't help you, how do you think it could help others?"

"Did you understand why you were doing the exercises and how they might help with your neck pain?"

"Was there anything in particular the physios said or did that you found helpful to understand why you were doing the exercises and how they might help?"

"Was there anything about the resources (e.g., workbook, diary etc.) that helped you understand how the exercises would benefit you?"

"What would have helped you better understand why you were doing the exercises and how they would help your pain?"

Section 4 – PO2 - Progression and adaptation

"One of the ideas in the programme is that you are able to change your exercises by yourself to make them harder or easier as needed. Could you tell me how you would do that?" / "How have you done that?"

Optional prompts/follow-ups

"What parts of the physiotherapist's instructions, or resources made it easier for you to know how to change the exercises?"

"What problems did you have when trying to change the exercises or make them harder?"

"What further support would you need to understand and be able to progress exercise independently?"

Probe logic model

Section 5 – PO3 - Flare-ups

"The program also talked about using exercise to manage pain flare-ups. What would you do if you have a flare-up?"/"What have you done?

Optional prompts/follow-ups

"How did you use exercise to manage the flare-up?"

"Was there anything that helped you use exercise to manage the flare-up?"

"What would you do if you did have a flare-up in future?"

Probe logic model

Section 6 – PO4 – General exercise and secondary prevention

"Could you tell me about what your future plans are in relation to your exercise programme and your neck pain?"

"Will you continue with the exercises from the physiotherapist?"

"Do you plan to do any other general exercise?" "Why or why not?"

Optional prompts/follow-ups

What has helped you make that decision?

What has helped you make that decision?

Probe logic model

Section 7 – Resources

"How did the resources like the workbook, app and other information help you with your exercise programme?"

Optional prompts/follow-ups

"What did you find most helpful"

"Were there any resources that didn't help you?" "Why?"

"Is there anything we could improve or change about these resources?"

Section 8 – Physio support

"How could physiotherapists improve the support they gave you during the exercise program?"

Optional prompts/follow-ups

"Was there anything about the physiotherapist's advice about exercise that was especially helpful?"

“Were there times when you felt unsure about what to do? How could the physiotherapist have helped more?”

Probe logic model

Section 9 – Study design

“What could we change in this study to make it easier for people with neck pain to take part?”

Optional prompts/follow-ups

"Was the information you received before the study clear? Did it help you decide whether to take part, and did it match what you experienced?"

“Were there any parts of the study difficult or inconvenient for you?” Prompt postal questionnaire
