# Supplementary material for: Multidimensional fidelity of the EPIC-Neck exercise programme for chronic non-specific neck pain: a UK feasibility trial process evaluation
Source: BMJ Open. 2026 Oct 20;16(10):e121944. doi: 10.1136/bmjopen-2026-121944 (PMC13684230; doi:10.1136/bmjopen-2026-121944)
Supplement: Supplementary file 5 [file bmjopen-16-10-s005.pdf]

GRIPP2-SF

1. Aim of PPIE

The purpose of PPIE in this study was to ensure the fidelity evaluation:

- aligned with patients' beliefs, expectations, and lived experiences of exercise-based care;
- was practical, acceptable, and feasible for patients to engage with;
- evaluated the intervention components and performance objectives in a way that reflected what matters to patients;
- supported decisions about how to describe, prioritise, and share findings with patient and public audiences.

PPIE also helped shape how results were interpreted (e.g. the importance of focusing on performance objectives, particularly PO4) and contributed to the development of the PUCA dissemination principles (Placement, Understandable, Credible, Attention-grabbing).

2. Methods of PPIE

Recruitment and composition

Participants for the PPIE group were recruited through clinic advertisements, social media, community signposting, and word-of-mouth. This helped achieve diversity in age, gender, ethnic background, and activity levels. We made targeted efforts to reach South Asian communities to reflect the population of Birmingham. All contributors were compensated for their time, and travel and parking costs were covered.

Format and facilitation

Workshops were held in person and facilitated by the lead author (JP), supported by clinicians, academics, and researchers experienced in PPIE and focus-group facilitation. Sessions used participatory activities (e.g., walking brainstorms, dot voting, round-robin discussions, practical tasks). Each workshop began with a brief recap to help maintain continuity for people who could not attend every session.

Ensuring meaningful involvement

Facilitators ensured all contributors, including quieter members, had space to speak. PPIE involvement spanned the whole project: multiple workshops during intervention development, one workshop before the fidelity study began, ongoing oversight during the study, and a final workshop focused on interpretation and dissemination. Two PPIE contributors sat on the Study Management Group.

3. Results of PPIE Contribution

Study design

- Highlighted the need to describe "usual exercise care" so fidelity findings could be interpreted meaningfully.
- Recommended interviewing patients at the end of treatment (rather than 4 months post-randomisation) so they had genuine exposure to performance objectives before being asked about them.

Participant communication and recruitment

- Co-developed the Participant Information Leaflets (PILs) and invitation materials (wording, layout, accessibility, cultural relevance).
- Suggested video-based PILs in English and Urdu, rather than written translations, to reduce literacy barriers.
- Provided suggestions to improve recruitment processes when initial uptake was low.
- Advised on ethnicity categories, recommending detailed categorisation aligned with UK census format.

Measures and data collection

- Reviewed and refined questionnaires:
    - added open-text boxes,
    - reduced length,
    - recommended one question per performance objective.
- Reviewed interview topic guides: confirmed the length was appropriate and helped refine question wording, particularly around eliciting participants' understanding of the exercise programme.
- Discussed acceptability of audio-recording physiotherapy appointments; the group felt this was acceptable if clearly explained as recording the physiotherapist, not the patient.

Study oversight and participant engagement

- Contributed to participant newsletters (study progress updates and reminders), which supported questionnaire return.
- Provided feedback at interim stages and helped identify areas requiring modification during the study.

Analysis and interpretation

- Helped prioritise the fidelity results most relevant to patients (e.g., focusing on performance objectives rather than every individual component).
- Identified the importance of understanding why some participants did not engage with PO4 (general exercise).

Dissemination

- Contributed to the development of dissemination principles for patient and public audiences (PUCA framework).

4. Discussion and conclusions

PPIE input directly led to:

- more acceptable and accessible study materials (PILs, questionnaires, topic guides, video PILs);
- more culturally detailed demographic categorisation;
- improved recruitment strategy and participant retention;
- a change in interview timing to better capture fidelity;
- refinement of the fidelity evaluation (e.g., including usual-care comparison);
- clearer patient-centred framing of the results (performance-objective focus);
- practical guidance for future dissemination approaches.

A summary of PPIE contributor characteristics is shown in the table below.

		N	%
Age			
	30-39	7	41%
	40-49	4	24%
	50-59	1	6%
	60-69	4	24%
	70+	1	6%
Gender			
	Female	10	59%
	Male	7	41%
Ethnic Background			
	White British	8	47%
	White Other	1	6%
	Asian Pakistani	3	18%
	Asian Indian	3	18%
	Black African	1	6%
	Black Caribbean	1	6%
Index of Multiple Deprivation Decile			
	1 (most deprived)	5	29%
	2	2	12%
	3	2	12%
	4	3	18%
	5	-	-
	6	1	6%
	7	-	-
	8	-	-
	9	3	18%
	10 (least deprived)	1	6%
Physical Activity Levels			
	Inactive	6	40%
	Moderately Inactive	2	13%
	Moderately Active	4	27%
	Active	3	20%

5. Reflections / Critical Perspectives

PPIE involvement was extremely valuable and enhanced the acceptability, relevance, and quality of the fidelity evaluation. However:

- The group were highly engaged and motivated; their experiences may not fully represent patients who struggle with engagement, who are particularly important in fidelity and implementation research.
- Views within the group were sometimes conflicting, requiring careful negotiation and prioritisation.

- Enthusiasm occasionally led to a large number of suggestions, and facilitators needed to balance ambition with methodological and resource constraints.

Overall, PPIE made a substantial contribution to shaping a more patient-centred fidelity evaluation and improving the design, conduct, interpretation, and dissemination of the study.
