# Supplementary material for: Multidimensional fidelity of the EPIC-Neck exercise programme for chronic non-specific neck pain: a UK feasibility trial process evaluation
Source: BMJ Open. 2026 Oct 20;16(10):e121944. doi: 10.1136/bmjopen-2026-121944 (PMC13684230; doi:10.1136/bmjopen-2026-121944)
Supplement: Supplementary file 6 [file bmjopen-16-10-s006.pdf]

TITLE PAGE

Full Title: Exercise Prescription Improved through Co-design for people with chronic non-specific Neck pain (EPIC-Neck): A randomised feasibility study with process evaluation

Internal Reference/Short Title: EPIC-Neck

Version number: v2.0

Version date: 12/02/2024

IRAS number: 331102

Sponsor: Birmingham Community Healthcare NHS Foundation Trust

Sponsor reference number: BCHCASR331102.P

Funder(s): HEE/ NIHR ICA Programme (Clinical Doctoral Research Fellowship; NIHRXXXXXX)

This protocol has regard for the HRA guidance

CONTENTS

List of abbreviations 7

Study summary 8

Funding and support in kind ....**Error! Bookmark not defined.**

Role of the study sponsor and funder... 11

Roles and responsibilities of study management commitees /groups and individuals 12

Study flow chart 13

1 Background 14

2 Rationale 14

2.1 Review of existing evidence 14

3 Aims and objectives 15

3.1 Aim 15

3.2 Main feasibility objective (large-scale definitive trial progression criteria) ... 16

3.3 Other feasibility objectives 18

4 Study design and setting 18

4.1 Study design 18

4.2 Study setting 19

5 Eligibility 19

5.1 Inclusion criteria 19

5.2 Exclusion criteria 19

6 Enrolment, consent, randomisation and blinding 19

6.1 Identification of participants 19

6.1.1 NHS musculoskeletal waiting lists and new referrals 19

6.1.2 NHS musculoskeletal department advertisements 20

6.2 Face-to-face screen and research appointment 20

6.2.1 Screening 20

6.2.2 Consent ... 20
6.2.3 Baseline questionnaire ... 21
6.2.4 Randomisation ... 21
6.2.5 Blinding ... 22
6.3 Participant screening and recruitment log ... 22
6.4 Payment ... 22
6.5 Physiotherapist process evaluation consent procedures ... 23
6.5.1 Physiotherapists delivering the EPIC-Neck intervention ... 23
6.5.2 Physiotherapists delivering the Usual exercise care intervention ... 23
7 Study interventions ... 24
7.1 EPIC-Neck programme (Intervention arm) ... 24
7.1.1 Development ... 24
7.1.2 EPIC-Neck programme components ... 28
7.1.3 EPIC-Neck programme delivery resources ... 29
7.1.4 EPIC-Neck programme structure and delivery ... 30
7.2 Usual exercise care (Control arm) ... 31
7.3 Training ... 31
8 Outcome measures ... 31
8.1 Primary outcome measure (for the future trial) ... 32
8.2 Secondary outcome measures (for the future trial) ... 32
8.3 Other outcome measures ... 32
8.4 Fidelity ... 32
8.4.1 Fidelity of delivery ... 35
8.4.2 Fidelity of receipt ... 35
8.4.3 Fidelity of enactment ... 35
8.5 Contamination ... 35
8.6 Qualitative interviews ... 36

8.6.1 Participant ... 36
8.6.2 Physiotherapist ... 37
9 Study procedures ... 37
9.1 Schedule of procedures ... 37
9.2 Baseline data collection ... 40
9.2.1 Participant ... 40
9.2.2 Physiotherapist ... 40
9.3 Intervention data collection ... 40
9.4 Follow-up data collection ... 41
9.4.1 Participant ... 41
9.4.2 Physiotherapist ... 42
9.5 Withdrawal criteria ... 42
10 Safety reporting ... 42
10.1 Definitions ... 42
10.2 Safety recording and reporting ... 43
11 Data handling and record keeping ... 44
11.1 Completed questionnaires ... 44
11.2 Data management, protection, and confidentiality ... 44
11.3 Archiving ... 45
12 Monitoring, audit, and inspection ... 46
13 End of study definition ... 46
14 Statistics and data analysis ... 46
14.1 Sample size calculation ... 46
14.2 Statistical analysis plan ... 46
15 Study organisational structure ... 47
15.1 Sponsor ... 47
15.2 Study management group ... 47

16 Ethical considerations 47
17 Public and patient involvement and engagement 47
18 Financial and other competing interests 47
19 Insurance and indemnity 48
20 Post-study care 48
21 Access to final study data set 48
22 Publication plan 48
23 References 48

LIST OF ABBREVIATIONS

Abbreviation	Term
AE	Adverse Event
AQoL	Assessment of Quality-of-Life
BCHC	Birmingham Community Healthcare NHS Foundation Trust
CI	Chief Investigator
CRF	Case Report Form
EPIC-Neck	Exercise Prescription Improved through Co-design for people with chronic non-specific Neck pain
GP	General Practitioner
HCI	Health and Care Innovation
HCP	Healthcare Professional
HEE	Health Education England
HRA	Health Research Authority
ICA	Integrated Clinical and Practitioner Academic
ICF	Informed Consent Form
IMD	Indices of Multiple Deprivation
IP	Intellectual Property
NDI	Neck Disability Index
NHS	National Health Service
NIHR	National Institute for Health and Care Research
PI	Principal Investigator
PIL	Participant Information Leaflet
PPIE	Patient and Public Involvement and Engagement
PSFS	Patient Specific Functional Scale
REC	Research Ethics Committee
SAE	Serious Adverse Event
SPIRIT	Standard Protocol Items: Recommendations for Interventional Trials
UK	United Kingdom
VAS	Visual Analogue Scale

STUDY SUMMARY

**Trial Title**	Exercise Prescription Improved through Co-design for people with chronic non-specific Neck pain (EPIC-Neck): A randomised feasibility study with process evaluation
**Internal ref. no. (or short title)**	EPIC-Neck
**Clinical Phase**	Feasibility study
**Trial Design**	A multicentre, two arm, parallel, individually randomised controlled feasibility study with process evaluation
**Trial Participants**	**Inclusion criteria**<br><br>1. Chronic non-specific neck pain (>3 months)<br>2. Aged ≥18 years old<br>3. Willing and able to give informed consent<br><br>**Exclusion criteria**<br><br>1. Received physiotherapy, chiropractic or osteopathic treatment (exercise, manual therapy, acupuncture etc) for neck pain in the past 6 months<br>2. Previous cervical spine surgery<br>3. Neck pain due to trauma (e.g., whiplash associated disorders)<br>4. Neck pain due to a specific diagnosis (e.g., rheumatoid arthritis, osteoarthritis)<br>5. Presence or suspicion of neurological involvement (e.g., cervical radiculopathy or cervical myelopathy)<br>6. Presence or suspicion of serious pathology requiring onwards referral<br>7. Headache as the primary complaint<br>8. Unable to understand or follow study procedures with support from family members or professional interpreters
**Planned Sample Size**	45 patient participants (30 EPIC-Neck programme; 15 Usual exercise care))<br>16-25 physiotherapist for process evaluation
**Study arms**	Patient participants will be individually randomised to either an interventional group (EPIC-Neck programme) or usual exercise care. Both groups will receive any other non-exercise usual care (e.g., work-place advice, postural advice, manual therapy).<br><br>**EPIC-Neck**<br>The EPIC-Neck programme is an evidence-informed, theory driven and co-developed biopsychosocial intervention to improve self-management of chronic non-specific neck pain through a home exercise programme.

<table>
<tr><td></td><td colspan="2">The EPIC-Neck programme consists of two main components:

1. A behavioural intervention aiming to maximise participant engagement in their neck exercise self-management programme.

2. A neck exercise intervention optimising exercise prescription to maximise short and long-term benefits.

The EPIC-Neck programme is delivered with supporting resources co-produced by the study team and patient representatives (EPIC-Neck workbook [paper or mobile application]; exercise sheets, wall chart and friends and family resource).

The EPIC-Neck programme is delivered face-to-face during physiotherapy appointments. Participants are expected to attend at least the initial physiotherapy appointment to be considered to have received the EPIC-Neck programme. There is no upper limit of appointments, and no defined timeframe treatment should be delivered within. Initial physiotherapy appointments and follow-ups will range from 45-60 mins and 20-30 mins respectively.

Usual exercise care

Physiotherapy exercise as per normal practice. As a pragmatic approach to the study design will be adopted, usual exercise care will be determined by the physiotherapist delivering the control-arm treatment.</td></tr>
<tr><td>Treatment duration</td><td colspan="2">See above</td></tr>
<tr><td>Follow up duration</td><td colspan="2">3- and 6-months post randomisation</td></tr>
<tr><td>Interviews</td><td colspan="2">EPIC-Neck participant semi-structured one-to-one interviews at 4-months post-baseline

Physiotherapist (EPIC-Neck and usual exercise care) semi-structured one-to-one interviews after the majority of treatment has been completed</td></tr>
<tr><td>Planned Study Period</td><td colspan="2">18 months</td></tr>
<tr><td></td><td>Objectives</td><td>Measurement</td></tr>
<tr><td>Main feasibility objective</td><td>Determine whether to continue to a</td><td>Evaluation of key uncertainties against pre-defined traffic light criteria
- Acceptability
- Delivery fidelity</td></tr>
</table>

	large-scale definitive trial	- Contamination<br>- Recruitment rate
**Other feasibility objectives**	To record and evaluate adverse events	Adverse event case report form
	To define usual exercise care for chronic non-specific neck pain	Usual exercise care audio-recordings<br><br>Usual exercise care observations completed by the CI<br><br>Interviews with physiotherapists delivering usual exercise care after the majority of treatment has been completed
	To deliver and gather feedback to help refine the physiotherapist training and intervention materials /procedures	EPIC-Neck training questionnaire<br><br>Interviews with physiotherapists delivering EPIC-Neck programme after the majority of treatment has been completed
	To refine the EPIC-Neck logic model	EPIC-Neck participant interviews at 4-months<br><br>EPIC-Neck follow-up questionnaires<br><br>Interviews with physiotherapists delivering EPIC-Neck programme after the majority of treatment has been completed
	To explore the demographics of people who do and do not enrol onto the study	EPIC-Neck Participant Screening and Recruitment log
	To measure the completeness of the planned definitive trial outcome measures at 6-months follow-up	Study monitoring data

ROLE OF THE STUDY SPONSOR AND FUNDER

As sponsors, Birmingham Community Healthcare NHS Foundation Trust (BCHC) are providing oversight of the conduct of the study. BCHC will review and approve study documentation produced by the research team and administer the grant for the research. BCHC staff have contributed to the design of this research and will be involved in data analysis and interpretation and decision making in relation to the choice of journal the work will be submitted to. BCHC may offer support with dissemination of the results and provide support with any further work following feasibility testing. BCHC will be the owners of any Intellectual Property (IP) generated by the research and are responsible for data protection in relation to all aspects of the research.

The NIHR are funding this research through the HEE/ NIHR ICA Programme; Clinical Doctoral Research Fellowship; NIHRXXXXXX.

ROLES AND RESPONSIBILITIES OF STUDY MANAGEMENT COMMITEES /GROUPS AND INDIVIDUALS

Patient and public involvement and engagement (PPIE)

A PPIE group consisting of 20 diverse members assisted with co-design and production of the EPIC-Neck programme and the development of this protocol and supporting patient information leaflet. 2-3 PPIE group representatives will sit on the Study Management Group.

EPIC-Neck mobile application developers (Health and Care Innovation Ltd)

Build the app and a web-based dashboard and make the changes required following initial review by co-design with PPIE representatives (prior to opening of feasibility and acceptability testing).

Ensure the app and dashboard are compliant with the NHS Digital Technology Assessment Criteria as described in the software specification so they are appropriate for current and future use in the NHS.

Continue to update and maintain the app and dashboard for a 2-year period so participants can continue to use it following the close of the study.

Protocol contributors

Professor Colin Greaves (employed by the University of Birmingham) will have overall oversight of the research, supporting the Chief Investigator with protocol development, application for ethical and HRA approvals, conduct of the research, data analysis, interpretation, and dissemination.

Mrs Natalie Rowland (employed by the University of Birmingham) and Professor Alison Rushton (employed by Western University, Ontario, Canada) will contribute to supporting protocol development, application for ethical and HRA approvals, conduct of the research, data analysis, interpretation, and dissemination.

They are independent of BCHC and the NIHR.

<u>BCHC (Sponsors)</u>

BCHC will review and approve study documentation produced by the research team. BCHC will be involved in data analysis and interpretation, manuscript writing, and decision making in relation to dissemination of results. BCHC are responsible for data protection in relation to all aspects of the research.

<u>PPIE representatives</u>

Public and patient involvement and engagement (PPIE) representatives who are people with chronic non-specific neck pain have been involved in developing the intervention and have reviewed this protocol and the patient information leaflets.

STUDY FLOW CHART

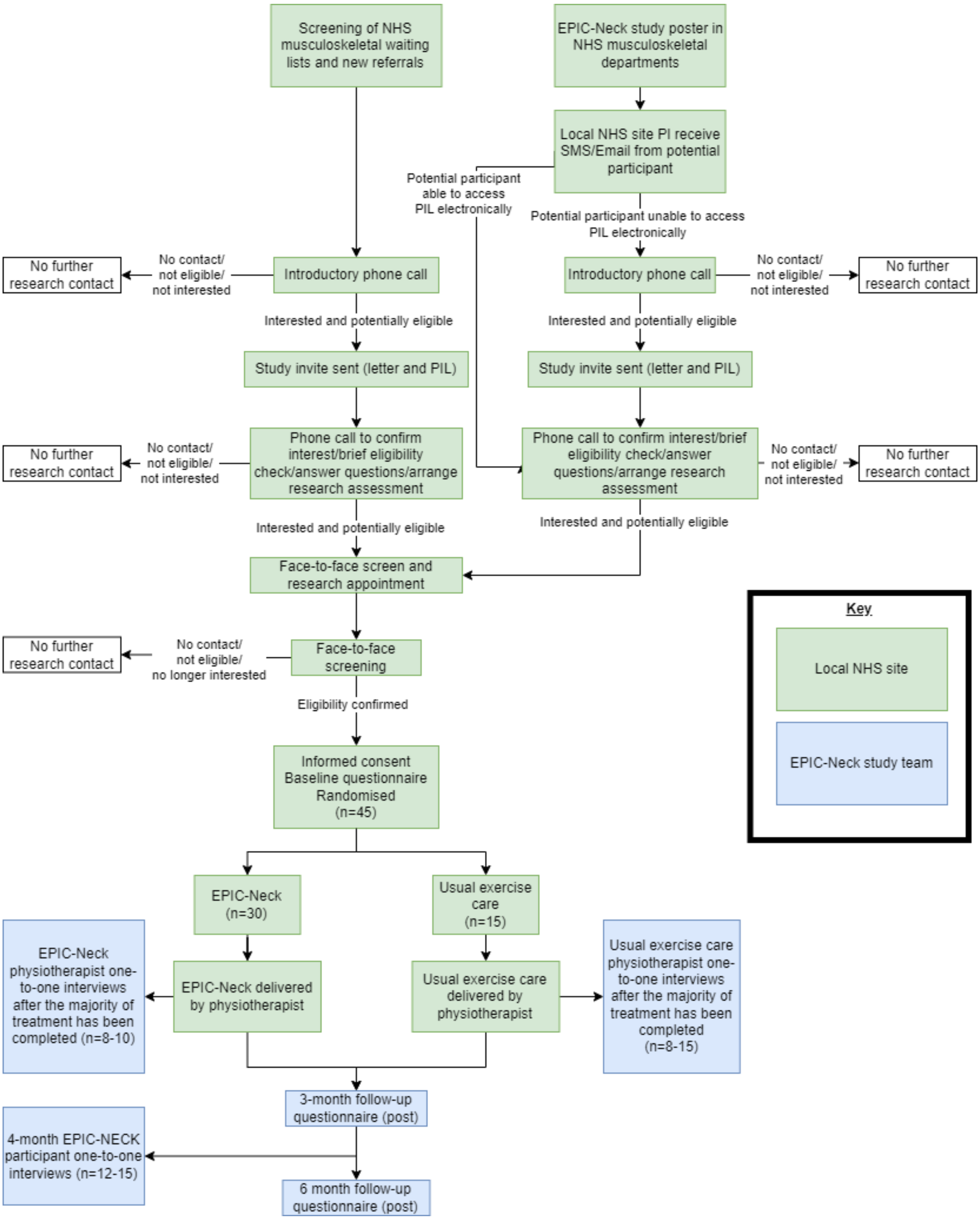

1 BACKGROUND

Neck pain is the third most common cause of years lived with disability in the United Kingdom (UK)(1) leading to personal financial burden (2) and poor quality of life (3). Personal impact is greatest for people with recurring or chronic, non-specific symptoms (chronic non-specific neck pain)(4) accounting for up to 75% of all people with neck pain (5, 6). These individuals will experience moderate to severe disability on 2 or more occasions each year (7).

International (8, 9) and National Institute for Health and Care Excellence (10) guidelines recommend people with chronic non-specific neck pain engage in long-term self-management of symptoms via physiotherapy led neck exercise programmes. Although physiotherapists follow treatment guidelines and prescribe neck exercise (11), the short-term effects of exercise on symptoms are modest (12, 13), engagement in self-management exercise is poor (13, 14), and 48% of people continue to experience long-term and recurrent episodes of disability (15). Current exercise programmes for people with chronic non-specific neck pain are inadequate.

Research is needed to 1) maximise the short-term effects of, and 2) increase engagement with self-management exercise to reduce long-term severity, duration and frequency of neck pain and disability.

2 RATIONALE

Improving exercise prescription for people with chronic non-specific neck pain to maximise effectiveness and engagement is a top ranked international neck pain research priority (16), James Lind Alliance UK physiotherapy research priority (17) and highlighted by the Global Burden of Disease study (18).

Patient and public involvement informing this study (20 chronic non-specific neck pain patients) identified the importance of exercise in the long-term self-management of neck pain disability episodes. Their experiences resonated with the existing evidence that available exercise programmes provide adequate short-term relief but fail to achieve long-term expectations or meet their needs for ongoing support.

2.1 Review of existing evidence

A neck exercise logic model would reduce heterogeneity and enable intervention refinement

A Cochrane review of exercise for neck pain (27 trials, 3005 participants)(12) and a recent neck exercise systematic review (19) identifies significant heterogeneity in exercise therapy between trials and poor reporting. This makes evidence synthesis to establish effect size difficult and provides very little guidance for clinical practice. The development of a neck exercise logic model would help intervention reporting, consistency, and reproducibility of neck pain exercise in clinical practice and intervention refinement in future trials (12, 20, 21).

A neck exercise logic model should optimise the technical aspects of exercise to maximise short-term effects

The Cochrane review of neck pain exercise (12), a review of reviews of chronic pain exercise (20) and other neck exercise systematic reviews (19, 22) demonstrate modest short-term effects, but recommend further optimisation of the technical aspects of exercise (dosage, mode, targets) to maximise benefits.

A neck exercise logic model needs to consider behavioural change strategies to maximise patient engagement to prescribed exercise therapies

Systematic reviews (20, 23, 24) demonstrate that long-term exercise engagement increases the effectiveness of exercise interventions for musculoskeletal pain and disability. Unfortunately, engagement to neck pain exercise programmes is poor (only 2/14 studies reported exercise adherence >75%)(13) particularly beyond 7-10 weeks (14). Although a Cochrane review of interventions for chronic musculoskeletal conditions demonstrates behavioural change strategies can improve exercise engagement (25), other systematic reviews identify a paucity of behavioural change strategies within neck exercise programmes (26).

The Cochrane review of exercise for neck pain (12), the Cochrane review of exercise for chronic pain (20) and the Medical Research Council (21) all recommend that chronic non-specific neck pain exercise interventions be co-developed with patients using a complex intervention framework to incorporate behavioural change techniques and address individual exercise barriers/facilitators.

Adequately powered long-term clinical trials are required to evaluate the long-term benefits of neck exercise

Systematic reviews of neck pain (12, 19, 22) and chronic pain exercise interventions (20) show that trials are predominantly limited to short-term follow up (<6 months) and evidence is downgraded due to small sample sizes (<70 per arm). Long-term, adequately powered trials are needed. Prior to conducting an expensive clinical trial with long-term follow up, feasibility testing is needed to ensure the trial procedures can be delivered successfully, with low attrition rates.

This feasible study is part of a body of work to co-develop an exercise programme using a complex intervention development framework (21). Intervention mapping principles (27) have been used to co-develop the EPIC-Neck programme, grounded in evidence and theory and based on a logic model aiming to optimise the short-term benefits of, and long-term engagement with neck exercise.

3 AIMS AND OBJECTIVES

This protocol is written using the suggested adaptations (28) to the SPIRIT (29, 30) and CONSORT reporting guidelines for feasibility studies (31).

3.1 Aim

The overall aims of this study are to evaluate the feasibility and acceptability of the EPIC-Neck programme and inform the design of a large-scale definitive trial evaluating clinical and cost-effectiveness of the EPIC-Neck programme.

3.2 Main feasibility objective (large-scale definitive trial progression criteria)

- The main feasibility objective is to determine whether to continue to a large-scale definitive trial. Based on recommendations for progression criteria in feasibility studies (32) four key areas of uncertainty will be evaluated against a traffic light system (Table 1).

Table 1 Traffic light progression criteria

- Delivery fidelity – The EPIC-Neck programme being delivered as intended.

- Contamination – The EPIC-Neck programme and usual care exercise are sufficiently different. Measured by the presence of the EPIC-Neck logic model key behavioural and exercise components within usual exercise care.

- Acceptability – The degree to which people delivering and receiving the EPIC-Neck programme consider it to be appropriate. Measured qualitatively and quantitatively using Sekhon's acceptability of healthcare intervention domains (33, 34).

- Mean recruitment rate – The mean rate of recruitment per month. Threshold criteria determined by calculating the time to recruit to an estimated sample size of 248 in a definitive trial using 5 musculoskeletal services (19). Green would be <18 months, amber would be 18-30 months, and red would be >30 months.

Table 1 Traffic light progression criteria

	Green	Amber	Red	Measurement
**Mean recruitment rate**	≥3 per month	1-2 per month	≤1 per month	Study monitoring data at treatment completion
**Acceptability**	Mean acceptability score of ≥ 4 and no areas of concern identified in qualitative interviews	Mean acceptability score 3 and/or minor problems identified in qualitative interviews	Mean acceptability score ≤2 and/or major problems identified in qualitative interviews	Acceptability questionnaire at 3- and 6-months (33)<br><br>EPIC-Neck participant interviews at 4-months<br><br>Interviews with physiotherapists delivering EPIC-Neck programme after the majority of treatment has been completed
**Delivery fidelity**	No areas of concern identified	Minor problems identified	Major problems identified	EPIC-Neck appointment audio recordings and observations
**Contamination**	No EPIC-Neck logic model key behavioural or exercise components present within usual exercise care	Minor problems identified	Major problems identified	Usual exercise care appointment audio recording and observations<br><br>EPIC-Neck appointment audio recordings and observations<br><br>Interviews and questionnaire with physiotherapist delivering usual exercise care after the majority of treatment has been completed
Green: No areas of concern<br>Amber: Minor problems that require attention before continuing to a definitive trial<br>Red: Major problems that require urgent attention before continuing to a definitive trial				

3.3 Other feasibility objectives

Other objectives for this feasibility study are in Table 2.

Table 2 Other feasibility objectives

Objective	Measurement
To record and evaluate adverse events	Adverse event case report form
To define usual exercise care for chronic non-specific neck pain	Usual exercise care audio-recordings<br><br>Usual exercise care observations completed by CI<br><br>Interviews with physiotherapists delivering usual exercise care after the majority of treatment has been completed
To deliver and gather feedback to help refine the physiotherapist training and intervention materials /procedures	Training evaluation questionnaire<br><br>Neck Exercise Questionnaire<br><br>Interviews with physiotherapists delivering EPIC-Neck programme after the majority of treatment has been completed
To refine the EPIC-Neck logic model	EPIC-Neck participant interviews at 4-months<br><br>EPIC-Neck follow-up questionnaires<br><br>Interviews with physiotherapists delivering EPIC-Neck programme after the majority of treatment has been completed
To explore the demographics of people who do and do not enrol onto the study	EPIC-Neck Participant Screening and Recruitment log
To measure the completeness of the planned definitive trial outcome measures at 6-months follow-up	Study monitoring data

4 STUDY DESIGN AND SETTING

4.1 Study design

A multicentre, two arm, parallel, individually randomised controlled feasibility study with process evaluation. Patient participants will be randomised to either an interventional group (EPIC-Neck programme) or usual exercise care. Both groups will receive any other non-exercise usual care (e.g., work-place advice, postural advice, manual therapy). The feasibility study will be conducted across two sites with a total of 45 patient participants recruited over a 12-month period with 6-month follow-up after randomisation. To ensure sufficient data is obtained for process

evaluation, participants will be randomised in a 2:1 ratio in favour of the interventional group (53).

4.2 Study setting

Recruitment will be from NHS community based and secondary care musculoskeletal services.

5 ELIGIBILITY

5.1 Inclusion criteria

1. Chronic non-specific neck pain (>3 months)
2. Aged ≥18 years old
3. Willing and able to give informed consent

5.2 Exclusion criteria

Individuals will be excluded for the following reasons

1. Received physiotherapy, chiropractic or osteopathic treatment (exercise, manual therapy, acupuncture etc) for neck pain in the past 6 months
2. Previous cervical spine surgery
3. Neck pain due to trauma (e.g., whiplash associated disorders)
4. Neck pain due to a specific diagnosis (e.g., rheumatoid arthritis, osteoarthritis)
5. Presence or suspicion of neurological involvement (e.g., cervical radiculopathy or cervical myelopathy)
6. Presence or suspicion of serious pathology requiring onwards referral
7. Headache as the primary complaint
8. Unable to understand or follow study procedures with support from family members or professional interpreters

6 ENROLMENT, CONSENT, RANDOMISATION AND BLINDING

6.1 Identification of participants

Potential participants will be identified through two methods.

6.1.1 NHS musculoskeletal waiting lists and new referrals

NHS clinical staff will identify potentially eligible participants from referrals on existing waiting lists and new referrals (GP, specialist, or self-referral).

NHS clinical staff will contact potentially eligible participants via telephone to explain their organisation is participating in the study, provide an initial description of the study procedures and perform an initial telephone screen. The initial telephone screen will identify any exclusion criteria that can be evaluated over the telephone.

Individuals who are interested will be sent a patient Participant Information Leaflet (PIL) and a formal study invitation letter via post or email by NHS clinical staff. The patient PIL will be available in paper format (English), electronic .pdf (English) and electronic video (audio in English or Urdu).

At the time of the initial telephone call, verbal consent will be obtained for a follow-up phone call with NHS clinical staff. The follow-up call provides the opportunity to discuss the study further and to ask questions after receiving and reading the PIL. Potential participants who express an interest in taking part after receiving the PIL and follow-up call will be invited to a face-to-face screen and research appointment to establish eligibility, receive baseline consent, and commence data collection.

If the patient participant is not contactable after 4 weeks or a maximum of 4 contact attempts, they will be considered as not interested and will not be contacted again. They will remain on the physiotherapy waiting list for routine clinical care. The right of an individual to refuse participation without giving reasons will be respected.

6.1.2 NHS musculoskeletal department advertisements

EPIC-Neck study adverts will be placed strategically in NHS musculoskeletal departments. The adverts will include a QR code directing people to a .pdf and video of the patient Participant Information Leaflet. The advert will provide a phone number and email address for people to contact to express an interest in taking part. The contact details will be for an NHS clinical staff member at the local site.

Once an expression of interest is received, the NHS clinical staff member will contact the potential participant via phone to perform an initial telephone screen, provide an opportunity to discuss the study further and to ask questions. The initial telephone screen will identify any exclusion criteria that can be evaluated over the telephone. If an individual was unable to access the PIL electronically, a paper copy will be posted with a follow-up call arranged. If an individual is still interested in taking part, they will be invited to a face-to-face screen and research appointment.

6.2 Face-to-face screen and research appointment

This appointment consists of the final eligible screening, and for those eligible, completion of the consent, data collection and randomisation procedures. The screening and research appointment will be completed by a musculoskeletal physiotherapist (NHS clinical staff) trained in the study procedures a minimum of 24 hours after receiving the PIL.

6.2.1 Screening

Final confirmation of eligibility will be completed at a face-to-face screen. The face-to-face screen involves a short history and physical examination to confirm the diagnosis of chronic non-specific neck pain and determine the presence of any exclusion criteria.

People not meeting the inclusion criteria will not be eligible for the study and routine clinical care will be arranged. People eligible to take part in the study will immediately progress to consent procedures.

6.2.2 Consent

Prior to any study related procedures, written informed consent will be obtained at the baseline research assessment for each participant by the Principal Investigator

(PI). The PI may delegate this responsibility to a local site NHS physiotherapist (hence forth referred to as a “delegate”) documented on the site delegation log, trained in the study procedures and principles of Good Clinical Practice and the Declaration of Helsinki.

The PI or “delegate” will adequately explain the study aims, interventions, potential benefits, and risks. They will explain that participation is voluntary and can withdraw at any time without giving reasons and without prejudicing further treatment. The PIL will be used to facilitate this process.

The potential participant will be given the opportunity to ask any questions before signing and dating the latest version of the Informed Consent Form (ICF). The PI or “delegate” will countersign the ICF. The participant’s study ID number will be entered on the ICF once the participant enters the trial.

A copy of the ICF and PIL will be added to the participants clinical records and the original ICF stored in the Investigator Site File. The patient participant will be provided with a copy of their signed ICF either at the time or via post following the baseline visit. An electronic copy of the ICF will be emailed via secure nhs.net to nhs.net email to the EPIC-Neck study team. A letter will also be sent to the patient participants GP informing them of their involvement in the study.

For those unable to read English, the ICF will be read aloud on their behalf, before signing. For those unable to speak, or understand spoken English, professional interpreters will be provided where possible, and the consent process will be audio recorded. The patient participant will only be provided with a signed physical copy of the ICF and not the audio recording.

Explicit consent will be obtained for a member of the EPIC-Neck research team to audio-record or observe any clinical appointments with the physiotherapist related to the study. The ICF will also ask for explicit consent for the EPIC-Neck study team to contact the participant to be involved in the process evaluation qualitative interviews.

The informed consent process will be recorded in the participants clinical records including date, study name, PIL version number, ICF version number and date of consent.

6.2.3 Baseline questionnaire

Following informed consent, participants will complete the baseline questionnaire and participant contact details form.

6.2.4 Randomisation

Following completion of the baseline questionnaire and participant contact details form, participants will be individually randomised.

Randomisation will be performed by the PI or “delegate” using an online randomisation service (https://www.sealedenvelope.com/). Those delegated with the role of randomising participants will be detailed on the Delegation Log.

Anyone using the online randomisation service will be given a trial password to complete randomisation. All questions and data items on the online registration form must be answered before a Study ID number can be given.

Following randomisation, a confirmatory email will be sent to the Chief Investigator (CI), PI and person completing randomisation if different to the PI. The local NHS site will record the Study ID number on the EPIC-Neck Participant Screening and Recruitment log.

Participants will be individually randomised in a 2:1 ratio, to either 1) Intervention arm (EPIC-Neck) or 2) Usual exercise care. The participant will be informed which group they have been allocated to.

6.2.5 Blinding

As the aim of the feasibility study is to evaluate the acceptability and feasibility of the EPIC-Neck programme and a future definitive trial, participant and data collector blinding is not warranted.

6.3 Participant screening and recruitment log

NHS clinical staff completing the identification, screening and research appointment procedures will record the details of all individuals approached about the study on the EPIC-Neck Participant Screening and Recruitment log. The log will be stored securely by the site according to their local procedures in the Investigator Site File until the end. The log should be available to send to the study team upon request, via nhs.net to nhs.net email.

Details collected will be

- screening number
- local hospital number (redacted before sent to study team)
- age
- gender
- ethnicity
- first language (if available)
- requirement of an interpreter
- indices of multiple deprivation (IMD) decile
- reasons for ineligibility or decline
- date of consent
- treatment group allocation
- study ID number

An individual's postcode will be used to calculate the IMD but the postcode will not be recorded on the screening log. The data collected on the screening log allows evaluation of study uptake and any potential confounding demographic variables.

6.4 Payment

Travel expenses (public transport, parking, mileage) will be provided for the face-to-face screen and research appointment only. Mileage will be covered at 45p per mile.

A £20 voucher will be offered for each patient participant returning the baseline, 3- and 6-month questionnaires.

6.5 Physiotherapist process evaluation consent procedures

6.5.1 Physiotherapists delivering the EPIC-Neck intervention

It is estimated that 8-10 physiotherapists will be required to deliver the EPIC-Neck intervention, providing 3-4 patient participants per physiotherapists. This gives the physiotherapist adequate opportunity to deliver the intervention for process evaluation. Selection of physiotherapists will be purposive to give adequate cover across clinic locations and appointments availability throughout the week at each site.

The PI at the local site will approach physiotherapists via email to invite them to participant in the study and deliver the EPIC-Neck interventions. Physiotherapists will be given a study invite letter, a participant information leaflet, and a consent form. The PIL will state their involvement in delivering the EPIC-Neck intervention requires participating in a questionnaire, training feedback, audio-recording of physiotherapy appointments and a one-to-one interview. Physiotherapists will express an interest in taking part by contacting the CI. Written informed consent will be provided using an electronic signature on the consent form or using pen and paper at the EPIC-Neck training. A copy of the consent form will be emailed to the physiotherapist via secure nhs.net email.

A physiotherapist must consent to the process evaluation to be able to deliver the EPIC-Neck programme. The target sample size for physiotherapist EPIC-Neck delivery process evaluation is 8-10.

6.5.2 Physiotherapists delivering the Usual exercise care intervention

When a participant is allocated to usual exercise care and booked with a physiotherapist, the PI will approach the physiotherapist asking them to participate in the EPIC-Neck study process evaluation (appointment audio-recordings, one-to-one interviews, and questionnaire). They will be provided with a study invite letter, a participant information leaflet, and a consent form. The PIL will state the purpose of audio-recording of physiotherapy appointments, the one-to-one interview and questionnaire.

Physiotherapists will express an interest in taking part by contacting the CI, who will then take written informed consent using an electronic signature on the consent form. A copy of the consent form will be emailed to the physiotherapist via secure nhs.net email. Consent will be verbally confirmed again at the time of the physiotherapy interviews.

A physiotherapist may decline participating the in the process evaluation but are still permitted to deliver Usual exercise care. It is anticipated that a maximum of 15 physiotherapists will be required to deliver Usual exercise care. The minimum target sample size for physiotherapist Usual exercise care process evaluation is n=8 and the maximum is 15.

7 STUDY INTERVENTIONS

Participants will be randomised to either an interventional group (EPIC-Neck programme) or usual exercise care. Both groups will receive any other non-exercise usual care (e.g., work-place advice, postural advice, manual therapy).

7.1 EPIC-Neck programme (Intervention arm)

The EPIC-Neck programme is an evidence-informed, theory driven and co-produced biopsychosocial intervention that supports self-management of chronic non-specific neck pain through a home-based neck exercise programme.

To self-manage symptoms successfully, participants must engage with the exercise programme and the technical components of exercise (exercise type, dosage etc.) must be optimised to maximise the impact on symptoms. The EPIC-Neck programme therefore consists of two main intervention components:

1. A behavioural intervention aiming to maximise participant engagement in their neck exercise self-management programme.

2. A neck exercise intervention optimising exercise prescription to maximise short and long-term benefits.

7.1.1 Development

The EPIC-Neck programme was developed using intervention mapping principles (Figure 1)(27). The behavioural and neck exercise interventions were developed simultaneously.

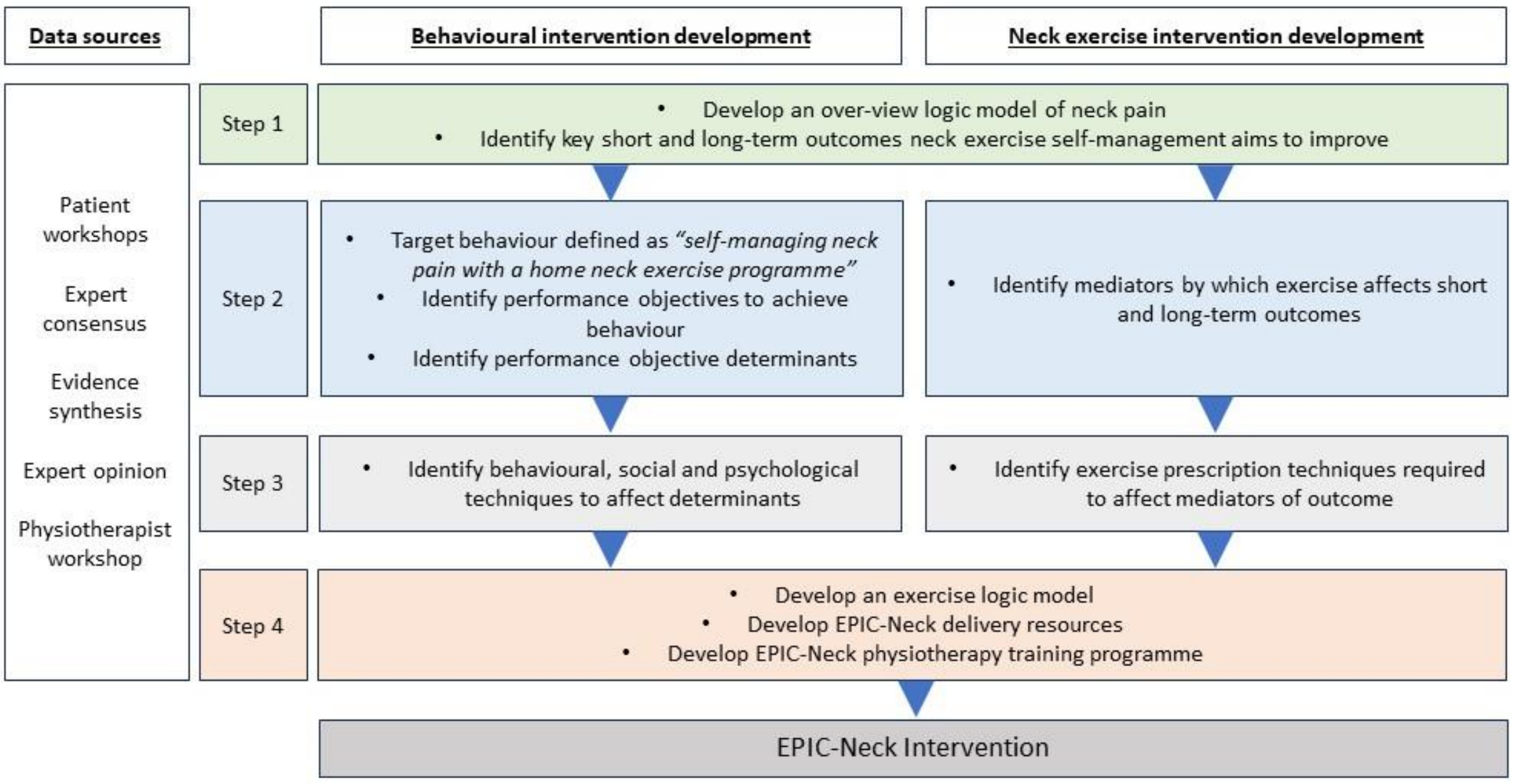

Figure 1 Intervention development flow chart

Step 1 – Evidence was triangulated from the existing literature, clinical guidelines, and patient workshops to develop an overview-level logic model of neck pain,

detailing the key short and long-term impacts of neck pain that an exercise self-management programme aims to improve.

Step 2 – *Behavioural intervention* – The target exercise behaviour was defined as *"self-managing neck pain with a home neck exercise programme"*. This behaviour was broken into smaller performance objectives (what a participant needs to do to successfully achieve the behaviour) using iterative patient workshops, evidence synthesis and expert consensus (35). Four sequential exercise performance objectives were identified:

PO1 – To perform physiotherapist prescribed neck exercise.

PO2 – To self-adjust neck exercise and dosage variables, and independently progress or regress exercise.

PO3 – To self-manage neck pain flare-ups with neck exercise.

PO4 – To engage in a long-term general exercise and understand the benefits and risks of secondary prevention neck exercise.

These performance objectives need to be achieved sequentially to perform the overarching behaviour of self-manging neck pain with exercise i.e., someone must be doing their exercises before learning how to self-adjust exercise dosage. Determinants (barriers or facilitators) for each performance objective were identified (literature, expert opinion, patient workshops) and mapped onto a behavioural intervention matrix.

Exercise intervention - The existing literature was synthesised to identify the direct and indirect mechanistic pathways (mediators) by which exercise interventions affect the impacts identified in Step 1.

Step 3 – *Behavioural intervention* – Behavioural, social and psychological techniques to affect each performance objectives determinants were generated from the theory and techniques tool (36, 37), existing musculoskeletal literature, a taxonomy of behavioural change techniques (38) and patient workshops. The selected techniques were then viewed using the theory of process model of lifestyle behaviour change (39, 40) to assist with describing how the techniques would be applied in practice.

Exercise intervention - The existing literature, and neck pain expert consensus (35) identified the specific exercise prescription and performance techniques needed to affect the outcome mediators.

Step 4 – A logic model of the EPIC-Neck programme was developed detailing the key components of the behavioural and exercise interventions, the proposed mechanisms of action/mediation pathways by which an affect occurs, and the outcomes the intervention intends to improve (Figure 2). The logic model was used to inform the development of delivery resources and physiotherapist training programme.

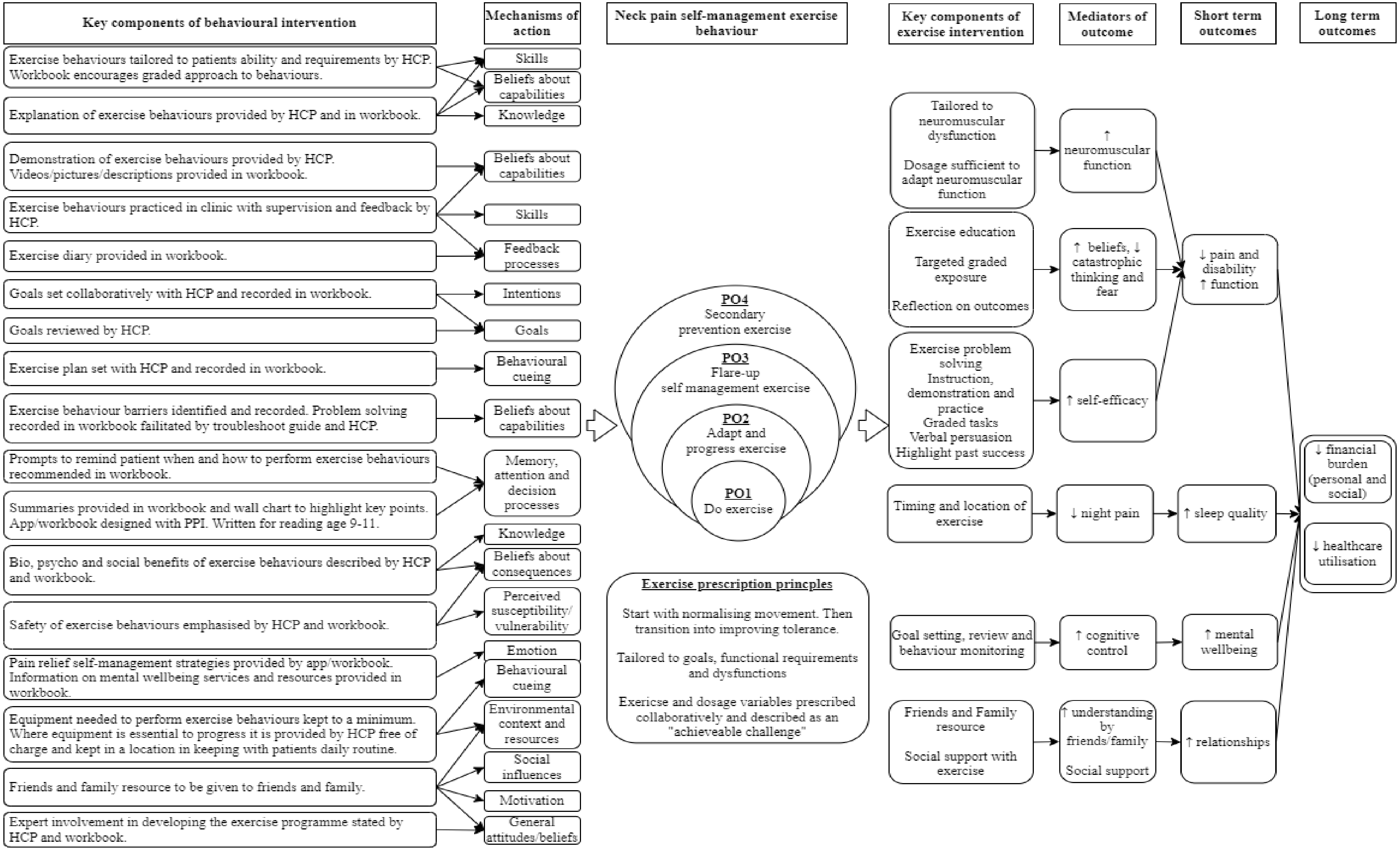

Key components of behavioural intervention
Mechanisms of action
Neck pain self-management exercise behaviour
Key components of exercise intervention
Mediators of outcome
Short term outcomes
Long term outcomes
Exercise behaviours tailored to patients ability and requirements by HCP. Workbook encourages graded approach to behaviours.
Explanation of exercise behaviours provided by HCP and in workbook.
Demonstration of exercise behaviours provided by HCP. Videos/pictures/descriptions provided in workbook.
Exercise behaviours practiced in clinic with supervision and feedback by HCP.
Exercise diary provided in workbook.
Goals set collaboratively with HCP and recorded in workbook.
Goals reviewed by HCP.
Exercise plan set with HCP and recorded in workbook.
Exercise behaviour barriers identified and recorded. Problem solving recorded in workbook failitated by troubleshoot guide and HCP.
Prompts to remind patient when and how to perform exercise behaviours recommended in workbook.
Summaries provided in workbook and wall chart to highlight key points. App/workbook designed with PPI. Written for reading age 9-11.
Bio, psycho and social benefits of exercise behaviours described by HCP and workbook.
Safety of exercise behaviours emphasised by HCP and workbook.
Pain relief self-management strategies provided by app/workbook. Information on mental wellbeing services and resources provided in workbook.
Equipment needed to perform exercise behaviours kept to a minimum. Where equipment is essential to progress it is provided by HCP free of charge and kept in a location in keeping with patients daily routine.
Friends and family resource to be given to friends and family.
Expert involvement in developing the exercise programme stated by HCP and workbook.
Skills
Beliefs about capabilities
Knowledge
Beliefs about capabilities
Skills
Feedback processes
Intentions
Goals
Behavioural cueing
Beliefs about capabilities
Memory, attention and decision processes
Knowledge
Beliefs about consequences
Perceived susceptibility/ vulnerability
Emotion
Behavioural cueing
Environmental context and resources
Social influences
Motivation
General attitudes/beliefs
PO4
Secondary prevention exercise
PO3
Flare-up self management exercise
PO2
Adapt and progress exercise
PO1
Do exercise
Exercise prescription princples
Start with normalising movement. Then transition into improving tolerance.
Tailored to goals, functional requirements and dysfunctions
Exericse and dosage variables prescribed collaboratively and described as an "achieveable challenge"
Tailored to neuromuscular dysfunction
Dosage sufficient to adapt neuromuscular function
Exercise education
Targeted graded exposure
Reflection on outcomes
Exercise problem solving
Instruction, demonstration and practice
Graded tasks
Verbal persuasion
Highlight past success
Timing and location of exercise
Goal setting, review and behaviour monitoring
Friends and Family resource
Social support with exercise
↑ neuromuscular function
↑ beliefs, ↓ catastrophic thinking and fear
↑ self-efficacy
↓ night pain
↑ cognitive control
↑ understanding by friends/family
Social support
↓ pain and disability
↑ function
↑ sleep quality
↑ mental wellbeing
↑ relationships
↓ financial burden (personal and social)
↓ healthcare utilisation

Figure 2 EPIC-Neck logic model

7.1.2 EPIC-Neck programme components

The key components of the behavioural and exercise interventions are described below. Some components appear in both sections as they are intended to influence both the behaviour and neck pain symptoms.

<u>Behavioural intervention</u>

The key EPIC-Neck programme behavioural intervention components are:

Social support – Participants will be provided with a “friends and family” resource to encourage social support in achieving their performance objectives.

Education – Participants will be educated on the bio, psycho, and social benefits of each of the performance objectives and that they are safe to perform.

Instruction, demonstration, practice, and feedback – Participants will be instructed on how to perform performance objectives, provided with demonstrations, have opportunity to practice under supervision and provided with feedback.

Graded and individualised delivery – The delivery and progression between performance objectives will be individualised to participants ability and needs.

Goal setting, action planning, exercise diaries, reviewing and problem-solving – Outcome and behavioural goals will be set collaboratively with action plans. Exercise diaries will be used for self-monitoring. The physiotherapist will review progress with goals and facilitate problem-solving (and refinement of goals) if required.

Prompts – Prompts to remind participants when and how to perform exercise.

Additional self-management resources – Advice and resources for further support with pain relief and mental wellbeing.

Accessibility – Resources were developed with a diverse patient cohort (age, gender, ethnic background, social deprivation), written for a reading age 9-11 and contain simple visual summaries to highlight key points.

<u>Exercise intervention</u>

The key exercise intervention components are:

General exercise prescription principles – Exercises are prescribed in two sequential stages.

Stage 1 – Normalising movement – People with neck pain may experience altered movement patterns of the neck (or surrounding regions) due, but not limited to, poor motor control, fear, or poor self-efficacy. In this stage, active exercise is prescribed to restore normal movement patterns at the neck and surrounding regions.

Stage 2 – Tolerance building – People with neck pain also experience pain or disability when the demands placed upon them are greater than they can tolerate. A person’s tolerance can be affected by, but not limited to, reduced strength,

endurance, or power of the tissues, but also continued fear or self-efficacy limitations with specific functional activities that require increased effort or demand. In this stage, active exercise with increasing demands is prescribed to improve the tolerance of the neck to cope with the individuals' requirements.

Exercise in both stages must always be tailored to ability, goals, functional limitations, and neuromuscular and psychosocial problems, prescribed collaboratively, and considered an "achievable challenge".

Exercise education - Education on the benefits and re-enforcing the safety of exercise.

Target graded exposure – Exercises will be targeted to dysfunctional or fearful movements. Exercise variables (direction, dosage, position etc.) will be graded and guided by participant response and ability.

Reflection on outcomes – Guided reflection on changes in outcomes (pain, range of movement etc.) to reinforce positive responses.

Instruction, demonstration, and practice – Exercise instruction, demonstrations, and supervised practice.

Verbal persuasion and highlight past success –Positive reinforcement and highlighting areas of success to encourage exercise completion.

Exercise timing and location – During exercise action planning participants will consider where and when to complete exercise to reduce any night pain.

Goal setting, review, and monitoring – Collaborative outcome and exercise behavioural goal setting. Exercise diaries for self-monitoring.

Social support – A friends and family resource to encourage social support with neck exercise.

7.1.3 EPIC-Neck programme delivery resources

The EPIC-Neck programme is delivered with supporting resources co-produced by the study team and patient representatives.

EPIC-Neck workbook – A workbook containing sections on general neck pain education, performance objective education and behavioural "FAQs". The workbook also contains activities encouraging goal setting, action planning, problem solving and self-monitoring. The workbook contains an exercise catalogue of frequently prescribed exercises for neck pain.

The workbook guides physiotherapy appointments and is provided to participants to take home. Participants are encouraged to read through and engage with activities within the workbook independently. A paper-based version and mobile application of the workbook are available for participants depending on preference.

EPIC-Neck exercise sheets and wall chart – Exercise sheets to record exercise completion, dosage, and symptoms. An A1 wipeable wall chart is provided as an

alternative to the exercise sheets and includes important education and space to record goals, action plans.

EPIC-Neck friends and family resource – A short booklet for friends and family, providing a summary of neck pain, exercise and how they can support the participant.

7.1.4 EPIC-Neck programme structure and delivery

The EPIC-Neck programme is delivered face-to-face during physiotherapy appointments. The EPIC-Neck programme is structured so that the rate of progression between the sequential performance objectives is individualised. To reflect clinical practice, and the individualised nature of the EPIC-Neck programme there is no maximum number of appointments. Participants will be discharged from care when both the physiotherapist and participant feel they have achieved the behavioural goal of self-managing neck pain with an exercise programme.

Participants are expected to attend at least the initial physiotherapy appointment to be considered to have received the EPIC-Neck programme. Initial physiotherapy appointments and follow-ups will range from 45-60 mins and 20-30 mins respectively.

Physiotherapists will use a patient-centred communication style (e.g., open questions, reflective listening, using an ask-tell-discuss approach) and involve participants in shared decision making throughout all appointments.

Initial appointment – The physiotherapist conducts a routine clinical assessment (history taking and physical examination) evaluating key aspects of the logic model. The EPIC-Neck workbook will be used to introduce performance objective 1 (performing exercise), assist in goal setting and action planning. A home neck exercise programme will be collaboratively prescribed, ensuring exercise dosage goals are an achievable challenge and individualised to ability, limitations, goals, and neuromuscular function. The physiotherapists will encourage the participant to use the EPIC-Neck resources (workbook, exercise sheets, wallchart) for further education, and monitoring of exercise and symptom progression. Utilising social support will be encouraged, and the participant will be directed to the EPIC-Neck friends and family resource.

Follow-up appointments – Appointments begin with a review of the exercise and outcome goals, supplemented by problem solving activities where required. When both the physiotherapist and participant feel performance objective 1 (doing exercise) has been achieved, performance objective 2 (adapting and progressing exercise) will be introduced. The aim of follow-up appointments is to review progress towards a given performance objective and goals and progress onto the next performance objective until the participant can self-manage neck pain with their exercise programme.

7.2 Usual exercise care (Control arm)

The usual exercise care group will receive physiotherapy exercise as per normal practice. As a pragmatic approach to the study design will be adopted, usual exercise care will be determined by the physiotherapist delivering the intervention.

There is significant exercise heterogeneity in neck pain clinical trials (19, 41) and there is no consensus over the exercise that should currently be used in clinical practice. To meet intervention reporting requirements for a definitive study (42, 43) this feasibility study will be used to describe current NHS exercise care for chronic non-specific neck pain. This will be done by triangulating data from audio-recordings, observations of usual exercise care appointments and qualitative one-to-one interviews with physiotherapists delivering usual exercise care (44).

7.3 Training

Physiotherapists delivering usual exercise care interventions will not undergo any training.

Training will be conducted face to face for physiotherapists delivering the EPIC-Neck programme. Training will utilise a mixture of lecture-based learning, mock demonstrations, and behavioural practice (45, 46) covering the key behavioural and exercise interventional components in the logic model and patient-centred communication techniques.

Ongoing support will be provided with 1) a physiotherapist EPIC-Neck training manual 2) booster sessions as required and 3) a point of contact for questions throughout the study within the EPIC-Neck study team and clinical champions based within clinic (45, 47).

Prior to the training, physiotherapists will provide written informed consent for data collected during the training. Physiotherapist demographics (age, gender, qualifications, years' experience, and prior training) will be collected. A Neck Exercise Questionnaire will be used to measure physiotherapists confidence (5-point Likert scale) delivering the EPIC-Neck key behavioural and exercise components within the logic model prior to and after training (46, 48). The questionnaire will be collected using pen and paper. The Neck Exercise Questionnaire will be used to evaluate training fidelity (49, 50). Training satisfaction will also be evaluated with a questionnaire at the end of the training.

8 OUTCOME MEASURES

As no core outcome set exists for chronic non-specific neck pain, the choice of planned definitive study outcome measures was informed by the existing literature and PPIE. Completeness of the proposed outcome measures for the future study will be collected at baseline, and then at 3- and 6-months post-randomisation to evaluate feasibility of data collection.

8.1 Primary outcome measure (for the future trial)

The Neck Disability Index (NDI) - A ten-item self-reported questionnaire assessing how neck pain affects daily life and any disability (51) (domains: pain intensity, personal care, lifting, reading, headaches, concentration, work, driving, sleeping and recreation rated on a 0 to 5 scale). It is the most commonly used neck pain outcome measure in clinical practice and research, with high quality evidence demonstrating good to excellent internal consistency, moderate to excellent test-retest reliability, and a minimum clinically important difference of 5.5 points (52).

8.2 Secondary outcome measures (for the future trial)

- Neck Pain (Visual Analogue Scale)(VAS)(53)
- Health-related quality of life (EQ-5D-5L)(54)
- Mental well-being (Hospital Anxiety and Depression Scale)(55)
- Sleep quality (The Sleep Condition Indicator)(56)
- Specific functional or activity limitations (Patient Specific Functional Scale)(PSFS)(57)
- Social and relationships (Relationships and Self-Worth domains of Assessment of Quality-of-Life (AQoL) 8D)(58, 59)

8.3 Other outcome measures

Other outcome measures will be collected to assist with exploring the mediators of outcome in the logic model.

- Pain beliefs - (Single item Survey of Pain Attitudes Questionnaire)(60)
- Pain related fear – (Tampa Scale of Kinesiophobia-13)(61)
- Pain self-efficacy – (Pain self-efficacy questionnaire)(62)
- Pain at night – (VAS)(53)

Measures of acceptability will be collected using Sekhon acceptability of healthcare intervention questionnaire (33, 34). Scores of 4 and above are acceptable, 2 and below are unacceptable and 3 is neutral.

8.4 Fidelity

Fidelity will be assessed in relation to the key behavioural and exercise components within the logic model (Table 3).

Table 3 Fidelity assessment of key behavioural and exercise intervention components

Intervention Component	Behavioural or exercise component	Delivery fidelity		Receipt fidelity		Enactment fidelity
		Appointment audio-recordings	Direct observations	Participant questionnaire	EPIC-Neck participant interviews	EPIC-Neck participant interviews
Performance Objective 1 – Doing exercise	Both	X	-	X	X	X
Performance Objective 2 – Adapting and progressing exercise	Both	X	-	X	X	X
Performance Objective 3 – Flare-up self-management exercise	Both	X	-	X	X	X
Performance Objective 4 – Secondary prevention exercise	Both	X	-	X	X	X
Behaviours tailored to patients' ability and requirements by HCP. App/workbook encourages graded approach to behaviours	Both	X	X	-	-	-
Explanation of behaviours provided by HCP and in app/workbook	Both	X		-	-	-
Demonstration of behaviours provided by HCP. Videos/pictures provided in app/workbook	Both	X	X	-	-	-
Exercise behaviours practiced in clinic with supervision and feedback by HCP	Both	X	X	-	-	-
Exercise diary provided in app/workbook	Behavioural	X	-	-	-	X
Goals set collaboratively with HCP and recorded in app/workbook	Both	X	-	-	-	-
Goals reviewed by HCP	Both	X	-	-	-	-
Exercise plan set with HCP and recorded in app/workbook	Both	X	-	-	-	-
Workbook and HCP used to identify and record behavioural barriers and problem solving	Both	X	-	X	-	-
Prompts to remind patient when and how to perform behaviours recommended in app/workbook	Behavioural	-	-	-	-	X
Summaries provided in workbook to highlight key points. Workbook designed with PPIE and written for reading age 9-11	Behavioural	-	-	-	-	-
Bio, psycho, and social impacts of behaviours described by HCP and app/workbook	Both	X	-	-	X	-

Intervention Component	Behavioural or exercise component	Delivery fidelity		Receipt fidelity		Enactment fidelity
		Appointment audio-recordings	Direct observations	Participant questionnaire	EPIC-Neck participant interviews	EPIC-Neck participant interviews
Safety of behaviours emphasised by HCP and app/workbook	Both	X	-	X	-	-
Pain relief self-management strategies provided by app/workbook	Behavioural	-	-	X	-	-
Mental wellbeing services and resources identified in app/workbook	Behavioural	-	-	X	-	-
Equipment needed to perform behaviour kept to a minimum. Where equipment is essential to progress, it is provided by HCP free of charge and kept in a location in keeping with patient's daily routine	Behavioural	X	-	-	-	-
Friends and family resource provided	Both	X	-	-	-	X
Expert involvement in developing the exercise programme stated by HCP and app/workbook	Behavioural	-	-	-	-	-
Exercise begins with normalising movement then progress to tolerance optimisation	Exercise	X	-	-	-	-
Exercise tailored to goals, functional requirements, and dysfunction	Exercise	X	X	-	-	-
Exercise dosage an achievable challenge	Exercise	X	X	-	-	-
Graded exposure techniques used	Exercise	X	X	-	-	-
HCP guides reflection on outcomes after exercise	Exercise	X	-	-	-	-
HCP uses verbal persuasion techniques	Exercise	X	-	-	-	-
HCP highlights past success	Exercise	X	-	-	-	-
The timing and location of exercise considering when planning exercise	Exercise	X	-	-	-	-
Abbreviations: HCP – Healthcare Professional; PPIE – Patient and Public Involvement and Engagement						

8.4.1 Fidelity of delivery

Fidelity of delivery refers to the intervention being delivered as intended. All EPIC-Neck appointments will be audio-recorded and coded against a checklist for each behavioural and exercise active ingredient (63, 64) and patient-centred communication techniques. Each item will be scored using Dreyfus criteria (0-6), with pre-defined anchor points of 3 representing acceptable delivery and 6 perfect delivery (65). Initially, two auditors will independently code 3-4 EPIC-Neck appointments and then discuss the coding. This process will be repeated two to three times to ensure a reasonable consensus is achieved (defined as at least 75% of scores being within one point of each other), at which point the remaining appointment recordings will be coded by a single auditor only. A single appointment for each EPIC-Neck physiotherapist will be observed in person by the CI to monitor delivery fidelity for any active ingredients that are not detectable from audio-recordings (e.g., tailoring to neuromuscular dysfunctions; correct demonstration of exercise)(66, 67).

Study monitoring data (total appointments, total did not attend appointments, frequency of appointments) will also be collected.

8.4.2 Fidelity of receipt

Fidelity of receipt refers to the participants understanding of what is being asked of them, and whether they have the necessary skills and ability to perform the interventional component (49). Fidelity of receipt will be evaluated through a participant questionnaire asking them to rate their confidence (1-5 Likert scale) in completing the performance objectives, problem solving, accessing mental wellbeing resources/services and using self-management pain relief strategies (68, 69). The Pain Attitudes Questionnaire (60) and the Tampa Scale of Kinesiophobia-13 (61) will be used to evaluate a participants understanding of the safety of exercise. Participants in both treatment groups will receive the same questionnaire at 3- and 6-months follow-up.

Understanding of completing the performance objectives and the bio, psycho and social benefits of exercise will be evaluated qualitatively using one-to-one semi-structured interviews with EPIC-Neck participants at 4-months follow-up (69).

8.4.3 Fidelity of enactment

Fidelity of enactment refers to whether a participant actually adopts and uses the any key active ingredients of the intervention (49). One-to-one semi-structured interviews with EPIC-Neck participants will be used to evaluate the use, barriers and facilitators of the intervention performance objectives (i.e., doing exercise, changing dosage, self-management of flare ups, secondary prevention exercise), exercise sheets, prompts and friends and family resources (69).

8.5 Contamination

Intervention contamination reduces the contrast between treatments potentially reducing the magnitude of effect (41). Contamination can occur for multiple reasons such as control staff delivering active interventions, participants switching clinicians

or when interventions have similar programme theory/the active ingredients of an intervention already exist to some extent within usual care (sometimes called "background noise")(70).

Strategies included in this study to reduce contamination are that physiotherapists will deliver **either** EPIC-Neck or usual exercise care, only physiotherapists delivering EPIC-Neck will be trained in the intervention and logic model and physiotherapists delivering the EPIC-Neck programme are asked not to share details of the intervention with colleagues.

As the definition of usual exercise care is unknown, it is unclear what key EPIC-Neck active behavioural and exercise components also exist in routine clinical practice and this will be assessed in this feasibility study (71, 72). Usual exercise care appointments will be audio-recorded and coded using the same checklist used for fidelity of delivery assessment. Intervention content will then be compared between the two treatment groups.

To evaluate any reasons for contamination qualitative interviews with physiotherapists delivering usual exercise care will be used. Physiotherapists delivering usual care will also complete the Neck Exercise Questionnaire (evaluating confidence in delivering key components of EPIC-Neck programme) prior to interviews.

8.6 Qualitative interviews

8.6.1 Participant

Qualitative one-to-one semi structured interviews will be conducted with EPIC-Neck participants (n=12-15) at 4-months post randomisation to evaluate EPIC-Neck acceptability, fidelity (receipt & enactment), and to suggest refinements to the intervention and the study procedures.

All participants will be provided with an explanation of the interviews during the consent process and PIL for the feasibility study. Those willing to be interviewed will provide permission to be contacted by the study team, and consent to participate in the interviews, on the feasibility study consent form. It will be made clear, that although they are willing, not all participants will be required for the interviews. If a participant decline's taking part in the interview, they are still eligible to take part in the feasibility study.

Following the 3-month follow-up questionnaire, patient participants who were allocated to the EPIC-Neck programme and who agreed to be contacted will be purposively sampled by the study team to ensure diversity in ethnicity, deprivation, baseline neck pain disability and confidence in completing the EPIC-Neck performance objectives (as rated on the 3-month follow-up questionnaire). Attempts will be made to (briefly) interview those who do not return questionnaires at 3-months to evaluate the reasons underlying any loss to follow-up.

The study team will contact the sampled patient participants via telephone, reminding them of the interview, inviting them to take part and providing opportunity to ask any questions. For those willing to be interviewed, a convenient time and date will be arranged for an interview. Informed consent will be obtained on the feasibility

study consent form at the time of enrolling onto the feasibility study. Interviews will take place using a mode that is preferred for the participant (phone, video, face-to-face). Professional interpreters will be provided for those that require one. Interviews will be semi-structured and a maximum of 90 minutes. Interviews will be audio-recorded using a password-protected dictaphone and transcribed verbatim, removing any personal identifiers during the transcription process.

8.6.2 Physiotherapist

Semi-structured one-to-one interviews will be completed with the physiotherapists who consent into the process evaluation once most of the treatment has been completed.

Interviews with physiotherapist delivering the EPIC-Neck programme will be conducted to explore the training in more depth, evaluate acceptability of delivering the EPIC-Neck programme and assist with refining the intervention.

Interviews with physiotherapist delivering usual exercise care will be conducted to further define usual exercise care and to evaluate reasons for contamination. Physiotherapists delivering usual exercise care will be asked to complete the Neck Exercise Questionnaire (including demographic questions) prior to the interview.

Interviews will take place using a mode that is preferred for the physiotherapist (phone or video). Interviews will be semi-structured and a maximum of 90 minutes. Interviews will be audio-recorded using a password-protected dictaphone and transcribed verbatim, removing any personal identifiers during the transcription process. The rights of physiotherapists to withdraw from interviews will be respected.

9 STUDY PROCEDURES

9.1 Schedule of procedures

Table 4 Schedule of procedures

Procedures	EPIC-Neck training	Screening	Baseline	Treatment	3-months post randomisation	4-months post randomisation	6-months post randomisation	Treatment completion
EPIC-Neck physiotherapist informed consent	X							
EPIC-Neck physiotherapist Neck Exercise Questionnaire	X							
EPIC-Neck physiotherapy training evaluation	X							
Patient Invitation		X						
Patient Eligibility assessment		X	X					
Patient Informed consent			X					
Patient Randomisation			X					
Patient Participant Baseline questionnaire			X					
Usual exercise care physiotherapist informed consent				X				
Intervention				X				
Intervention audio recordings				X				

Intervention observations				X				
Patient Participant Follow-up questionnaire					X		X	
EPIC-Neck participant interviews						X		
Physiotherapist interviews								X
Usual exercise care physiotherapist Neck Exercise Questionnaire								X

9.2 Baseline data collection

9.2.1 Participant

Baseline data (Table 5) will be collected on paper at a face-to-face research assessment, after informed consent procedures.

Table 5 Baseline data collection

**Domain**	**Tool**
Demographics	Age, gender, ethnic background, IMD decile (via postcode)
**Definitive Trial Outcome Measures**	
Pain	VAS (53)
Neck Specific Disability	NDI (73)
Quality of Life	EQ-5D-5L (74)
Mental well-being	Hospital Anxiety and Depression Scale (55)
Relationships	Relationships and Self-Worth domains of AQoL 8D (58, 59)
Sleep	The Sleep Condition Indicator (56)
Specific Functions/Activities	PSFS (57)
**Mediation Measures**	
Night pain	VAS at night (53)
Pain beliefs	Single item Survey of Pain Attitudes Questionnaire (60)
Pain related fear	Tampa Scale of Kinesiophobia-13 (61)
Pain self-efficacy	Pain self-efficacy questionnaire (62)
Abbreviations: Index of multiple deprivation; VAS-Visual analogue scale; NDI-Neck disability index; AQol-Assessment of Quality of Life; PSFS-Patient specific functional scale	

9.2.2 Physiotherapist

Demographics, and confidence delivering key components of the EPIC-Neck intervention will be collected from physiotherapists delivering the EPIC-Neck programme at the training using a Neck Exercise Questionnaire. The questionnaire will be completed using pen and paper.

Demographics, and confidence delivering component key components of the EPIC-Neck intervention will be collected from physiotherapists delivering the Usual exercise care prior to interviews towards the end of the study. The questionnaire will be completed electronically using REDCap.

9.3 Intervention data collection

The data collected during the intervention period are in Table 6.

Table 6 Intervention data collection

**Data**	**Complete by**	**When**
Adverse event case report form	All physiotherapists	As reported at intervention appointments

Appointment audio-recordings	All physiotherapist	All EPIC-NECK and usual care participants appointments
Observation	CI	Single appointment for all physiotherapists
Total appointments, total did not attend appointments, frequency of appointments	PI	Treatment completion
Abbreviations: CI-Chief investigator; PI-Principal investigator		

9.4 Follow-up data collection

9.4.1 Participant

All participants will receive a paper-based questionnaire at 3- and 6- months post randomisation. Questionnaires will be sent via post with a pre-paid envelope to the return to the EPIC-Neck Study Office. A maximum of 2 postal reminders and 1 phone call reminder will be completed where questionnaires are not returned. Questionnaires will be completed via telephone with the CI for participants unable to complete a postal questionnaire. Professional interpreters will be provided for the questionnaire phone call if required. The data collected is in Table 7.

Table 7 Follow-up data collection

Domain	Tool
**Definitive trial outcome measure**	
Pain	VAS (53)
Disability	NDI (73)
Health Related Quality of Life	EQ-5D-5L (74)
Mental well-being	Hospital Anxiety and Depression Scale (55)
Relationships with others	Relationships and Self-Worth domains of AQoL 8D (58, 59)
Sleep	The Sleep Condition Indicator (56)
Specific function and activities	PSFS (57)
**Other data**	
Night pain	VAS at night (53)
Exercise acceptability	Acceptability of healthcare interventions questionnaire (33)
Fidelity of receipt	Confidence in completing the performance objectives, problem solving, accessing mental wellbeing resources/services, and using self-management pain relief strategies (68, 69)
Pain beliefs	Single item Survey of Pain Attitudes Questionnaire (60)
Pain related fear	Tampa Scale of Kinesiophobia-13 (61)
Pain self-efficacy	Pain self-efficacy questionnaire (62)
Abbreviations: VAS-Visual analogue scale; NDI-Neck disability index; AQol-Assessment of Quality of Life; PSFS-Patient specific functional scale	

Qualitative interviews will also be completed with participants receiving the EPIC-Neck intervention 4 months post randomisation.

9.4.2 Physiotherapist

The change in confidence of physiotherapists delivering the EPIC-Neck programme will be collected using the Neck Exercise Questionnaire after training has been completed. The questionnaire will be completed using pen and paper.

Qualitative interviews will also be completed with physiotherapists (Table 8).

Table 8 Interview data collection

Who	When
Physiotherapists delivering the EPIC-Neck programme	Once the majority of study interventions are complete
Physiotherapists delivering usual exercise care	Once all study interventions are complete

9.5 Withdrawal criteria

Participants have the right to withdraw at any time during the study or for the interviews. The study team may discontinue a participant from the study at any time if considered necessary.

The changes in levels of participation within the trial are categorised in the following ways:

No study intervention: The participant would no longer like to receive the study intervention but is willing to be followed up in accordance with the schedule of procedures.

No further data collection: The participant would no longer like to receive the study intervention AND does not wish for any further data to be collected (i.e., only data collected prior to any changes of levels in participation can be used in the trial analysis).

Withdrawal, and the level of, will be documented using the withdrawal CRF and reported to the study team.

10 SAFETY REPORTING

10.1 Definitions

The terms used within safety reporting are informed by the “NIHR Decision Tree for Adverse Event Reporting – Non CTIMPS” (75). Definitions and decision making are in Table 9. A “medical occurrence” is any problem a participant experiences that interferes with function, lasting >2 days and/or requiring medication or seeking health professional support (76).

The following criteria will be used to define serious: results in death; is life-threatening; requires hospitalisation or prolongation of hospitalisation; results in persistent or significant disability or incapacity; consists of a congenital abnormality or birth defect.

Table 9 Adverse event reporting

Term	Definition
**Adverse Event (AE)**	Any **non-serious** untoward medical occurrence which a participant experiences to whom an intervention (experimental or control) has been administered, that is **not caused by or related to the intervention**.
**Related Event**	Any **non-serious** untoward medical occurrence which a participant experiences to whom an intervention (experimental or control) has been administered, **that is, or possibly caused by or related to the intervention.** e.g., temporary increases in neck pain symptoms, delayed onset muscle soreness
**Serious Adverse Event (SAE)**	Any **serious** untoward medical occurrence which a participant experiences to whom an intervention (experimental or control) has been administered, that is **not caused by or related to the intervention**.
**Serious Related Event**	Any **serious** untoward medical occurrence which a participant experiences to whom an intervention (experimental or control) has been administered, **that is, or possibly caused by or related to the intervention and is consistent with the available information (i.e., expected).**
**Unexpected Serious Related Event**	Any **serious** untoward medical occurrence which a participant experiences to whom an intervention (experimental or control) has been administered, **that is, or possibly caused by or related to the intervention and unexpected.**

10.2 Safety recording and reporting

All adverse events described in Table 9 will be recorded using the Study Adverse Event (AE) form from randomisation up until the 6-month follow-up and evaluated as part of process evaluation. The Study AE form will include a description of the medical occurrence, date of onset, end date, severity, and assessment of relatedness to study interventions. A copy of the Study AE form will be emailed via secure nhs.net email to the EPIC-Neck study team, and the original retained in the site file.

When a local site becomes aware of any SAE, the local PI will review the SAE and decide on causality to the intervention and expectedness. The local site will notify the EPIC-NECK study team via email or telephone within 24 hours of the PI becoming aware of any Unexpected Serious Related Events.

Once received, causality and expectedness will be confirmed by the CI or delegate (Nominated Person). In the event that consensus is not reached between the PI and Nominated Person about assessment of causality and expectedness, this will be escalated to the CI for further discussion. However, if no consensus decision is reached about expectedness after further discussion within one working day, and the

SAE is judged to be unexpected by any one of either the PI, Nominated Person or CI, the event will be classified as an Unexpected Serious Related Event.

Unexpected Serious Related Event's will be reported to the Research Ethics Committee (REC) that gave a favourable opinion of the study within 15 working days of the CI becoming aware of the event, using the HRA report of serious adverse events form (HRA website). All Unexpected Serious Related Events will be reported to the Study Management Group at their next meeting.

11 DATA HANDLING AND RECORD KEEPING

11.1 Completed questionnaires

Baseline participant questionnaires will be completed in clinic on paper. The site PI or delegated staff (as directed by the delegation log) will oversee the completion and check for any missing data. Originals will be stored in the local Site File and scanned copies will be send to the EPIC-Neck Study Office via nhs.net to nhs.net email.

Follow-up participant questionnaires will be completed at the participants home on paper and returned to the study office via pre-paid envelope. Where participants need support with completing questionnaires due to literacy ability, participant questionnaires will be completed over the phone by the Study Office and recorded on paper.

EPIC-Neck physiotherapist questionnaires will be completed via pen and paper. Paper copies will scanned and stored electronically by the Study Office. Original paper copies will be destroyed.

Usual exercise care physiotherapist questionnaires will be completed electronically using REDCap.

11.2 Data management, protection, and confidentiality

All investigators and study site staff must comply with the requirements of the Data Protection Act 2018 with regards to the collection, storage, processing, and disclosure of personal information and will uphold the Act's core principles. A data protection impact assessment is being undertaken to ensure the EPIC-Neck mobile application workbook is compliant with General Data Protection Regulation. The app developers are registered on the NHS Data Security and Protection Toolkit.

Sites will store any data in the local Site Files in accordance with their local information governance policies.

All study specific documentation will refer to the participant with a unique study participant number, other than the consent form and follow-up contact details.

Case Report Form's (CRF) and baseline participant questionnaires received via email by the EPIC-Neck Study Office at Birmingham Community Healthcare NHS Foundation Trust will be stored on the Trust's local shared drive, with access restricted to the Study team.

Participant questionnaires received by the EPIC-Neck Study Office via post, or completed on paper via phone call will be stored in a locked filing cabinet in accordance with BCHC's information governance policy. Documents will be then scanned and stored on the Trust's local shared drive, with access restricted to the Study team. Once scanned, original paper copies will be destroyed.

CRF's and participant questionnaire data will be transcribed by a member of the office team onto the EPIC-Neck database, an excel spreadsheet stored on the Trust's local shared drive, with access restricted to the Study team.

Any missing or ambiguous data on CRF's identified by the Study Office will be queried with the site PI or person with delegated responsibility. Missing or ambiguous data on participant questionnaires will be queried with the participant.

Contact details will be stored separately from outcome data and deleted when they are no longer required as part of the study (within 12 months after last data collection point).

Password protected dictaphones will be used for all audio-recordings. Participant appointment audio-recordings will be collected by the Study Office by attending the clinic and transferring the files onto a BCHC laptop. One-to-one interview audio-recordings with participants and physiotherapist will be completed by the CI and immediately transferred to a BCHC laptop. Once files have been transferred to the BCHC laptop, they will be stored on the Trust's local shared drive, with access restricted to the Study team at Birmingham Community Healthcare NHS Foundation Trust. Once transferred, files on the dictaphone will be deleted.

The second auditor performing fidelity assessments of appointment audio-recordings will be a member of the EPIC-Neck study team and employee of Birmingham Community Healthcare NHS Foundation Trust and will therefore have access to the original audio-recordings.

Audio recordings maybe transcribed by a commercial company who will have a confidentiality agreement in place with Birmingham Community Healthcare NHS Foundation Trust. The commercial company will remove and destroy all personal identifiable data from the transcripts and participants will be coded and referred to in study documents using a unique identification number. The audio recordings will be kept by Birmingham Community Healthcare NHS Foundation Trust.

Anonymised data may be stored on the CI's personal password protected laptop to allow access to analysis software not available on Trust devices (SPSS, NVIVO).

11.3 Archiving

It is the responsibility of the site PI to ensure all essential study documentation and source documents at their site are securely retained for the contractual period. Identifiable and anonymised data will be archived by BCHC for a maximum of 25 years.

12 MONITORING, AUDIT, AND INSPECTION

Quality assurance checks will be completed by the CI including a single inspection of the Study Master File at each site, observation of one research assessment and fidelity assessments.

A Study Management Group consisting of methodological and subject expertise and patient representation will be responsible for the overall running of the study. Day to day study management will be the responsibility of the CI. The CI will be responsible for the training conducted at research sites. As this is a low-risk feasibility study there will be no Study Steering or Data and Safety Monitoring Committee.

13 END OF STUDY DEFINITION

The end of the study will be the date of the last data capture including resolution of data clarifications. This will allow sufficient time for the completion of protocol procedures, data collection and input and data cleaning. The Study Office will notify the REC and the Sponsor within 90 days of the end of the study. Where the study has terminated early, the Study Office will notify the REC within 15 days of the end of trial. The Study Office will provide the REC and the Sponsor with a summary of the study report within 12 months of the end of the study.

14 STATISTICS AND DATA ANALYSIS

14.1 Sample size calculation

A sample size has been calculated to provide estimates (within specified confidence intervals) of retention rates. A sample size of 45 would allow an estimation of retention rate of 80% (77) with a margin of error ± 12% and confidence level 95%.

14.2 Statistical analysis plan

Quantitative data will be analysed and summarised to evaluate uptake rate, retention, outcomes completion rates, intervention acceptability, adherence, and fidelity. Descriptive statistics (means, standard deviations, frequencies, or proportions, as appropriate) will be used. As this is a feasibility study no formal between-group analysis will be conducted (78).

Audio-recording data for intervention fidelity will be analysed independently by two raters (lead applicant and one independent rater with experience in coding behavioural interventions). Initially, x2 auditors will code all EPIC-Neck appointments until consensus is achieved, at which point the remaining appointments will be coded by a single auditor only. Summarised fidelity data will be reported using descriptive statistics (mean, SD) and collated by the auditor and by checklist item.

Qualitative data from one-to-one interviews will be transcribed verbatim and analysed by the lead applicant via inductive thematic analysis and deductively against the constructs of the healthcare intervention acceptability theoretical framework (79). Quantitative and qualitative data will be analysed using IBM SPSS Statistics Version 25 and QSR International’s NVivo 12 Plus software respectively.

15 STUDY ORGANISATIONAL STRUCTURE

15.1 Sponsor

The sponsor for this study is Birmingham Community Healthcare NHS Foundation Trust.

15.2 Study management group

The Study Management Group comprises individuals responsible for the day-to-day management of the trial. The role of the group is to monitor all aspects of the conduct and progress of the trial, ensure that the protocol is adhered to and take appropriate action to safeguard participants and the quality of the trial itself. The SMG will meet sufficiently frequently to fulfil its function.

16 ETHICAL CONSIDERATIONS

The study will be conducted in accordance with the UK Policy Framework for Health and Social Care Research and applicable UK Acts of Parliament and Statutory Instruments (and relevant subsequent amendments), and the Principles of Good Clinical Practice as set out in the UK Statutory Instrument (2004/1031; and subsequent amendments). The protocol will be submitted to and approved by the REC prior to the start of the trial.

Before any participants are enrolled into the trial, the PI at each site is required to obtain the necessary local approval.

It is the responsibility of the PI to ensure that all subsequent amendments gain the necessary local approval. This does not affect the individual clinicians' responsibility to take immediate action if thought necessary to protect the health and interest of individual participants.

17 PUBLIC AND PATIENT INVOLVEMENT AND ENGAGEMENT

A PPIE group consisting of 20 diverse members assisted with co-design and production of the EPIC-Neck programme and in the development of this protocol and supporting patient information leaflet. Key involvement was 1) identifying important outcome measures 2) developing study procedures to support inclusivity and 3) pilot testing CRF's and questionnaires. During the feasibility study 2-3 PPIE representatives will sit on the Study Management Group. The wider PPIE group (n=20) will be involved in interpretation of study findings, evaluating progression criteria, and developing public facing dissemination material. PPIE will be reported using the GRIPP2 checklist in the final publication (80).

18 FINANCIAL AND OTHER COMPETING INTERESTS

XXXXX, Clinical Doctoral Research Fellow, NIHRXXXX is funded by HEE/NIHR for this research project. There are no other competing interests to declare.

19 INSURANCE AND INDEMNITY

The sites included in this study will be covered by the NHS indemnity scheme to meet the potential legal liability of the sponsor(s) for harm to participants arising from the management of the research, design of the research and conduct of the research.

The collaborators from outside the NHS will be responsible for their own indemnity insurance arrangements. This is specified in the contract with BCHC.

20 POST-STUDY CARE

At the end of the study follow-up period, the participant's continued care will be decided by the clinical team at their GP or musculoskeletal service.

Participants randomised to EPIC-Neck will continue to have access to the paper EPIC-Neck intervention material indefinitely. EPIC-Neck participants will have access to the EPIC-Neck app up until 2 years after the start of the study.

21 ACCESS TO FINAL STUDY DATA SET

The final data set will only be available to the EPIC-Neck study team. Formal requests for access should be made to the Study Management Group who will review the requests on an individual basis.

22 PUBLICATION PLAN

The EPIC-Neck study protocol will be published in an appropriate journal. Outputs from the study will be disseminated via academic publication, conference presentation and BCHC social media.

No professional writers will be used to prepare manuscripts for publication. The criteria for individually named authors or group authorship will follow the guidance of The International Committee of Medical Journal Editors.

The study participants and PPIE representatives will be informed of the findings via lay summary provided by post or email to the participants. Some PPIE representatives and participants may be involved in producing a video and/or attending conferences to disseminate the findings.

23 REFERENCES

1. James SL, Abate D, Abate KH, Abay SM, Abbafati C, Abbasi N, et al. Global, regional, and national incidence, prevalence, and years lived with disability for 354 diseases and injuries for 195 countries and territories, 1990–2017: a systematic analysis for the Global Burden of Disease Study 2017. Lancet. 2018;392(10159):1789-858.

2. Yang H, Haldeman S. Chronic Spinal Pain and Financial Worries in the US Adult Population. Spine (Phila Pa 1976). 2020;45(8):528-33.

3. Nolet PS, Côté P, Kristman VL, Rezai M, Carroll LJ, Cassidy JD. Is neck pain associated with worse health-related quality of life 6 months later? A population-based cohort study. The Spine Journal. 2015;15(4):675-84.

4. Hallman DM, Holtermann A, Dencker-Larsen S, Birk Jorgensen M, Norregaard Rasmussen CD. Are trajectories of neck-shoulder pain associated with sick leave and work ability in workers? A 1-year prospective study. BMJ Open. 2019;9(3):e022006.

5. Beltran-Alacreu H, López-De-Uralde-Villanueva I, Calvo Lobo C, La-Touche R, Cano De La Cuerda R, Gil-Martínez A, et al. Prediction models of health-related quality of life in different neck pain conditions: a cross-sectional study. Patient Preference and Adherence. 2018;Volume 12:657-66.

6. Hallman DM, Rasmussen CDN, Jorgensen MB, Holtermann A. Time course of neck-shoulder pain among workers: A longitudinal latent class growth analysis. Scand J Work Environ Health. 2018;44(1):47-57.

7. van Randeraad-van der Zee CH, Beurskens AJ, Swinkels RA, Pool JJ, Batterham RW, Osborne RH, et al. The burden of neck pain: its meaning for persons with neck pain and healthcare providers, explored by concept mapping. Qual Life Res. 2016;25(5):1219-25.

8. Bier JD, Scholten-Peeters WGM, Staal JB, Pool J, van Tulder MW, Beekman E, et al. Clinical Practice Guideline for Physical Therapy Assessment and Treatment in Patients With Nonspecific Neck Pain. Phys Ther. 2018;98(3):162-71.

9. Blanpied PR, Gross AR, Elliott JM, Devaney LL, Clewley D, Walton DM, et al. Neck Pain: Revision 2017. J Orthop Sports Phys Ther. 2017;47(7):A1-A83.

10. National Institute for Health and Care Excellence. Chronic pain (primary and secondary) in over 16s: assessment of all chronic pain and mangement of chronic primary pain. NICE Guideline NG193 [Internet]. 2021. Available from: https://www.nice.org.uk/guidance/ng193.

11. Alreni A, McLean S, Demack S, Harrop D, Kilner K. Managing non-specific neck pain: A national survey of current UK physiotherapy practice. Orthopaedic Proceedings. 2018;100-B(SUPP_2):14-.

12. Gross A, Kay TM, Paquin JP, Blanchette S, Lalonde P, Christie T, et al. Exercises for mechanical neck disorders. Cochrane Database Syst Rev. 2015;1:CD004250.

13. Wilhelm MP, Donaldson M, Griswold D, Learman KE, Garcia AN, Learman SM, et al. The Effects of Exercise Dosage on Neck-Related Pain and Disability: A Systematic Review With Meta-analysis. J Orthop Sports Phys Ther. 2020;50(11):607-21.

14. Zebis MK, Andersen CH, Sundstrup E, Pedersen MT, Sjøgaard G, Andersen LL. Time-Wise Change in Neck Pain in Response to Rehabilitation with Specific Resistance Training: Implications for Exercise Prescription. PLoS ONE. 2014;9(4):e93867.

15. de Vos Andersen NB, Kent P, Hjort J, Christiansen DH. Clinical course and prognosis of musculoskeletal pain in patients referred for physiotherapy: does pain site matter? BMC Musculoskelet Disord. 2017;18(1):130.

16. Silva PV, Costa LOP, Maher CG, Kamper SJ, Costa L. The New Agenda for Neck Pain Research: A Modified Delphi Study. The Journal of orthopaedic and sports physical therapy. 2019;49(9):666-74.

17. Rankin G, Summers R, Cowan K, Barker K, Button K, Carroll SP, et al. Identifying Priorities for Physiotherapy Research in the UK: the James Lind Alliance Physiotherapy Priority Setting Partnership. Physiotherapy. 2020;107:161-8.

18. Cieza A, Causey K, Kamenov K, Hanson SW, Chatterji S, Vos T. Global estimates of the need for rehabilitation based on the Global Burden of Disease study 2019: a systematic analysis for the Global Burden of Disease Study 2019. Lancet. 2020;396(10267):2006-17.

19. Price J, Rushton A, Tyros I, Tyros V, Heneghan NR. Effectiveness and optimal dosage of exercise training for chronic non-specific neck pain: A systematic review with a narrative synthesis. PLoS One. 2020;15(6):e0234511.

20. Geneen LJ, Moore RA, Clarke C, Martin D, Colvin LA, Smith BH. Physical activity and exercise for chronic pain in adults: an overview of Cochrane Reviews. Cochrane Database Syst Rev. 2017;4:CD011279.

21. Craig P, Dieppe P, Macintyre S, Michie S, Nazareth I, Petticrew M. Developing and evaluating complex interventions: new guidance. London: Medical Research Council; 2019.

22. de Zoete RM, Armfield NR, McAuley JH, Chen K, Sterling M. Comparative effectiveness of physical exercise interventions for chronic non-specific neck pain: a systematic review with network meta-analysis of 40 randomised controlled trials. Br J Sports Med. 2020.

23. Hayden JA, van Tulder MW, Tomlinson G. Systematic review: strategies for using exercise therapy to improve outcomes in chronic low back pain. Annals of Internal Medicine. 2005;142(9):776-86.

24. Polaski AM, Phelps AL, Kostek MC, Szucs KA, Kolber BJ. Exercise-induced hypoalgesia: A meta-analysis of exercise dosing for the treatment of chronic pain. PLoS One. 2019;14(1):e0210418.

25. Jordan JL, Holden MA, Mason EE, Foster NE. Interventions to improve adherence to exercise for chronic musculoskeletal pain in adults. Cochrane Database Syst Rev. 2010(1):CD005956.

26. Meade LB, Bearne LM, Sweeney LH, Alageel SH, Godfrey EL. Behaviour change techniques associated with adherence to prescribed exercise in patients with persistent musculoskeletal pain: Systematic review. Br J Health Psychol. 2019;24(1):10-30.

27. Bartholomew Eldredge LK, Markham CM, Ruiter RAC, Fernández ME, Kok G, Parcel GS. Planning Health Promotion Programs: An Intervention Mapping Approach. Hoboken: Hoboken: John Wiley & Sons, Incorporated; 2016.

28. Thabane L, Lancaster G. A guide to the reporting of protocols of pilot and feasibility trials. Pilot Feasibility Stud. 2019;5(1):37.

29. Chan AW, Tetzlaff JM, Altman DG, Laupacis A, Gotzsche PC, Krleza-Jeric K, et al. SPIRIT 2013 statement: defining standard protocol items for clinical trials. Ann Intern Med. 2013;158(3):200-7.

30. Chan AW, Tetzlaff JM, Gotzsche PC, Altman DG, Mann H, Berlin JA, et al. SPIRIT 2013 explanation and elaboration: guidance for protocols of clinical trials. BMJ. 2013;346(jan08 15):e7586.

31. Eldridge SM, Chan CL, Campbell MJ, Bond CM, Hopewell S, Thabane L, et al. CONSORT 2010 statement: extension to randomised pilot and feasibility trials. BMJ. 2016;355:i5239.

32. Mellor K, Albury C, Dutton SJ, Eldridge S, Hopewell S. Recommendations for progression criteria during external randomised pilot trial design, conduct, analysis and reporting. Pilot Feasibility Stud. 2023;9(1):59.

33. Sekhon M, Cartwright M, Francis JJ. Development of a theory-informed questionnaire to assess the acceptability of healthcare interventions. BMC Health Serv Res. 2022;22(1):279.

34. Sekhon M, Cartwright M, Francis JJ. Acceptability of healthcare interventions: an overview of reviews and development of a theoretical framework. BMC Health Serv Res. 2017;17(1):88.

35. Price J, Rushton A, Tyros V, Heneghan NR. Expert consensus on the important chronic non-specific neck pain motor control and segmental exercise and dosage variables: An international e-Delphi study. PLoS One. 2021;16(7):e0253523.

36. Carey RN, Connell LE, Johnston M, Rothman AJ, de Bruin M, Kelly MP, et al. Behavior Change Techniques and Their Mechanisms of Action: A Synthesis of Links Described in Published Intervention Literature. Ann Behav Med. 2019;53(8):693-707.

37. Johnston M, Carey RN, Connell Bohlen LE, Johnston DW, Rothman AJ, de Bruin M, et al. Development of an online tool for linking behavior change techniques and mechanisms of action based on triangulation of findings from literature synthesis and expert consensus. Transl Behav Med. 2021;11(5):1049-65.

38. Michie S, Richardson M, Johnston M, Abraham C, Francis J, Hardeman W, et al. The behavior change technique taxonomy (v1) of 93 hierarchically clustered techniques: building an international consensus for the reporting of behavior change interventions. Ann Behav Med. 2013;46(1):81-95.

39. Greaves C. Supporting Health Behavior Change in General Practice. In: Barnard KD, Lloyd CE, editors. Psychology and Diabetes Care: A Practical Guide. London: Springer London; 2012. p. 157-70.

40. Greaves CJ, Reddy P, Sheppard KE, editors. Supporting behaviour change for diabetes prevention2010.

41. Gross A, Kay TM, Paquin JP, Blanchette S, Lalonde P, Christie T, et al. Exercises for mechanical neck disorders. Cochrane Database Syst Rev. 2015;1(1):CD004250.

42. Hoffmann TC, Glasziou PP, Boutron I, Milne R, Perera R, Moher D, et al. Better reporting of interventions: template for intervention description and replication (TIDieR) checklist and guide. BMJ. 2014;348(mar07 3):g1687.

43. Slade SC, Dionne CE, Underwood M, Buchbinder R, Beck B, Bennell K, et al. Consensus on Exercise Reporting Template (CERT): Modified Delphi Study. Phys Ther. 2016;96(10):1514-24.

44. Yorganci E, Evans CJ, Johnson H, Barclay S, Murtagh FE, Yi D, et al. Understanding usual care in randomised controlled trials of complex interventions: A multi-method approach. Palliat Med. 2020;34(5):667-79.

45. To D, De Carvalho D, Pike A, Lawrence R, Etchegary H, Patey AM, et al. A qualitative study exploring perceived barriers and enablers to fidelity of training and delivery for an intervention to reduce non-indicated imaging for low back pain. Chiropr Man Therap. 2023;31(1):6.

46. Beneciuk JM, George SZ, Greco CM, Schneider MJ, Wegener ST, Saper RB, et al. Targeted interventions to prevent transitioning from acute to chronic low back pain in high-risk patients: development and delivery of a pragmatic training course of psychologically informed physical therapy for the TARGET trial. Trials. 2019;20(1):256.

47. Craven K, Holmes J, Powers K, Clarke S, Cripps RL, Lindley R, et al. Embedding mentoring to support trial processes and implementation fidelity in a randomised controlled trial of vocational rehabilitation for stroke survivors. BMC Med Res Methodol. 2021;21(1):203.

48. Chlan LL, Ridgeway JL, Tofthagen CS, Hamann BR, Mele KE, Dozois D, et al. Iterative development and pilot testing of an intervention fidelity monitoring plan for the enhanced, electronic health record-facilitated pragmatic clinical trial: Implications for training and protocol integrity. Contemp Clin Trials Commun. 2021;24:100868.

49. Borrelli B. The Assessment, Monitoring, and Enhancement of Treatment Fidelity In Public Health Clinical Trials. J Public Health Dent. 2011;71(s1):S52-S63.

50. Hawkes RE, Cameron E, Miles LM, French DP. The Fidelity of Training in Behaviour Change Techniques to Intervention Design in a National Diabetes Prevention Programme. Int J Behav Med. 2021;28(6):671-82.

51. Vernon H, Mior S. The Neck Disability Index: a study of reliability and validity. J Manipulative Physiol Ther. 1991;14(7):409-15.

52. Bobos P, MacDermid JC, Walton DM, Gross A, Santaguida PL. Patient-Reported Outcome Measures Used for Neck Disorders: An Overview of Systematic Reviews. J Orthop Sports Phys Ther. 2018;48(10):775-88.

53. Modarresi S, Lukacs MJ, Ghodrati M, Salim S, MacDermid JC, Walton DM, et al. A Systematic Review and Synthesis of Psychometric Properties of the Numeric Pain Rating Scale and the Visual Analog Scale for Use in People With Neck Pain. Clin J Pain. 2021;38(2):132-48.

54. Feng YS, Kohlmann T, Janssen MF, Buchholz I. Psychometric properties of the EQ-5D-5L: a systematic review of the literature. Qual Life Res. 2021;30(3):647-73.

55. Zigmond AS, Snaith RP. The hospital anxiety and depression scale. Acta Psychiatr Scand. 1983;67(6):361-70.

56. Espie CA, Kyle SD, Hames P, Gardani M, Fleming L, Cape J. The Sleep Condition Indicator: a clinical screening tool to evaluate insomnia disorder. BMJ Open. 2014;4(3):e004183.

57. Stratford P, Gill C, Westaway M, Binkley J. Assessing disability and change on individual patients: a report of a patient specific measure. Physiotherapy canada. 1995;47(4):258-63.

58. Maxwell A, Ozmen M, Iezzi A, Richardson J. Deriving population norms for the AQoL-6D and AQoL-8D multi-attribute utility instruments from web-based data. Qual Life Res. 2016;25(12):3209-19.

59. Richardson J, Iezzi A, Khan MA, Maxwell A. Validity and reliability of the Assessment of Quality of Life (AQoL)-8D multi-attribute utility instrument. Patient. 2014;7(1):85-96.

60. Jensen MP, Keefe FJ, Lefebvre JC, Romano JM, Turner JA. One- and two-item measures of pain beliefs and coping strategies. Pain. 2003;104(3):453-69.

61. Dupuis F, Cherif A, Batcho C, Massé-Alarie H, Roy JS. The Tampa Scale of Kinesiophobia: A Systematic Review of Its Psychometric Properties in People With Musculoskeletal Pain. Clin J Pain. 2023;39(5):236-47.

62. Nicholas N. Paper presented at the Annual Conference of the British Psychological Society. 1989.

63. Frost J, Wingham J, Britten N, Greaves C, Abraham C, Warren FC, et al. Home-based rehabilitation for heart failure with reduced ejection fraction: mixed methods process evaluation of the REACH-HF multicentre randomised controlled trial. BMJ Open. 2019;9(8):e026039.

64. Thompson TP, Lambert JD, Greaves CJ, Taylor AH. Intervention delivery fidelity assessment of a counseling-based intervention for promoting smoking reduction and increasing physical activity. Health Psychol. 2018;37(7):627-37.

65. Dreyfus SE. The Five-Stage Model of Adult Skill Acquisition. Bulletin of Science, Technology & Society. 2016;24(3):177-81.

66. Orton E, Lafond N, Skelton DA, Coupland C, Gladman JRF, Iliffe S, et al. Implementation fidelity of the Falls Management Exercise Programme: a mixed methods analysis using a conceptual framework for implementation fidelity. Public Health. 2021;197:11-8.

67. Rookes TA, Schrag A, Walters K, Armstrong M. Measures of fidelity of delivery and engagement in self-management interventions: A systematic review of measures. Clin Trials. 2022;19(6):665-72.

68. Lambert JD, Greaves CJ, Farrand P, Price L, Haase AM, Taylor AH. Web-Based Intervention Using Behavioral Activation and Physical Activity for Adults With Depression (The eMotion Study): Pilot Randomized Controlled Trial. J Med Internet Res. 2018;20(7):e10112.

69. Green SMC, Hall LH, Rousseau N, French DP, Graham CD, Collinson M, et al. Acceptability, fidelity and trial experience of four intervention components to support medication adherence in women with breast cancer: A process evaluation protocol for a pilot fractional factorial trial. NIHR Open Research. 2023;3(3).

70. Magill N, Knight R, McCrone P, Ismail K, Landau S. A scoping review of the problems and solutions associated with contamination in trials of complex interventions in mental health. BMC Med Res Methodol. 2019;19(1):4.

71. Luker JA, Craig LE, Bennett L, Ellery F, Langhorne P, Wu O, et al. Implementing a complex rehabilitation intervention in a stroke trial: a qualitative process evaluation of AVERT. BMC Med Res Methodol. 2016;16:52.

72. Robinson K, Allen F, Darby J, Fox C, Gordon AL, Horne JC, et al. Contamination in complex healthcare trials: the falls in care homes (FinCH) study experience. BMC Med Res Methodol. 2020;20(1):46.

73. Vernon H. The Neck Disability Index: state-of-the-art, 1991-2008. J Manipulative Physiol Ther. 2008;31(7):491-502.

74. Herdman M, Gudex C, Lloyd A, Janssen M, Kind P, Parkin D, et al. Development and preliminary testing of the new five-level version of EQ-5D (EQ-5D-5L). Qual Life Res. 2011;20(10):1727-36.

75. National Institute for Health Research. Good Clinical Practice Supporting Templates: Decision Tree for Adverse Event Reporting NON CTIMPS 2023 [Available from: https://learn.nihr.ac.uk/course/view.php?id=496.

76. Bennell KL, Nelligan RK, Rini C, Keefe FJ, Kasza J, French S, et al. Effects of internet-based pain coping skills training before home exercise for individuals with hip osteoarthritis (HOPE trial): a randomised controlled trial. Pain. 2018;159(9):1833-42.

77. Higgins J, Green S. Cochrane Handbook for Systematic Reviews of Interventions: The Cochrane Collaboration; 2011. Available from: www.handbook.cochrane.org.

78. Thabane L, Ma J, Chu R, Cheng J, Ismaila A, Rios LP, et al. A tutorial on pilot studies: the what, why and how. BMC Medical Research Methodology. 2010;10(1):1.

79. Sekhon M, Cartwright M, Francis JJ. Acceptability of healthcare interventions: an overview of reviews and development of a theoretical framework. BMC Health Services Research. 2017;17(1):88.

80. Staniszewska S, Brett J, Simera I, Seers K, Mockford C, Goodlad S, et al. GRIPP2 reporting checklists: tools to improve reporting of patient and public involvement in research. Res Involv Engagem. 2017;3(1):13.
