# Supplementary material for: Multidimensional fidelity of the EPIC-Neck exercise programme for chronic non-specific neck pain: a UK feasibility trial process evaluation
Source: BMJ Open. 2026 Oct 20;16(10):e121944. doi: 10.1136/bmjopen-2026-121944 (PMC13684230; doi:10.1136/bmjopen-2026-121944)
Supplement: Supplementary file 7 [file bmjopen-2026-121944.reviewer_comments.pdf]

PEER REVIEW HISTORY

BMJ Open publishes all reviews undertaken for accepted manuscripts. Reviewers are asked to complete a checklist review form and are provided with free text boxes to elaborate on their assessment. These free text comments are reproduced below.

ARTICLE DETAILS

Title (Provisional)

Multidimensional fidelity of the EPIC-Neck exercise programme for chronic non-specific neck pain: a UK feasibility-trial process evaluation

Authors

Price, Jonathan; Rushton, Alison; Ives, Natalie; Bradbury, Marilyn; Greaves, Colin J

VERSION 1 - REVIEW

**Reviewer**	**1**
**Name**	**Bagherian, Sajad**
**Affiliation**	**Shahrekord University**
**Date**	**10-Jun-2026**
**COI**	**None**

Title:

The title is too long. The phrase "co-developed, theory and evidence-based" seems redundant and can be removed without losing meaning. I suggest you shorten the title, for example: "Multidimensional fidelity of the EPIC-Neck exercise programme for chronic nonspecific neck pain: a process evaluation".

Abstract:

Results:

• You report a mean score of 2.4 out of 5, but the reader does not know that this is below the acceptable threshold because you did not state the threshold in the methods.

• The phrase "PO2-PO4 scored poorest" is not clear to readers who are unfamiliar with your protocol. Please replace the abbreviations with descriptive terms: "progressing/regressing exercise, flare-up management, and general exercise (PO2-PO4)".

• You state that 5 physiotherapists were interviewed out of 7, but you did not give the reason for excluding the other 2. If space allows, add "two declined".

Conclusion:

The conclusion is acceptable, but the sentence "training did not adequately prepare physiotherapist for delivery" is somewhat contradicted by your own data showing that median confidence increased from 3 to 4. Please soften this to: "training improved confidence but remained insufficient for complex components, and delivery fidelity was below the acceptable threshold".

BACKGROUND:

I have several concerns with the background section. First, the projection that neck pain disability will rise by "35% by 2044" is too precise for a single reference, and I am not convinced this forecast is robust enough for a peer-reviewed journal. Please rephrase or add supporting evidence. Second, the claim that chronic nonspecific neck pain accounts for "60% of clinical cases" is not clearly supported by references 2-6; some of these papers do not report this figure at all, so you need to verify and correct your citations. Third, when you state that EPIC-Neck was developed using Intervention Mapping, you should cite the original methodology (Bartholomew Eldredge et al., 2016) immediately after the term rather than only as reference 31 later in the paragraph. Fourth, the transition from describing the EPIC-Neck programme into fidelity evaluation is abrupt, and I suggest adding a brief bridging sentence before introducing the MRC guidance. Fifth, in objective 1 the word "adequately" is vague – please specify what constitutes adequate training, for example "sufficient to deliver the programme as intended". Finally, reference 35 appears before the full stop, which should be corrected to after the full stop following journal convention. Overall the rationale is clear, but the referencing needs careful revision.

METHODS:

I have several concerns with the methods section. First, the study design is described as a "mixed methods fidelity evaluation embedded in a randomised controlled feasibility study" but you do not state the primary aim of the main feasibility trial anywhere in this manuscript. Readers need to know what the main trial was testing to understand the context of this fidelity evaluation. Please add one sentence explaining the parent study. Second, you state that design fidelity was not assessed because "the theoretical processes and implementation of key components were outlined in detail during intervention development" – this justification is insufficient. The NIH-BCC framework includes design fidelity for a reason. Not assessing it is a limitation that should be acknowledged transparently, not justified away. Please rephrase as a limitation rather than a decision.

Third, the EPIC-Neck programme description is very long and some details are repeated from the background. Consider condensing this or moving detailed tables to supplementary files as you have already done. Fourth, in the quantitative data collection for delivery fidelity, you describe a process where two researchers coded appointments until "at least 75% of scores were within one point of each other" before a single researcher continued. You do not report the final inter-rater agreement percentage or kappa statistic. This is essential for reproducibility. Please add the final agreement figure.

Fifth, you state that enactment fidelity was "assessed qualitatively only, as appropriate enactment varied by context and scenario, making quantitative measurement difficult to operationalise." This is a weakness, not a justification. Many fidelity studies measure enactment quantitatively. Please acknowledge this as a limitation more clearly. Sixth, the interviewer (JP) was the chief investigator and lead intervention developer who had existing professional relationships with some physiotherapists. This carries a risk of social desirability bias, particularly for the physiotherapist interviews. You mention rapport and confidentiality but do not describe any specific strategies to minimise this bias, such as using an independent interviewer for at least a subset of interviews. Please address this.

Finally, you used NVivo for qualitative analysis but you do not report whether any independent double-coding was performed beyond review with the study team. A single coder (JP) raises concerns about reliability. Please clarify if any formal inter-coder reliability checks were conducted. Overall the methods are thorough but transparency around potential bias and missing fidelity domains needs improvement.

RESULTS:

I have several concerns with the results section. First, Table 2 presents participant characteristics but you report that 24 participants were randomised to EPIC-Neck, 18 returned quantitative receipt data, and 12 were interviewed. You do not explain why 6 participants did not return the receipt questionnaire (was it non-response, withdrawal, or loss to follow-up?) and why 5 of the 17 invited to interview declined. Please add brief explanations. Second, in the training fidelity results, you report that median confidence increased for 22/32 components, remained unchanged with reduced variability for 6/32 components, and showed no change for 4 components. However, you do not provide a statistical test (e.g., Wilcoxon signed-rank) to support these comparisons. Descriptive statistics alone are insufficient here. Please add appropriate non-parametric tests.

Third, and most concerning, the delivery fidelity results show an overall mean of 2.4/5, which is below your acceptable threshold of 3. However, you then report that usual exercise care had a mean fidelity score of 1.1/5. You are essentially using the same checklist to score a completely different intervention (usual care) and interpreting a score of 1.1 as "lower fidelity" to EPIC-Neck. This is methodologically problematic because usual care was never intended to follow the EPIC-Neck protocol. A low score on your EPIC-Neck checklist for usual care does not indicate "poor delivery" of usual care – it simply confirms that usual care is different. Please reinterpret these data or remove the comparison entirely. At minimum, change the language from "fidelity" to "concordance with EPIC-Neck protocol" for the usual care group.

Fourth, in Table 4, you colour-code components as green (3.0-5.0), amber (2.0-2.9), and red (0.0-1.9). This is helpful, but component 16 (Improving tolerance) has a mean of 2.7 (amber) with a very large standard deviation of 1.6. This suggests substantial variability across participants or physiotherapists. You do not explore this variability anywhere. Please add

commentary on why this component was so inconsistent. Fifth, for receipt fidelity, you report that 94% of participants were confident or very confident performing PO1, but qualitative data show that confidence was lower for complex or resisted exercises. This is a divergence you acknowledge, but you do not quantify how many participants fell into this subgroup. Please provide a rough estimate (e.g., "approximately half of interviewed participants reported difficulty with resisted exercises").

Sixth, for enactment fidelity, you report that "no participants started or resumed general exercise routines because of the intervention" (PO4). This is a striking null finding. However, you do not report how many participants were already active at baseline. If most participants were already doing general exercise, then "no change" is less surprising. Please add baseline physical activity levels for your interview subsample. Finally, the integration sections are well done but some claims are not fully supported. For example, you state "poorer delivery appeared to contribute to weaker receipt and enactment" but you cannot infer causation from this sequential association. Please rephrase to "was associated with" rather than "contributed to".

DISCUSSION:

I have several concerns with the discussion section. First, you open by stating that "prescribed exercise programmes were generally well received and enacted by patients, albeit selectively." However, your delivery fidelity was only 2.4/5, which is below acceptable. You seem to be downplaying this major finding. The discussion should more prominently acknowledge that the intervention was not delivered as intended, and this fundamentally limits what you can conclude about its potential effectiveness. Please restructure the opening paragraph to give equal weight to the delivery failure.

Second, you claim that "poorer delivery appears to contribute to weaker receipt and enactment" and that this demonstrates "sequential influence" across domains. This is an overstatement. Your study is observational and correlational. You have not experimentally manipulated delivery quality to test its effect on receipt or enactment. The phrase "appears to contribute" is acceptable, but later you say "this highlights the importance of improving delivery quality, given its likely influence" – this is speculative. Please tone down causal language throughout.

Third, you compare your findings to other studies reporting "only moderate exercise adherence" and suggest that EPIC-Neck may support better engagement for PO1. However, you did not measure adherence quantitatively (e.g., exercise diary completion rates, percentage of prescribed exercises performed). Your enactment data are qualitative only and based on participant self-report at discharge. This comparison is not valid. Either remove this claim or add a strong caveat acknowledging the lack of quantitative adherence data.

Fourth, you state that "no participants started or resumed general exercise (PO4)" and attribute this to poor delivery of PO4 components. This ignores an alternative explanation: the follow-up period may have been too short. Discharge occurred after a mean of 16.9

weeks. Behaviour change for general exercise often takes longer. You acknowledge this partially in the limitations but then ignore it in your main interpretation. Please discuss this alternative explanation more explicitly.

Fifth, you argue that training should move from a "single-day, high-volume model" to a "staged training model" with practice and feedback. This is a reasonable suggestion, but you provide no evidence from your data about what an alternative training model would look like or how many days would be sufficient. Please be more specific or present this as a hypothesis rather than a firm recommendation.

Sixth, you list several limitations but you missed at least three important ones: (1) the fidelity checklist was designed by the intervention developer (JP), introducing potential bias; (2) you did not assess inter-rater reliability beyond 75% agreement within 1 point, which is a weak threshold; and (3) the physiotherapists knew they were being audio-recorded, which may have altered their behaviour (Hawthorne effect). Please add these to the limitations paragraph.

Finally, the conclusion states that "optimisation of intervention content, training, and implementation processes, followed by repeat fidelity evaluation, appears warranted" before a definitive trial. I agree, but this is obvious given your findings. A stronger conclusion would specify the top three specific changes you recommend (e.g., (1) extend training to two days with supervised practice, (2) simplify PO4 delivery, (3) add individualised feedback on audio-recorded sessions). Please be more specific.

TABLES AND FIGURES:

I have several concerns with the tables and figures. First, Table 1 is very useful but it is split awkwardly across pages 14 and 15 with "NA" and empty cells that are hard to follow. Please reformat this table so that it fits entirely on one page or restructure it vertically. The column "Integration" also contains too much text. Consider moving the integration descriptions to the main text and leaving only the design type (e.g., "Convergent", "Explanatory sequential") in the table.

Second, Table 2 presents participant characteristics but the column headings are confusing. You have "EPIC-Neck n=24", "Usual exercise care n=12", "Receipt at 6-months (questionnaire) n=18", and "Receipt/enactment (interviews) n=12". These are nested subsets, but the table makes them look like four separate groups. Please clarify using indentation or footnotes that the last two columns are subsets of the EPIC-Neck column. Also, percentages for the interview subsample (n=12) are sometimes misleading because the numbers are very small. For example, 8.3% for age 18-30 represents only one person. Consider suppressing percentages for any subgroup with n<5 or adding a footnote.

Third, Table 3 reports physiotherapist characteristics. You show "Trained: Confidence and survey data n=9" but earlier in the results you state that 9 physiotherapists were trained and 7 delivered EPIC-Neck. However, in Table 3 you do not explain why 2 of the 9 trained did not

deliver. Please add a footnote explaining this (e.g., "Two trained physiotherapists left the service before delivering the programme").

Fourth, Table 4 is the most important table but it is difficult to read. The component labels (01 through 17) are not descriptive enough. A reader should not have to keep looking back at the methods to understand what "03" means. Please add brief descriptive labels in parentheses, for example: "03 - Improve motivation (perceived importance and safety)". Also, the colour coding (green, amber, red) is helpful but you do not specify in the table footnote what the colours mean for the usual exercise care column. Is red still "major delivery concerns" for usual care? As I noted in the results section, scoring usual care against an EPIC-Neck checklist is problematic. At minimum, add a strong footnote stating that these scores measure concordance with EPIC-Neck, not fidelity to usual care.

Fifth, Table 5 is excellent. The integration interpretation column is very clear. However, you have a footnote for PO4 stating that "enactment of secondary-prevention exercise was not expected and was not assessed". This contradicts your own logic model. If it was not expected, why is it a performance objective? Please clarify this in the main text, not just in a footnote.

Sixth, Figure S1 and S2 (the frameworks) are visually appealing but the font size is too small. When printed, the text in these figures will be illegible. Please increase the font size or provide these as separate high-resolution files. Finally, several supplementary tables (especially Table S4) contain excessive text in the "Interview derived barriers" and "Interview derived facilitators" columns. Consider moving this detailed qualitative data into the main results narrative and summarising the table more concisely, or splitting Table S4 into two separate tables.

**Reviewer**	**2**
**Name**	**Hernández-Beltrán, Víctor**
**Affiliation**	**Universidad de Extremadura**
**Date**	**16-Jun-2026**
**COI**	**None**

The authors present a good research paper.

• Relevance of the topic: Excellent.

• Introduction: Good.

• Methodology: Regular.

• Results: Good.

• Discussion: Good.

• Conclusions: Bad

However, Reconsider after major revision. In general, the paper follows an adequate structure and correct scientific support and can be published considering some limitations. However, there are a series of limitations that should be considered.

Specific comments.

The author must rewrite the whole text in an impersonal manner and try not to use the pronoun “we”. In the same way, reviewing each section's verb tense is recommended. Results (past tense), Introduction (past tense), Method (past tense), Results (past tense), Discussion (present tense), and Conclusions (present tense).

4.1. Title and abstract

• The title is clear and appropriate.

• The abstract is comprehensive, but could place greater emphasis on low adherence to the intervention and its implications.

4.2. Introduction

Well-structured and the use of references is correct, and they are updated. However, this section could be improved:

• Include more recent evidence on adherence to exercise in musculoskeletal pain.

• Better explain why the NIH-BCC was chosen over other frameworks.

4.3. Methods

The methods and procedures followed are well defined and explain the main information about the study in order to be replicated. In this line, some issues can be explained:

• Why did you select a threshold of ≥3/5 was chosen as ‘acceptable’

• Justify the decision not to assess design fidelity beyond the reference to the previous article.

4.4. Results

Clearly presented, but:

• The table showing fidelity by component should include confidence intervals.

• It would be useful to add a flowchart of participants in the fidelity assessment.

4.5. Discussion

This section is well structured and discusses the main results of the study according to previous research. However, some sentences and affirmations are too descriptive. A critical analysis is lacking regarding:

• The actual feasibility of the programme within the NHS.

• The need to redesign behavioural change components.

• The possible simplification of the programme.

4.6. Conclusions

• It is necessary to include a review of the training.

• To summarise the programme before a definitive trial.

**Reviewer**	**3**
**Name**	**Saadat, Maryam**
**Affiliation**	**Ahvaz Jundishapur University of Medical Sciences**
**Date**	**19-Jun-2026**
**COI**	**None**

Abstract

• The title of the manuscript appears overly long and somewhat cluttered, which makes it difficult to immediately grasp the main focus of the study. A more concise and clearer title would improve readability and help readers quickly understand the key message of the paper.

• The abbreviation "EPIC Neck" is introduced without first providing its full term. Please ensure that the full name of the intervention is written out at its first appearance in the manuscript, followed by the abbreviation in parentheses.

Introduction

• The EPIC Neck program is briefly introduced in the Introduction; however, the description remains quite general.

Method

• Include references for line 147 to 149

• The manuscript refers to participants with "non specific neck pain"; however, the definition of this condition is not clearly described. The authors should clarify the diagnostic criteria used and who was responsible for making the diagnosis

• The study appears to include two groups; however, this section refers only to participants randomized to the EPIC Neck intervention. The authors should clarify whether the fidelity evaluation was conducted exclusively for the intervention group and explain how participants in the control group were considered in the study. Providing a clearer explanation would help readers better understand the analytical approach.

• The manuscript states that physiotherapists completed a questionnaire before and after the training; however, the questionnaire is not clearly described. The authors should clarify

whether this was a previously validated questionnaire or a study specific instrument developed for this research.

• Explain more details from delivery checklist and receipt questionnaire

Results

• The authors state that "distinct differences" were noted between the EPIC-Neck and Usual Exercise Care groups. While the descriptive data (means and SDs) support this, the authors should clarify if any statistical significance testing (e.g., t-tests or non-parametric equivalents) was performed to support the use of the term "distinct differences." If not, it might be more appropriate to describe these as "descriptive differences."

VERSION 1 - AUTHOR RESPONSE

Reviewer 1

	Section	Feedback	Response	Updated Text
1.1	Title/ Abstract	The title is too long. The phrase "co-developed, theory and evidence-based" seems redundant and can be removed without losing meaning. I suggest you shorten the title, for example: "Multidimensional fidelity of the EPIC-Neck exercise programme for chronic nonspecific neck pain: a process evaluation".	Thank you for the feedback. The title has been shortened and the country has been added.	**Original:**<br>Multidimensional fidelity of a co-developed, theory and evidence-based exercise programme for chronic non-specific neck pain: a process evaluation of the EPIC-Neck programme<br><br>**Updated:**<br>Multidimensional fidelity of the EPIC-Neck exercise programme for chronic non-specific neck pain: a UK feasibility-trial process evaluation
1.2	Title/ Abstract	You report a mean score of 2.4 out of 5, but the reader does not know that this is below the acceptable threshold because you did not state the threshold in the methods.	Thank you for this comment. We respectfully note that the acceptable threshold is already stated in the abstract methods under "Fidelity evaluation" ("0–5; 3 = acceptable"). We therefore did not make changes in response to this comment.	No changes were made to the manuscript in response to this comment.
1.3	Title/ Abstract	The phrase "PO2-PO4 scored poorest" is not clear to readers who are unfamiliar with your protocol. Please replace the abbreviations with descriptive terms: "progressing/regressing exercise, flare-up	Thank you for this comment. The performance objectives are defined earlier in the abstract under "Intervention." "four performance objectives (POs): perform prescribed exercise (PO1), progress/regress exercise (PO2), use exercise to manage flare-ups (PO3), and engage in general exercise (PO4)."	No changes were made to the manuscript in response to this comment.

	Section	Feedback	Response	Updated Text
		management, and general exercise (PO2-PO4)".	In the interests of brevity, and given the editor's request to reduce the length of the abstract results, we did not make further changes in response to this comment.	
1.4	Title/ Abstract	You state that 5 physiotherapists were interviewed out of 7, but you did not give the reason for excluding the other 2. If space allows, add "two declined".	Thank you for this suggestion. This has already been clarified in the main manuscript, which states that the remaining two physiotherapists declined due to sick/bereavement leave. We have not added this detail to the abstract in view of the editor's request to reduce the length of the abstract results section.	No changes were made to the manuscript in response to this comment.
1.5	Title/ Abstract	The conclusion is acceptable, but the sentence "training did not adequately prepare physiotherapist for delivery" is somewhat contradicted by your own data showing that median confidence increased from 3 to 4. Please soften this to: "training improved confidence but remained insufficient for complex components, and delivery fidelity was below the acceptable threshold".	Thank you for this comment. We agree that the conclusion should align clearly with the training findings reported in the abstract. The abstract already states that confidence improved while qualitative findings indicated that physiotherapists still felt underprepared. To reflect this more clearly and avoid any perceived overstatement, we have revised the conclusion wording.	**Original (Abstract):**<br>Although beneficial, training did not adequately prepare physiotherapist for delivery.<br><br>**Updated:**<br>Although training improved confidence, it did not fully prepare physiotherapists for delivery of more complex components.
1.6	Background	I have several concerns with the background section. First, the projection that neck pain disability will rise by "35% by 2044" is too precise for a single reference, and I am not convinced this forecast is robust enough for a peer-reviewed journal. Please rephrase or add supporting evidence.	Thank you for this comment. We have revised the wording to report the source paper's projected age-standardised YLD rates directly and to make clear that these are modelled estimates.	**Original (Background):**<br>Global age-standardised years lived with disability attributable to neck pain have increased over the past decade and are projected to rise by a further 35% by 2044.1<br><br>**Updated:**<br>Global Burden of Disease modelling indicates that the global age-standardised years lived with disability rate attributable to neck pain has increased in recent years and is projected to rise by 35% (from

	Section	Feedback	Response	Updated Text
				267.348 per 100,000 in 2019 to 360.034 per 100,000) by 2044.1
1.7	Background	Second, the claim that chronic nonspecific neck pain accounts for "60% of clinical cases" is not clearly supported by references 2-6; some of these papers do not report this figure at all, so you need to verify and correct your citations.	Thank you for this comment. We have removed this specific figure and revised the wording to focus on the clinical importance of chronic/recurrent neck pain, including its association with disability, recurrent symptoms and healthcare use.	**Original (Background):**<br>Chronic non-specific neck pain, which accounts for around 60% of clinical cases, contributes most to this burden because of its high prevalence and associated disability.2-6<br><br>**Updated:**<br>Chronic non-specific neck pain contributes substantially to this burden because symptoms are often recurrent or persistent and are associated with disability, psychological distress and healthcare use.2-6
1.8	Background	Third, when you state that EPIC-Neck was developed using Intervention Mapping, you should cite the original methodology (Bartholomew Eldredge et al., 2016) immediately after the term rather than only as reference 31 later in the paragraph.	Thank you for this comment. The original Intervention Mapping methodology reference was already cited immediately after first mention of the term, alongside the intervention development paper ("…developed using Intervention Mapping 32 33"). We therefore did not make further changes in response to this comment.	No changes were made to the manuscript in response to this comment.
1.9	Background	Fourth, the transition from describing the EPIC-Neck programme into fidelity evaluation is abrupt, and I suggest adding a brief bridging sentence before introducing the MRC guidance.	Thank you for this suggestion. We have added a bridging sentence to explain that, given the multiple interacting components within EPIC-Neck, assessing whether the programme can be delivered, received and enacted as intended is an important step before definitive effectiveness testing.	**Original (Background):**<br>The programme was designed to maximise engagement with and the effectiveness of neck exercise for people with chronic non-specific neck pain.<br><br>The UK Medical Research Council recommends that feasibility studies evaluate fidelity to identify implementation uncertainties that may influence intervention effectiveness and to inform refinement prior to a definitive trial 31 34.<br><br>**Updated:**<br>The programme was designed to maximise engagement with and the effectiveness of neck

	Section	Feedback	Response	Updated Text
				exercise for people with chronic non-specific neck pain.<br><br>Given the multiple interacting components within EPIC-Neck, understanding whether the programme can be delivered, received and enacted as intended is an essential step before definitive effectiveness testing.31 34 The UK Medical Research Council recommends that feasibility studies evaluate fidelity to identify implementation uncertainties that may influence intervention effectiveness and to inform refinement prior to a definitive trial.31 34
1.10	Background	Fifth, in objective 1 the word "adequately" is vague – please specify what constitutes adequate training, for example "sufficient to deliver the programme as intended".	Thank you for this comment. We have revised the objective.	**Original (Aims and objectives):**<br>Training fidelity: Assess whether the training adequately developed physiotherapists' capability (skills, knowledge, confidence) to deliver EPIC-Neck and identify areas for refinement.<br><br>**Updated:**<br>Training fidelity: Assess the extent to which training developed physiotherapists' capability (skills, knowledge and confidence) to deliver EPIC-Neck as intended and identify areas for refinement.
1.11	Background	Finally, reference 35 appears before the full stop, which should be corrected to after the full stop following journal convention. Overall the rationale is clear, but the referencing needs careful revision.	Thank you for this comment. Reference formatting has been checked and amended throughout the manuscript.	-
1.12	Methods	I have several concerns with the methods section. First, the study design is described as a "mixed methods fidelity evaluation embedded in a randomised	Thank you for these comments. We have revised the Study design section to state the aim of the parent feasibility trial before describing the nested multidimensional fidelity	**Original (Methods):**<br>Study design<br>A mixed methods fidelity evaluation was embedded in the EPIC-Neck randomised controlled feasibility study,

	Section	Feedback	Response	Updated Text
		controlled feasibility study" but you do not state the primary aim of the main feasibility trial anywhere in this manuscript. Readers need to know what the main trial was testing to understand the context of this fidelity evaluation. Please add one sentence explaining the parent study.	evaluation. We have clarified that the multidimensional fidelity evaluation focused on the EPIC-Neck intervention. Usual exercise care appointments were included only to assess concordance with EPIC-Neck components and explore intervention distinctness/contamination, rather than to evaluate fidelity of usual care to its own intended model.	where patients attending NHS musculoskeletal outpatient physiotherapy services in the UK were randomised to receive the EPIC-Neck programme or usual exercise care (physiotherapists' routine practice).44<br><br>Multidimensional fidelity evaluation<br>EPIC-Neck fidelity evaluation was guided by the NIH-BCC framework covering training, delivery, receipt and enactment, using a mixture of qualitative and quantitative methods (Table 1)36 41 56 57.<br><br>**Updated:**<br>Study design<br>This mixed-methods process evaluation was embedded within the EPIC-Neck randomised controlled feasibility study, which aimed to determine the feasibility of a definitive trial evaluating the clinical and cost-effectiveness of EPIC-Neck compared with usual exercise care for adults with chronic non-specific neck pain. In the parent feasibility study, patients attending UK NHS musculoskeletal outpatient physiotherapy services were randomised to EPIC-Neck or usual exercise care (physiotherapists' routine practice).41 The present study reports the nested multidimensional fidelity evaluation of the EPIC-Neck intervention. Usual exercise care appointments were included only to examine whether EPIC-Neck components appeared in usual care.<br><br>Multidimensional fidelity

	Section	Feedback	Response	Updated Text
				Fidelity evaluation of the EPIC-Neck programme was guided by the NIH-BCC framework covering training, delivery, receipt and enactment, using a mixture of qualitative and quantitative methods (Table 1).36 41 56 57
1.13	Methods	Second, you state that design fidelity was not assessed because "the theoretical processes and implementation of key components were outlined in detail during intervention development" – this justification is insufficient. The NIH-BCC framework includes design fidelity for a reason. Not assessing it is a limitation that should be acknowledged transparently, not justified away. Please rephrase as a limitation rather than a decision.	Thank you for these comments. We have revised the Methods section to clarify that design fidelity was addressed during intervention development, where the theoretical mechanisms, active components and intended implementation of EPIC-Neck were specified using Intervention Mapping methods and reported previously. This process evaluation focused on empirically assessing training, delivery, receipt and enactment fidelity. We now also acknowledge that no additional independent assessment of design fidelity was undertaken within this study, and that this is considered in the limitations section (see response 1.38).	**Original (Methods – Multidimensional fidelity evaluation):**<br>Design fidelity was not assessed because the theoretical processes and the implementation of key components were outlined in detail during intervention development using intervention mapping methods,34 ensuring a good fit between the intervention content and the intervention logic model /underpinning theoretical mechanisms<br><br>**Updated:**<br>Design fidelity was addressed during intervention development, where the theoretical mechanisms, active components and intended implementation of EPIC-Neck were specified using Intervention Mapping methods and reported previously.34 This process evaluation therefore focused on empirically assessing training, delivery, receipt and enactment fidelity.
1.14	Methods	Third, the EPIC-Neck programme description is very long and some details are repeated from the background. Consider condensing this or moving detailed tables to supplementary files as you have already done.	Thank you for this comment. We have condensed this section by removing repeated detail and shortening the descriptions of the exercise prescription framework and behaviour change facilitation guide, while retaining key details required to understand the fidelity evaluation. It has taken the word count for the EPIC-Neck programme description from 446 words to 340 words.	See "EPIC-Neck programme" in the manuscript

	Section	Feedback	Response	Updated Text
			Describing the intervention in some detail is of course necessary for this paper as the purpose is to assess the fidelity of its delivery.	
1.15	Methods	Fourth, in the quantitative data collection for delivery fidelity, you describe a process where two researchers coded appointments until "at least 75% of scores were within one point of each other" before a single researcher continued. You do not report the final inter-rater agreement percentage or kappa statistic. This is essential for reproducibility. Please add the final agreement figure.	Thank you for this comment. We have reported the final agreement percentages the Results section "(two researchers scoring two appointments each) yielded 67% agreement. Following discussion, agreement increased to 84% then 82% (across a further four, then three appointments respectively), before the remaining recordings were scored by a single researcher (JP)." We did not calculate a kappa statistic.	No changes were made to the manuscript in response to this comment, as the final agreement figures were already reported in the Results section.
1.16	Methods	Fifth, you state that enactment fidelity was "assessed qualitatively only, as appropriate enactment varied by context and scenario, making quantitative measurement difficult to operationalise." This is a weakness, not a justification. Many fidelity studies measure enactment quantitatively. Please acknowledge this as a limitation more clearly.	Thank you for this comment. We have revised the Methods section to explain that quantitative enactment measurement was considered during study development but was not included because, for the EPIC-Neck intervention, meaningful enactment varied by performance objective and context, and because further questionnaire items would have increased participant burden and complexity. Specifically, PO2 involved nuanced decisions about adapting, progressing or regressing exercises; PO3 was conditional on participants experiencing a flare-up; and PO4 involved decision-making about general exercise and optional secondary-prevention neck exercise. Following PPIE feedback, proposed items to measure enactment of performance objectives quantitatively were removed, and qualitative interviews were used to explore enactment in	**Original (Methods – Data collection – Quantitative – Enactment)**<br>Enactment fidelity was assessed qualitatively only, as appropriate enactment varied by context and scenario, making quantitative measurement difficult to operationalise.<br><br>**Updated:**<br>Enactment fidelity was assessed qualitatively through participant interviews. Quantitative enactment measurement was considered during study development but was not included because meaningful enactment varied by performance objective and context. For example, PO2 involved nuanced decisions about adapting, progressing or regressing exercises; PO3 was only applicable when participants experienced flare-ups; and PO4 involved decision-making about general exercise and optional secondary-

	Section	Feedback	Response	Updated Text
			context. We acknowledge that quantitative measurement may be more feasible for PO1 if conceptualised as exercise adherence, for example using tools such as ATEMPT. However, ATEMPT was published after this study was planned. However, the use of such quantitative measures will be considered further when progressing to a definitive trial to establish long-term effectiveness and engagement. This is acknowledged in the limitations section (see response 1.38)	prevention neck exercise. Based on PPIE input, proposed quantitative performance-objective measures were removed to reduce participant burden and because they risked being too crude to capture the complexity of enactment in this context.
1.17	Methods	Sixth, the interviewer (JP) was the chief investigator and lead intervention developer who had existing professional relationships with some physiotherapists. This carries a risk of social desirability bias, particularly for the physiotherapist interviews. You mention rapport and confidentiality but do not describe any specific strategies to minimise this bias, such as using an independent interviewer for at least a subset of interviews. Please address this.	Thank you for this comment. We have acknowledged this more clearly in the Methods section and described the strategies used to reduce this risk. We have also acknowledged this in the limitations section (see response 1.38).	**Original:**<br>Data collection: Qualitative Interviews were conducted by JP by telephone, Microsoft Teams, or face-to-face, lasted up to 90 minutes, and were audio-recorded and transcribed verbatim. JP (chief investigator and lead intervention developer) had existing professional relationships with some physiotherapists (none with participants) and did not deliver the intervention. Rapport, reflective listening, and confidentiality were emphasised. Notes were taken, but no reflexive journal kept. A structured topic guide supported consistency and minimised bias.<br><br>**Updated:**<br>Data collection: Qualitative Interviews were conducted by JP by telephone, Microsoft Teams, or face-to-face, lasted up to 90 minutes, and were audio-recorded and transcribed verbatim. JP was the chief investigator and lead intervention developer, had existing professional relationships with some physiotherapists, but did not deliver the intervention. To

	Section	Feedback	Response	Updated Text
				mitigate social desirability and allegiance bias, particularly in physiotherapist interviews, JP used a structured topic guide, emphasised confidentiality and neutrality, used reflective listening, and encouraged participants to describe both positive and negative experiences. Brief notes were taken after interviews; no reflexive journal was maintained.
1.18	Methods	Finally, you used NVivo for qualitative analysis but you do not report whether any independent double-coding was performed beyond review with the study team. A single coder (JP) raises concerns about reliability. Please clarify if any formal inter-coder reliability checks were conducted. Overall the methods are thorough but transparency around potential bias and missing fidelity domains needs improvement.	Thank you for this comment. No formal independent double-coding or inter-coder reliability checks were conducted. We have clarified this in the Methods section and acknowledged it more explicitly as a limitation.	**Original (Methods – Data analysis – Qualitative):**<br>JP coded and analysed all interview transcripts.<br><br>**Updated:**<br>Due to resource limitations, JP coded and analysed all interview transcripts. No formal independent double-coding or inter-coder reliability checks were conducted.
1.19	Results	I have several concerns with the results section. First, Table 2 presents participant characteristics but you report that 24 participants were randomised to EPIC-Neck, 18 returned quantitative receipt data, and 12 were interviewed. You do not explain why 6 participants did not return the receipt questionnaire (was it non-response, withdrawal, or loss to follow-up?) and why 5 of the 17 invited to interview declined. Please add brief explanations.	Thank you for this comment. We have clarified that the six participants who did not contribute quantitative receipt data did not return the 6-month questionnaire despite follow-up. We respectfully note that reasons for interview non-participation were already reported in the Results section: "Seventeen participants were invited to interviews and 12 took part. Two were not contactable, and three declined due to not wanting to use a professional interpreter (n = 2) or a lack of time (n = 1)."	**Original (Results – patient participant demographics):**<br>Eighteen out of 24 EPIC-Neck intervention participants returned quantitative receipt data.<br><br>**Updated:**<br>Eighteen out of 24 EPIC-Neck intervention participants returned quantitative receipt data; the remaining six did not return the 6-month questionnaire despite follow-up.
1.20	Results	Second, in the training fidelity results, you report that median confidence increased for 22/32	Thank you for this comment. The training-confidence data were collected primarily to support descriptive assessment of	**Updated:**<br><br>Methods - As an exploratory analysis, overall pre/post training

	Section	Feedback	Response	Updated Text
		components, remained unchanged with reduced variability for 6/32 components, and showed no change for 4 components. However, you do not provide a statistical test (e.g., Wilcoxon signed-rank) to support these comparisons. Descriptive statistics alone are insufficient here. Please add appropriate non-parametric tests.	training fidelity within a small-sample process evaluation, rather than to undertake formal hypothesis testing. However, we agree that a paired non-parametric test may help readers interpret the overall pre/post change in confidence. We have therefore added an exploratory Wilcoxon signed-rank test comparing each physiotherapist's overall mean confidence score across all training components before and after training.	confidence was compared using a Wilcoxon signed-rank test, based on each physiotherapist's mean confidence score across all training components.<br><br>Results - An exploratory Wilcoxon signed-rank test of physiotherapists' overall mean confidence scores across all components indicated a statistically significant increase from baseline to post-training ($Z = -2.55$, $p = .011$).
1.21	Results	Third, and most concerning, the delivery fidelity results show an overall mean of 2.4/5, which is below your acceptable threshold of 3. However, you then report that usual exercise care had a mean fidelity score of 1.1/5. You are essentially using the same checklist to score a completely different intervention (usual care) and interpreting a score of 1.1 as "lower fidelity" to EPIC-Neck. This is methodologically problematic because usual care was never intended to follow the EPIC-Neck protocol. A low score on your EPIC-Neck checklist for usual care does not indicate "poor delivery" of usual care – it simply confirms that usual care is different. Please reinterpret these data or remove the comparison entirely. At minimum, change the language from "fidelity" to "concordance with EPIC-Neck protocol" for the usual care group.	Thank you for this comment. We agree that usual exercise care should not be interpreted as having "fidelity" to the EPIC-Neck protocol, as usual care was not intended to deliver EPIC-Neck. We have therefore revised the Results section and Table 4 footnote to clarify that usual exercise care appointments were scored against the EPIC-Neck checklist to assess the presence of EPIC-Neck components (which would indicate potential contamination between study arms), rather than to assess fidelity of usual care to its own intended model. It is really important to establish that EPIC-Neck differs from usual care to help establish the rationale for progressing to a full-scale RCT (i.e. the premise that EPIC-Neck might deliver advantages over usual care is premised on the idea that EPIC-Neck delivers something different to usual care – and our data support this premise).	**Original (Results – Delivery – Quantitative):**<br>Distinct differences were noted between EPIC-Neck and Usual Exercise Care. Usual Exercise Care had a mean fidelity score of 1.1 (SD 1.0; range 0.6–1.5), with only one component (improving self-efficacy) delivered in a manner consistent with the EPIC-Neck protocol (Table 4). Full participant-by-component fidelity scores for both study arms are provided in Supplementary File 1 – Table S5-6.<br><br>**Updated:**<br>Usual exercise care appointments contained few EPIC-Neck components, suggesting limited contamination between study arms. When scored against the EPIC-Neck checklist, usual exercise care had a mean concordance score of 1.1 (SD 1.0; range 0.6–1.5). These descriptive differences suggest that usual exercise care rarely incorporated EPIC-Neck components. Only one component (improving self-efficacy) was delivered in a manner consistent with the EPIC-Neck protocol (Table 4). Full

	Section	Feedback	Response	Updated Text
				participant-by-component checklist scores for both study arms are provided in Supplementary File 1 – Tables S5–S6.<br><br>Table 4 footnote<br>Usual exercise care scores represent concordance with EPIC-Neck components and should not be interpreted as indicating poor quality of usual exercise care
1.22	Results	Fourth, in Table 4, you colour-code components as green (3.0-5.0), amber (2.0-2.9), and red (0.0-1.9). This is helpful, but component 16 (Improving tolerance) has a mean of 2.7 (amber) with a very large standard deviation of 1.6. This suggests substantial variability across participants or physiotherapists. You do not explore this variability anywhere. Please add commentary on why this component was so inconsistent.	Thank you for this comment. We have added a sentence to the Discussion noting that variability in "Improving tolerance".	**Updated (Discussion – delivery barriers reflected clinician behaviour change and training design):**<br>Overall, few EPIC-Neck components were delivered acceptably (≥3), with a range of barriers explaining delivery challenges. However, poorer delivery was concentrated in components that were novel, complex, skill-intensive, and less aligned with routine clinical practice, echoing findings from fidelity evaluations of other complex musculoskeletal interventions.73-75 Variability (indicated by a high standard deviation in fidelity scores) was also evident within some components, particularly "Improving tolerance", which likely reflected differences in physiotherapists' confidence and clinical reasoning around progressive strengthening/loading, as well as patient-level variation in readiness, symptoms and tolerance. This suggests that physiotherapists need to develop new skills and change their own behaviour to integrate EPIC-Neck components into routine consultations.76-80
1.23	Results	Fifth, for receipt fidelity, you report that 94% of	Thank you for this comment. We agree that the divergence	**Original (Results – Receipt – Qualitative):**

	Section	Feedback	Response	Updated Text
		participants were confident or very confident performing PO1, but qualitative data show that confidence was lower for complex or resisted exercises. This is a divergence you acknowledge, but you do not quantify how many participants fell into this subgroup. Please provide a rough estimate (e.g., "approximately half of interviewed participants reported difficulty with resisted exercises").	between the quantitative and qualitative receipt findings for PO1 could be expressed more clearly. As the qualitative analysis was not undertaken using estimated frequencies for various codes (and such an approach would not be robust with these relatively small numbers), we have not added numerical estimates. Instead, we have revised the qualitative and integration wording to make clearer that confidence and understanding were generally good overall, but were relatively lower for technically complex or resisted movements.	Most participants understood and felt confident performing exercises, but this was lower for complex or resisted exercises.<br><br>**Updated:**<br>Most participants understood and felt confident performing exercises, but several reported lower confidence and understanding for complex or resisted exercises.<br><br>**Original (Results – Receipt – Integration):**<br>Datasets agreed that participants were confident and understood how to perform their prescribed exercise programme. Qualitative data complemented this by showing that confidence and understanding were lower for technically complex or resisted movements and highlighting physiotherapist demonstrations as a key facilitator of understanding.<br><br>**Updated:**<br>Datasets agreed that participants were confident and understood how to perform their prescribed exercise programme. Qualitative data complemented this by showing that confidence and understanding were generally good, but were relatively lower for technically complex or resisted movements, and by highlighting physiotherapist demonstrations as a key facilitator of understanding.
1.24	Results	Sixth, for enactment fidelity, you report that "no participants started or resumed general exercise routines because of the intervention" (PO4). This is a striking null finding. However, you do not report	Thank you for this comment. Although, in retrospect, it would be interesting to see this data, baseline physical activity was not collected.	No changes made

	Section	Feedback	Response	Updated Text
		how many participants were already active at baseline. If most participants were already doing general exercise, then "no change" is less surprising. Please add baseline physical activity levels for your interview subsample.		
1.25	Tables/ Figures	I have several concerns with the tables and figures. First, Table 1 is very useful but it is split awkwardly across pages 14 and 15 with "NA" and empty cells that are hard to follow. Please reformat this table so that it fits entirely on one page or restructure it vertically. The column "Integration" also contains too much text. Consider moving the integration descriptions to the main text and leaving only the design type (e.g., "Convergent", "Explanatory sequential") in the table.	Thank you for this comment. We have revised Table 1 to improve readability and reduce its length so that it now fits on one page.	See Table 1
1.26	Tables/ Figures	Second, Table 2 presents participant characteristics but the column headings are confusing. You have "EPIC-Neck n=24", "Usual exercise care n=12", "Receipt at 6-months (questionnaire) n=18", and "Receipt/enactment (interviews) n=12". These are nested subsets, but the table makes them look like four separate groups. Please clarify using indentation or footnotes that the last two columns are subsets of the EPIC-Neck column. Also, percentages for the interview subsample (n=12) are sometimes misleading	Thank you for this comment. We have revised Table 2 to focus only on EPIC-Neck participants and amended the column headings to clarify participants who were randomised, returned the 6-month receipt questionnaire, and completed receipt/enactment interviews. We have also added a footnote stating that the questionnaire and interview columns are subsets of the EPIC-Neck randomised group.<br><br>We have retained percentages alongside n values because the table is used to describe inclusivity and sample composition, including ethnicity categories. However, we have	See table 2

	Section	Feedback	Response	Updated Text
		because the numbers are very small. For example, 8.3% for age 18-30 represents only one person. Consider suppressing percentages for any subgroup with n<5 or adding a footnote.	added a footnote noting that percentages should be interpreted cautiously where subgroup denominators are small.	
1.27	Tables/ Figures	Third, Table 3 reports physiotherapist characteristics. You show "Trained: Confidence and survey data n=9" but earlier in the results you state that 9 physiotherapists were trained and 7 delivered EPIC-Neck. However, in Table 3 you do not explain why 2 of the 9 trained did not deliver. Please add a footnote explaining this (e.g., "Two trained physiotherapists left the service before delivering the programme").	Thank you for this comment. We have clarified in the Results text why two trained physiotherapists did not deliver EPIC-Neck.	**Original (Results – physiotherapist characteristics):**<br>Of these, seven delivered the EPIC-Neck programme.<br><br>**Updated:**<br>Of these, seven delivered the EPIC-Neck programme; one trained physiotherapist left the service before delivering the intervention, and one did not deliver because no eligible participants were booked into their diary.
1.28	Tables/ Figures	Fourth, Table 4 is the most important table but it is difficult to read. The component labels (01 through 17) are not descriptive enough. A reader should not have to keep looking back at the methods to understand what "03" means. Please add brief descriptive labels in parentheses, for example: "03 - Improve motivation (perceived importance and safety)".	Thank you for this comment. We respectfully note that Table 4 already includes descriptive component labels alongside the numeric identifiers (for example, “03 - Improve motivation to perform exercise (PO1) - Enhance perceived importance and safety”). We therefore did not make substantive changes in response to this comment.	No changes were made to the manuscript in response to this comment.
1.29	Tables/ Figures	Fourth, Table 4. Also, the colour coding (green, amber, red) is helpful but you do not specify in the table footnote what the colours mean for the usual exercise care column. Is red still "major delivery concerns" for usual care? As I noted in the results	Thank you for this comment. We agree that applying the traffic-light colour coding to the usual exercise care column could imply that lower scores represented poor delivery of usual care, which was not intended. We have therefore removed colour coding from the usual exercise care column and	See Table 4

	Section	Feedback	Response	Updated Text
		section, scoring usual care against an EPIC-Neck checklist is problematic. At minimum, add a strong footnote stating that these scores measure concordance with EPIC-Neck, not fidelity to usual care.	revised the Table 4 legend to clarify that colour coding applies only to EPIC-Neck delivery fidelity. We have also added a footnote stating that usual exercise care scores indicate the extent to which EPIC-Neck components were present in usual care appointments and should not be interpreted as fidelity of usual care.	
1.30	Tables/ Figures	Fifth, Table 5 is excellent. The integration interpretation column is very clear. However, you have a footnote for PO4 stating that "enactment of secondary-prevention exercise was not expected and was not assessed". This contradicts your own logic model. If it was not expected, why is it a performance objective? Please clarify this in the main text, not just in a footnote.	Thank you for this comment. We agree that the dual nature of PO4 could be clearer. We have therefore clarified in the main text and the Table 5 legend that PO4 comprised two related behaviours: engagement in general exercise, which was intended for all participants, and secondary-prevention neck exercise, which was an optional shared-decision-making behaviour. We have also retained the explanation that enactment analysis for PO4 focused on general exercise only because interviews occurred at discharge.	**Original (Methods – EPIC-Neck programme):**<br>PO4 comprises two exercise behaviours. All patients are encouraged to engage in general exercise, however secondary-prevention neck exercise is conceptualised as an optional, shared-decision-making behaviour because evidence for recurrence prevention remains uncertain (see intervention development paper for details).33<br><br>**Updated:**<br>PO4 comprised two related behaviours: engagement in general exercise, which was intended for all participants, and secondary-prevention neck exercise, which was conceptualised as an optional shared-decision-making behaviour because evidence for recurrence prevention remains uncertain (see intervention development paper for details).33<br><br>**Original (Methods – Data analysis – qualitative):**<br>As secondary-prevention exercise was an optional shared-decision-making component and cannot be enacted at discharge, qualitative

	Section	Feedback	Response	Updated Text
				enactment analysis for PO4 focused only on general exercise.<br><br>**Updated:**<br>As secondary-prevention neck exercise was an optional shared-decision-making component and could not reasonably be enacted at discharge, qualitative enactment analysis for PO4 focused on general exercise only.<br><br>**Original (Table 5):**<br>Legend: *Secondary-prevention exercise was conceptualised as an optional, shared-decision-making behaviour. As interviews occurred at discharge, enactment of secondary-prevention exercise was not expected and was not assessed; only understanding of the benefits and risks of secondary-prevention exercise was evaluated within qualitative receipt.<br><br>**Updated:**<br>Legend: *PO4 comprised two related behaviours: engagement in general exercise, which was intended for all participants, and secondary-prevention neck exercise, which was optional and based on shared decision-making. As interviews occurred at discharge, enactment analysis for PO4 focused on general exercise only; only understanding of the benefits and risks of secondary-prevention exercise was assessed within qualitative receipt.
1.31	Tables/ Figures	Sixth, Figure S1 and S2 (the frameworks) are visually appealing but the font size is too small. When printed, the text in these figures will be illegible. Please	Thank you for this suggestion. These figures have now been provided as separate high-resolution PDF supplementary files, and the numbering/referencing of	See supplementary files 1 and 2.

	Section	Feedback	Response	Updated Text
		increase the font size or provide these as separate high-resolution files.	supplementary files has been updated throughout the manuscript.	
1.32	Tables/ Figures	Finally, several supplementary tables (especially Table S4) contain excessive text in the "Interview derived barriers" and "Interview derived facilitators" columns. Consider moving this detailed qualitative data into the main results narrative and summarising the table more concisely, or splitting Table S4 into two separate tables.	Thank you for this comment. We agree that Supplementary Table S4 contains detailed qualitative information. This level of detail was intentionally placed in the supplementary material rather than the main results to preserve readability of the manuscript while maintaining transparency around the component-level barriers and facilitators underpinning the delivery fidelity findings. We have therefore retained Table S4 in the supplementary material and did not move this detail into the main results.	No changes were made to the manuscript in response to this comment.
1.33	Discussion	I have several concerns with the discussion section. First, you open by stating that "prescribed exercise programmes were generally well received and enacted by patients, albeit selectively." However, your delivery fidelity was only 2.4/5, which is below acceptable. You seem to be downplaying this major finding. The discussion should more prominently acknowledge that the intervention was not delivered as intended, and this fundamentally limits what you can conclude about its potential effectiveness. Please restructure the opening paragraph to give equal weight to the delivery failure.	Thank you for this comment. We have revised the opening paragraph to provide more balance.	**Original (Discussion):**<br>Fidelity varied across domains and intervention components. Prescribed exercise programmes were generally well received and enacted by patients, albeit selectively. However, receipt and enactment for supporting exercise behaviours (flare-up management and general exercise) was weaker, a pattern that was consistent with the observed poorer delivery of these components.<br><br>**Updated:**<br>Fidelity varied across domains and intervention components. Although some prescribed exercise components were delivered successfully, overall delivery fidelity was below the predefined acceptable threshold, indicating that EPIC-Neck was not consistently delivered as intended. Prescribed exercises were generally well received and enacted by patients, albeit selectively. However, receipt and enactment for supporting

	Section	Feedback	Response	Updated Text
				exercise behaviours (flare-up management and general exercise) were weaker, a pattern that was consistent with poorer delivery of these components. Taken together, these findings suggest that EPIC-Neck requires optimisation of intervention content, training and implementation support before it can be considered ready for a definitive trial or wider delivery within routine NHS physiotherapy services.
1.34	Discussion	Finally, the integration sections are well done but some claims are not fully supported. For example, you state "poorer delivery appeared to contribute to weaker receipt and enactment" but you cannot infer causation from this sequential association. Please rephrase to "was associated with" rather than "contributed to".<br><br>Second, you claim that "poorer delivery appears to contribute to weaker receipt and enactment" and that this demonstrates "sequential influence" across domains. This is an overstatement. Your study is observational and correlational. You have not experimentally manipulated delivery quality to test its effect on receipt or enactment. The phrase "appears to contribute" is acceptable, but later you say "this highlights the importance of improving delivery quality, given its likely influence" – this is speculative. Please tone	Thank you for this comment. We agree that causal language should be avoided because the study was not designed to experimentally test relationships between delivery, receipt and enactment. We have revised the Discussion to use more cautious phrasing, replacing wording such as “contribute to” and “likely influence” with language indicating that poorer delivery was “consistent with” weaker receipt and enactment. We have also clarified that causal relationships between fidelity domains cannot be inferred from this study.	**Original (Discussion - Lower fidelity for supporting behaviours (PO3-4):**<br>These findings suggest a cross-domain pattern in which poorer delivery appears to contribute to weaker receipt and enactment.<br><br>This highlights the importance of improving delivery quality, given its likely influence on participant receipt and enactment.<br><br>**Updated:**<br>These findings suggest a cross-domain pattern in which poorer delivery may help explain weaker receipt and enactment, although causal relationships between fidelity domains cannot be inferred.<br><br>This supports the importance of improving delivery quality and evaluating whether delivery improvements are accompanied by stronger receipt and enactment in future work.

	Section	Feedback	Response	Updated Text
		down causal language throughout.		
1.35	Discussion	Third, you compare your findings to other studies reporting "only moderate exercise adherence" and suggest that EPIC-Neck may support better engagement for PO1. However, you did not measure adherence quantitatively (e.g., exercise diary completion rates, percentage of prescribed exercises performed). Your enactment data are qualitative only and based on participant self-report at discharge. This comparison is not valid. Either remove this claim or add a strong caveat acknowledging the lack of quantitative adherence data.	Thank you for this comment. We have removed this comparison and revised the wording to clarify that PO1 enactment reflects qualitative evidence of perceived engagement with prescribed exercises, rather than objectively or quantitatively measured adherence. We now present this as preliminary support for the underlying programme theory and acknowledge that future evaluations should include quantitative adherence measures.	**Original (Results – Enactment):**<br>Most participants reported completing their prescribed exercise programme (PO1). This contrasts with other studies reporting only moderate exercise adherence 10 20-23 and suggests that a theory-informed behaviour change approach may support patient engagement with prescribed exercise. This pattern of enactment was broadly aligned with the comparatively stronger receipt of PO1 and the relatively higher delivery scores for exercise-related and self-efficacy components.<br><br>**Updated:**<br>Most participants reported completing their prescribed exercise programme (PO1). This pattern of enactment was broadly aligned with the comparatively stronger receipt of PO1 and the relatively higher delivery scores for exercise-related and self-efficacy components. Although enactment was assessed qualitatively and not through quantitative adherence measures, these findings provide preliminary support for the underlying programme theory that behaviour change support may facilitate engagement with prescribed exercise. Future evaluations should include quantitative adherence measures, to test this more robustly.65
1.36	Discussion	Fourth, you state that "no participants started or resumed general exercise (PO4)" and attribute this to poor delivery of PO4 components. This ignores	Thank you for this comment. We agree that timing of assessment should be considered more explicitly when interpreting PO4 enactment. We have revised the Discussion to acknowledge that,	**Original (Discussion - Lower fidelity for supporting behaviours (PO3-4):**<br>No participants started or resumed general exercise (PO4), and few experiencing flare-ups

	Section	Feedback	Response	Updated Text
		an alternative explanation: the follow-up period may have been too short. Discharge occurred after a mean of 16.9 weeks. Behaviour change for general exercise often takes longer. You acknowledge this partially in the limitations but then ignore it in your main interpretation. Please discuss this alternative explanation more explicitly.	although interviews occurred after a mean intervention period of 16.9 weeks, PO4 may reasonably be introduced later in treatment, reducing the time available to observe initial enactment before interview. We have also clarified that longer-term follow-up is needed to assess maintenance of general exercise behaviours over time.	used appropriate exercise-based strategies (PO3). This contradicts the programme theory and may undermine the development of longer-term outcomes.34 68 69 Reduced enactment of these supporting exercise behaviours may partly reflect the inconsistent and/or incomplete understanding identified in the evaluation of receipt, and the relatively poor delivery of intervention components designed to support PO3 and PO4 (<2).<br><br>**Updated**<br>No participants started or resumed general exercise (PO4), and few experiencing flare-ups used appropriate exercise-based strategies (PO3). This contradicts the programme theory and may undermine the development of longer-term outcomes.34 68 69 Reduced enactment of these supporting exercise behaviours may partly reflect the inconsistent and/or incomplete understanding identified in the evaluation of receipt, and the relatively poor delivery of intervention components designed to support PO3 and PO4 (<2). However, timing of assessment should also be considered. Interviews were conducted at discharge, after a mean intervention period of 16.9 weeks; however, PO4 may reasonably be introduced later in treatment, reducing the time available for participants to start or resume general exercise before interview. Although this timeframe may have allowed initial attempts to be identified for some participants, it was unlikely to capture whether general exercise behaviours were

	Section	Feedback	Response	Updated Text
				sustained over time.70 Longer-term follow-up is therefore needed to determine whether participants initiate and sustain PO4 enactment over time.
1.37	Discussion	Fifth, you argue that training should move from a "single-day, high-volume model" to a "staged training model" with practice and feedback. This is a reasonable suggestion, but you provide no evidence from your data about what an alternative training model would look like or how many days would be sufficient. Please be more specific or present this as a hypothesis rather than a firm recommendation.	Thank you for these comments. We agree that the Discussion should be more cautious and more explicit about the uncertainty around training redesign and programme simplification. We have revised the Results section to make clearer that physiotherapists themselves suggested spreading training across sessions and including more opportunities for practice and feedback. We have revised the Discussion to present staged training, repeated practice and feedback as candidate optimisation strategies rather than firm recommendations about the required training format, duration or intensity. We have also added a more critical discussion of the need to review behavioural change components and simplify the programme where appropriate, while preserving the theoretical integrity of EPIC-Neck.	**Original:**<br>Results<br>The volume of content within a single-day format, limited practice time, and the long gap between training and intervention delivery were identified as barriers to capability development, with physiotherapists suggesting that training should be spread across sessions and include more opportunities for practice and feedback (Theme 4-5).<br><br>Discussion<br>These findings suggest that the single-day, high-volume training model may have been insufficient for skill consolidation and behavioural adoption. This is consistent with evidence that staged training models, providing opportunities to practice and refine skills with feedback, allow new components to be integrated into consultations sequentially, achieving adequate capability to deliver complex interventions 79 80 82.<br><br>However, delivery problems cannot be attributed to training alone as patient preferences, intervention complexity, perceived clinical value, resource use, and organisational pressures also influenced delivery. EPIC-Neck optimisation may also require simplifying lower-priority or lower-value components and clarifying which elements can be adapted without undermining the intervention theory.

	Section	Feedback	Response	Updated Text
				**Updated:**<br>Results<br>The volume of content within a single-day format, limited practice time, and the long gap between training and intervention delivery were identified as barriers to capability development, with physiotherapists suggesting that training should be spread across sessions and include more opportunities for practice and feedback (Theme 4-5).<br><br>Discussion<br>These findings suggest that the single-day, high-volume training model may have been insufficient for skill consolidation and behavioural adoption. This is consistent with evidence that staged training models, and feedback from physiotherapists during interviews, that training involving repeated practice, feedback and staged skill development may better support clinician behaviour change and delivery of complex interventions.79 80 82 However, the optimal format, duration and intensity of training required for EPIC-Neck warrant further investigation and should be designed with key stakeholders to ensure fit with the NHS context.<br><br>It is important to acknowledge that optimisation of training alone may not be sufficient to enhance delivery to an acceptable standard. Findings also indicated that factors relating to EPIC-Neck design and complexity influenced delivery. Optimisation should therefore examine whether the behavioural change components and overall

	Section	Feedback	Response	Updated Text
				programme structure can be simplified without undermining the intervention theory.[83] Components with lower delivery, receipt or enactment fidelity, particularly social support, problem-solving and behaviours linked to PO2–PO4, may require redesign, clearer operationalisation or repositioning within the programme. Future refinement should clarify which components are essential versus adaptable, reduce avoidable cognitive and practical burden, and specify how behavioural change components can be tailored while preserving the intended mechanisms of action.[83-85]
1.38	Discussion	Sixth, you list several limitations but you missed at least three important ones: (1) the fidelity checklist was designed by the intervention developer (JP), introducing potential bias; (2) you did not assess inter-rater reliability beyond 75% agreement within 1 point, which is a weak threshold; and (3) the physiotherapists knew they were being audio-recorded, which may have altered their behaviour (Hawthorne effect). Please add these to the limitations paragraph.	Thank you for this comment. We agree that these are important limitations. We have expanded the limitations section to acknowledge that the delivery-fidelity checklist was developed by the lead intervention developer (JP), although it was also independently reviewed by CG, a health psychologist with expertise in behaviour change interventions. We have additionally acknowledged that no formal inter-rater reliability statistic was calculated following the calibration process, and that physiotherapists knew appointments were being audio-recorded, which may have influenced behaviour and led to an overestimation of delivery fidelity.	**Original (Limitations):**<br>Limitations include enactment being assessed at discharge, which may not have fully captured longer-term behaviours and the use of non-validated questionnaires; however, these were purpose-designed to align with the EPIC-Neck programme theory and fidelity framework. In addition, the breadth of interview topics may have limited the depth of exploration across fidelity domains.<br><br>**Updated:**<br>Several limitations should be considered. Beyond the initial Intervention Mapping process, no further independent assessment of design fidelity was undertaken. Enactment was assessed qualitatively at discharge, with no quantitative or objective measures, limiting assessment of longer-term behaviour maintenance. However, interviews provided insight into enactment, adaptation, barriers and facilitators. Future

	Section	Feedback	Response	Updated Text
				evaluations should consider combining qualitative methods with quantitative measures of adherence or enactment, such as ATEMPT.66<br>The receipt questionnaires and delivery-fidelity checklist were purpose-designed for EPIC-Neck and NIH-BCC fidelity domains but were not externally validated. Although the delivery-fidelity checklist was reviewed by CG, final scoring was undertaken by JP, introducing potential bias. Calibration was conducted with a second researcher before single coding, but no formal inter-rater reliability statistic was calculated.<br>Physiotherapists were aware that appointments were being audio-recorded, which may have altered behaviour and overestimated delivery fidelity, although recording enabled direct assessment rather than reliance on self-report.36 Finally, interviews were conducted and analysed by JP, who had existing professional relationships with some physiotherapists, and no formal independent double-coding was undertaken. Independent interviewing and coding would have strengthened the evaluation, although credibility was supported through structured topic guides, confidentiality procedures, team review of coding decisions, and an audit trail.
1.39	Conclusion	Finally, the conclusion states that "optimisation of intervention content, training, and implementation processes, followed by repeat fidelity evaluation, appears warranted" before a definitive trial. I agree, but	Thank you for these comments. We agree that the conclusion should more clearly identify the main optimisation priorities, while avoiding overly prescriptive recommendations that this study was not designed to determine. We have revised the conclusion to highlight the	**Original (Conclusion):**<br>This multidimensional fidelity evaluation showed that fidelity of the EPIC-Neck intervention varied across domains and components. Receipt and enactment were relatively stronger for performing prescribed neck exercises than

	Section	Feedback	Response	Updated Text
		this is obvious given your findings. A stronger conclusion would specify the top three specific changes you recommend (e.g., (1) extend training to two days with supervised practice, (2) simplify PO4 delivery, (3) add individualised feedback on audio-recorded sessions). Please be more specific.	need to review physiotherapist training, strengthen practice and feedback processes, consider simplification of the intervention design while preserving intended mechanisms, clarify essential versus adaptable components, and repeat fidelity evaluation before progression to a definitive trial.	for longer-term condition-management behaviours (PO2-4), although enactment was selective and declined for more challenging exercises. These patterns appeared to be linked, at least in part, to suboptimal delivery of several components that required physiotherapists to develop new skills and change existing practice. Optimisation of intervention content, training, and implementation processes, followed by repeat fidelity evaluation, appears warranted before progressing to a definitive trial.<br><br>**Updated:**<br>This multidimensional fidelity evaluation showed that fidelity of the EPIC-Neck intervention varied across domains and components. Receipt and enactment were relatively stronger for performing prescribed neck exercises than for longer-term condition-management behaviours (PO2-4), although enactment was selective and declined for more challenging exercises. These patterns appeared to be linked, at least in part, to suboptimal delivery of several components that required physiotherapists to develop new skills and change existing practice.<br><br>EPIC-Neck requires optimisation before progression to a definitive trial. Refinements should focus on strengthening physiotherapist training and support, including candidate strategies such as staged learning, additional practice and regular feedback, to help clinicians develop and maintain the skills required for delivery. Optimisation should

	Section	Feedback	Response	Updated Text
				also consider simplifying the intervention design and clarifying essential versus adaptable components, while preserving the intended mechanisms of action. Repeat fidelity evaluation is needed before progressing to a definitive trial evaluating long-term effectiveness and engagement.

Reviewer 2

	Section	Feedback	Response	Updated Text
2.1	General	The authors present a good research paper.<br><br>• Relevance of the topic: Excellent.<br>• Introduction: Good.<br>• Methodology: Regular.<br>• Results: Good.<br>• Discussion: Good.<br>• Conclusions: Bad<br><br>However, Reconsider after major revision. In general, the paper follows an adequate structure and correct scientific support and can be published considering some limitations. However, there are a series of limitations that should be considered.	Thank you for a positive response. We are sure that the feedback from the peer-review process will strengthen the manuscript	-
2.2	General	The author must rewrite the whole text in an impersonal manner and try not to use the pronoun "we".	Thank you for this comment. The manuscript was reviewed carefully for use of first-person language and author-centred phrasing. No use of first-person pronouns (e.g., "we", "our", "us") was identified within the main text. The manuscript is written in standard scientific prose and primarily uses an impersonal style. Consequently, no major revisions were required in response to this comment.	No changes were made to the manuscript in response to this comment.

	Section	Feedback	Response	Updated Text
2.3	General	In the same way, reviewing each section's verb tense is recommended. Results (past tense), Introduction (past tense), Method (past tense), Results (past tense), Discussion (present tense), and Conclusions (present tense).	Thank you for this suggestion. We reviewed verb tense throughout the manuscript. While there are no universally accepted rules prescribing a single tense for each manuscript section, Methods and Results are conventionally reported in the past tense, which is already the predominant style used here. After reviewing the manuscript, we did not identify any substantive tense inconsistencies and are confident that the current tense usage is appropriate.	No changes were made to the manuscript in response to this comment.
2.4	Title/ Abstract	Title and abstract<br>The title is clear and appropriate.	Thank you for the feedback. In response to other reviewers feedback the title has been shortened and the country has been added (See comments 0.1, 1.1 above).	**Original:**<br>Multidimensional fidelity of a co-developed, theory and evidence-based exercise programme for chronic non-specific neck pain: a process evaluation of the EPIC-Neck programme<br><br>**Updated:**<br>Multidimensional fidelity of the EPIC-Neck exercise programme for chronic non-specific neck pain: a UK feasibility-trial process evaluation
2.5	Title/ Abstract	The abstract is comprehensive, but could place greater emphasis on low adherence to the intervention and its implications.	Thank you for this comment. We agree that engagement with the intervention is an important finding. Physiotherapist adherence to the intervention (delivery fidelity) is already highlighted in the abstract through reporting that overall delivery fidelity was below the acceptable threshold and that EPIC-Neck was not consistently delivered as intended. Participant adherence/enactment is also addressed through reporting that participants generally understood and enacted prescribed neck exercises, but that receipt and enactment were weaker for longer-term self-management behaviours (PO2-PO4). Given the editor's request to reduce the length of the abstract, we did not make further changes in response to this comment.	No changes were made to the manuscript in response to this comment.

	Section	Feedback	Response	Updated Text
2.6	Background	Well-structured and the use of references is correct, and they are updated. However, this section could be improved:<br>Include more recent evidence on adherence to exercise in musculoskeletal pain.	Thank you for this suggestion. We reviewed the adherence-related wording and citations in the Introduction. As this manuscript evaluates multidimensional fidelity rather than patient exercise adherence alone, we have retained a concise rationale focused primarily on chronic non-specific neck pain. We also note that "adherence" can refer to different constructs in this context, including participant enactment of exercise behaviours and physiotherapist delivery of intervention components as intended; both are addressed within the NIH-BCC fidelity framework used in this study. To acknowledge the broader musculoskeletal literature, we have added a recent citation on exercise adherence and outcomes in non-specific low back pain, while avoiding expansion of this section beyond the focus of the paper.	Added citation<br><br>Jones MD, Hansford HJ, Bastianon A, et al. Exercise adherence is associated with improvements in pain intensity and functional limitations in adults with chronic non-specific low back pain: a secondary analysis of a Cochrane review. J Physiother 2025;71(2):91-99. doi: 10.1016/j.jphys.2025.03.004 [published Online First: 20250401]
2.7	Background	Better explain why the NIH-BCC was chosen over other frameworks.	Thank you for this comment. We have added a clearer justification for selecting the NIH-BCC framework.	**Original (Background):** Despite this, fidelity remains under-evaluated and inconsistently reported within musculoskeletal research.23 37-39 The National Institute of Health Behaviour Change Consortium (NIH-BCC) provides a multidimensional framework, comprising five sequential domains across design, training, delivery, receipt, and enactment allowing a complete assessment of fidelity.40 Therefore, this process evaluation aimed to establish the multidimensional fidelity of the EPIC-Neck programme and identify refinements before progressing to a full-scale effectiveness trial.

	Section	Feedback	Response	Updated Text
				**Updated:**<br>Despite this, fidelity remains under-evaluated and inconsistently reported within musculoskeletal research.1-4 This evaluation was guided by the National Institutes of Health Behaviour Change Consortium (NIH-BCC) framework because it is a well-established and highly cited framework for behavioural interventions, has been used in multidimensional fidelity evaluations, and provides coverage of domains relevant to complex intervention evaluation.5 6 7 The NIH-BCC framework comprises five domains: design, training, delivery, receipt and enactment, aligning with the EPIC-Neck programme theory by assessing whether physiotherapists were trained to deliver the intervention, whether EPIC-Neck was delivered as intended, and whether participants understood and enacted the intended exercise behaviours.5 Therefore, this process evaluation aimed to establish the multidimensional fidelity of the EPIC-Neck programme and identify refinements before progressing to a full-scale effectiveness trial.

	Section	Feedback	Response	Updated Text
2.8	Methods	The methods and procedures followed are well defined and explain the main information about the study in order to be replicated. In this line, some issues can be explained:<br>Why did you select a threshold of ≥3/5 was chosen as 'acceptable'	Thank you for this comment. We have clarified the rationale for using a threshold of ≥3/5. Delivery quality was assessed using the Dreyfus clinical competence scale, where a score of 3 represents acceptable competence and a score of 5 represents expert/perfect delivery. Consequently, a score of ≥3/5 was used to indicate acceptable delivery fidelity. This approach is consistent with previous intervention fidelity studies that have used Dreyfus-based competency scoring frameworks.	**Original (Methods – data collection – quantitative – delivery):**<br>Delivery quality of checklist items were scored using the Dreyfus clinical competence scale (0–5), where 3 indicated acceptable and 5 perfect delivery.59<br><br>**Updated:**<br>Delivery quality of checklist items was scored using the Dreyfus clinical competence scale (0-5), where scores represent increasing levels of competence, with 3 indicating acceptable delivery and 5 indicating expert/perfect delivery.59 Consistent with previous fidelity research using this approach, a score of ≥3/5 was used as the threshold for acceptable delivery fidelity.51 52 60
2.9	Methods	Justify the decision not to assess design fidelity beyond the reference to the previous article.	Thank you for these comments. We have revised the Methods section. See response 1.13 above for more details.	See response 1.13 above.
2.10	Results	Clearly presented, but:<br>The table showing fidelity by component should include confidence intervals.	Thank you for this suggestion. The table has been updated to include n and 95%CI values.	See table 4
2.11	Results	It would be useful to add a flowchart of participants in the fidelity assessment.	Thank you for this suggestion. We have added Figure 1: a flowchart of participants in the fidelity assessment	Figure 1. Participant contribution to fidelity evaluation datasets.

2.12	Discussion	This section is well structured and discusses the main results of the study according to previous research. However, some sentences and affirmations are too descriptive. A critical analysis is lacking regarding:<br>The actual feasibility of the programme within the NHS.	Thank you for this comment. We agree that the Discussion should more explicitly consider the implications of the fidelity findings for NHS deliverability. As the broader feasibility outcomes are reported in the companion feasibility manuscript, we have focused here on what the fidelity findings indicate about deliverability of EPIC-Neck within routine NHS physiotherapy services. We have revised the opening Discussion paragraph to state that the below-threshold delivery fidelity raises questions about current NHS deliverability and indicates that optimisation of intervention content, training and implementation support is needed before definitive trial evaluation.	**Original (Discussion):**<br>Fidelity varied across domains and intervention components. Prescribed exercise programmes were generally well received and enacted by patients, albeit selectively. However, receipt and enactment for supporting exercise behaviours (flare-up management and general exercise) was weaker, a pattern that was consistent with the observed poorer delivery of these components.<br><br>**Updated:**<br>Fidelity varied across domains and intervention components. Although some prescribed exercise components were delivered successfully, overall delivery fidelity was below the predefined acceptable threshold, indicating that EPIC-Neck was not consistently delivered as intended. Prescribed exercises were generally well received and enacted by patients, albeit selectively. However, receipt and enactment for supporting exercise behaviours (flare-up management and general exercise) were weaker, a pattern that was consistent with poorer delivery of these components. Taken together, these findings suggest that EPIC-Neck requires optimisation of intervention content, training and implementation support before it can be considered ready for a definitive trial or wider

	Section	Feedback	Response	Updated Text
				delivery within routine NHS physiotherapy services.
2.13	Discussion	A critical analysis is lacking regarding:<br>The possible simplification of the programme.<br>The need to redesign behavioural change components.	Thank you for these comments. We believe these comments have now been resolved under response 1.37 above.	See response 1.37 above
2.14	Conclusion	It is necessary to include a review of the training.<br>To summarise the programme before a definitive trial.	Thank you for these comments. We agree that the conclusion should more clearly identify the main optimisation priorities, while avoiding overly prescriptive recommendations that this study was not designed to determine. We have revised the conclusion to highlight the need to review physiotherapist training, strengthen practice and feedback processes, consider simplification of the intervention design while preserving intended mechanisms, clarify essential versus adaptable components, and repeat fidelity evaluation before progression to a definitive trial.	**Original (Conclusion):**<br>This multidimensional fidelity evaluation showed that fidelity of the EPIC-Neck intervention varied across domains and components. Receipt and enactment were relatively stronger for performing prescribed neck exercises than for longer-term condition-management behaviours (PO2-4), although enactment was selective and declined for more challenging exercises. These patterns appeared to be linked, at least in part, to suboptimal delivery of several components that required physiotherapists to develop new skills and change existing practice. Optimisation of intervention content, training, and implementation processes, followed by repeat fidelity evaluation, appears warranted before progressing to a definitive trial.<br><br>**Updated:**<br>This multidimensional fidelity evaluation showed that fidelity of the EPIC-Neck intervention varied

	Section	Feedback	Response	Updated Text
				across domains and components. Receipt and enactment were relatively stronger for performing prescribed neck exercises than for longer-term condition-management behaviours (PO2-4), although enactment was selective and declined for more challenging exercises. These patterns appeared to be linked, at least in part, to suboptimal delivery of several components that required physiotherapists to develop new skills and change existing practice.<br><br>EPIC-Neck requires optimisation before progression to a definitive trial. Refinements should focus on strengthening physiotherapist training and support, including candidate strategies such as staged learning, additional practice and regular feedback, to help clinicians develop and maintain the skills required for delivery. Optimisation should also consider simplifying the intervention design and clarifying essential versus adaptable components, while preserving the intended mechanisms of action. Repeat fidelity evaluation is needed before progressing to a definitive trial evaluating long-term effectiveness and engagement.

Reviewer 3

	Section	Feedback	Response	Updated Text
3.1	Title/ Abstract	The title of the manuscript appears overly long and somewhat cluttered, which makes it difficult to immediately grasp the main focus of the study. A more concise and clearer title would improve readability and help readers quickly understand the key message of the paper.	Thank you for the feedback. The title has been shortened and the country has been added.	**Original:**<br>Multidimensional fidelity of a co-developed, theory and evidence-based exercise programme for chronic non-specific neck pain: a process evaluation of the EPIC-Neck programme<br><br>**Updated:**<br>Multidimensional fidelity of the EPIC-Neck exercise programme for chronic non-specific neck pain: a UK feasibility-trial process evaluation
3.2	Title/ Abstract	The abbreviation "EPIC Neck" is introduced without first providing its full term. Please ensure that the full name of the intervention is written out at its first appearance in the manuscript, followed by the abbreviation in parentheses.	Thank you. The manuscript has been updated.	**Original (Abstract):**<br>Objectives: To evaluate multidimensional fidelity of the EPIC-Neck programme.<br><br>**Updated:**<br>Objectives: To evaluate multidimensional fidelity of the EPIC-Neck programme (Exercise Prescription Improved through Co-design).
3.3	Background	The EPIC Neck program is briefly introduced in the Introduction; however, the description remains quite general.	Thank you for this comment. We have added a brief description of EPIC-Neck in the Introduction, while retaining the detailed intervention description in the Methods section.	**Original (Background):**<br>Using these principles, the EPIC-Neck programme (Exercise Prescription Improved through Co-design), a co-developed, theory and evidence informed exercise programme, was developed using Intervention Mapping.32 33 The programme was designed to maximise engagement with and the effectiveness of neck exercise for people with chronic non-specific neck pain.<br><br>**Updated:**<br>Using these principles, the EPIC-Neck programme (Exercise Prescription Improved through Co-design), a co-developed, theory and evidence informed exercise programme, was developed using

	Section	Feedback	Response	Updated Text
				Intervention Mapping.32 33 The programme was designed to maximise engagement with and the effectiveness of neck exercise for people with chronic non-specific neck pain. EPIC-Neck combines individualised, physiotherapist-prescribed biopsychosocial exercise with behaviour change strategies and patient-facing resources to support exercise-based self-management.
3.4	Methods	The study appears to include two groups; however, this section refers only to participants randomized to the EPIC Neck intervention. The authors should clarify whether the fidelity evaluation was conducted exclusively for the intervention group and explain how participants in the control group were considered in the study. Providing a clearer explanation would help readers better understand the analytical approach.	Thank you for these comments. We have revised the Study design section to state the aim of the parent feasibility trial before describing the nested multidimensional fidelity evaluation. We have clarified that the multidimensional fidelity evaluation focused on the EPIC-Neck intervention. Usual exercise care appointments were included only to assess concordance with EPIC-Neck components and explore intervention distinctness/contamination, rather than to evaluate fidelity of usual care to its own intended model.	**Original (Methods):**<br>Study design<br>A mixed methods fidelity evaluation was embedded in the EPIC-Neck randomised controlled feasibility study, where patients attending NHS musculoskeletal outpatient physiotherapy services in the UK were randomised to receive the EPIC-Neck programme or usual exercise care (physiotherapists' routine practice).44<br>Multidimensional fidelity evaluation<br>EPIC-Neck fidelity evaluation was guided by the NIH-BCC framework covering training, delivery, receipt and enactment, using a mixture of qualitative and quantitative methods (Table 1)36 41 56 57.<br><br>**Updated:**<br>Study design<br>This mixed-methods process evaluation was embedded within the EPIC-Neck randomised controlled feasibility study, which aimed to determine the feasibility of a definitive trial evaluating the clinical and cost-effectiveness of EPIC-Neck compared with usual exercise care for adults with chronic non-specific neck pain. In the parent feasibility study, patients attending UK NHS musculoskeletal outpatient physiotherapy services were randomised to EPIC-Neck or usual exercise care

	Section	Feedback	Response	Updated Text
				(physiotherapists' routine practice).41 The present study reports the nested multidimensional fidelity evaluation of the EPIC-Neck intervention. Usual exercise care appointments were included only to examine whether EPIC-Neck components appeared in usual care.<br>Multidimensional fidelity<br>Fidelity evaluation of the EPIC-Neck programme was guided by the NIH-BCC framework covering training, delivery, receipt and enactment, using a mixture of qualitative and quantitative methods (Table 1).36 41 56 57
3.5	Methods	Include references for line 147 to 149	Thank you. We have added the reference again at this point in the text to improve clarity for readers.	**Original (Methods – EPIC-Neck programme):**<br>Full details of the EPIC-Neck (Exercise Prescription Improved through Co-design) programme, development processes and its theoretical underpinnings are published elsewhere.33 In brief, EPIC-Neck is an individualised, biopsychosocial, evidence-based, theory-informed intervention co-designed with patients and experts to support self-management of chronic neck pain through a home-based exercise programme. It integrates tailored exercise prescription with structured behaviour change support.<br><br>**Updated:**<br>Full details of the EPIC-Neck (Exercise Prescription Improved through Co-design) programme, development processes and its theoretical underpinnings are published elsewhere.33 In brief, EPIC-Neck is an individualised, biopsychosocial, evidence-based, theory-informed intervention co-designed with patients and experts to support self-management of chronic neck pain through a home-based exercise programme.33 It integrates tailored exercise prescription with structured behaviour change support.33

	Section	Feedback	Response	Updated Text
3.6	Methods	The manuscript refers to participants with "non specific neck pain"; however, the definition of this condition is not clearly described. The authors should clarify the diagnostic criteria used and who was responsible for making the diagnosis	Thank you. We have clarified the eligibility criteria by explicitly defining chronic non-specific neck pain and key exclusions, while retaining reference to the published protocol for full details.	**Original (Methods – Patient participants):**<br>Adults (≥18 years) with chronic non-specific neck pain were recruited from NHS community-based and secondary care musculoskeletal services in Berkshire, Birmingham, and Nottingham. Exclusion criteria included neck pain due to trauma, a specific diagnosis (e.g., osteoarthritis) and neurological involvement. Full eligibility criteria are reported elsewhere.41<br><br>**Updated:**<br>Adults (≥18 years) with chronic (≥3 months) non-specific neck pain were recruited from NHS community-based and secondary care musculoskeletal services in Berkshire, Birmingham, and Nottingham. Non-specific neck pain was defined as pain or discomfort in the neck and/or shoulder girdle, with or without referred arm pain, for which no specific cause could be identified.51Exclusion criteria included neck pain due to trauma (e.g., whiplash), a specific diagnosis (e.g., rheumatoid arthritis, osteoarthritis) and neurological involvement (e.g., radiculopathy, myelopathy). Full eligibility criteria are reported elsewhere.41
3.7	Methods	The manuscript states that physiotherapists completed a questionnaire before and after the training; however, the questionnaire is not clearly described. The authors should clarify whether this was a previously validated questionnaire or a study specific instrument developed for this research.	Thank you. We have clarified that the pre/post training confidence questionnaire and post-training survey were study-specific instruments developed to align with the EPIC-Neck logic model and fidelity objectives. Further details are in Supplementary File 3 Table S2 and Data S1.	**Original (Methods – Data collection – quantitative – training):**<br>Physiotherapists completed a questionnaire immediately pre and post training, rating confidence in delivering key components on a 5-point Likert scale (1 = not confident, 3 = somewhat confident, 5 = completely confident) and a post-training survey evaluating training satisfaction, strengths/limitations, and suggested refinements.

	Section	Feedback	Response	Updated Text
				**Updated:**<br>Physiotherapists completed a questionnaire immediately pre and post training, rating confidence in delivering key components on a 5-point Likert scale (1 = not confident, 3 = somewhat confident, 5 = completely confident) and a post-training survey evaluating training satisfaction, strengths/limitations, and suggested refinements. These questionnaires were study-specific measures developed to align with the EPIC-Neck logic model and fidelity objectives and were not formally validated.
3.8	Methods	Explain more details from delivery checklist and receipt questionnaire	Thank you for this comment. We believe the delivery checklist and receipt questionnaire were already described sufficiently for the main manuscript, with fuller detail provided in the supplementary material. To improve clarity, we have slightly rephrased the description of the receipt questionnaire to make explicit that it comprised one item for each performance objective (PO1–PO3).	**Original (Methods – data collection quantitative receipt):**<br>Confidence completing performance objectives 1-3 was assessed using a 3-item questionnaire (one question for each PO) with an anchored 1-5 Likert scale (1 = very unconfident, 3 = no opinion, 5 = very confident), posted to participants at 6 months post-randomisation.56 57<br><br>**Updated:**<br>Confidence completing performance objectives 1–3 was assessed at 6 months post-randomisation using a 3-item questionnaire, comprising one item for each performance objective, with an anchored 1–5 Likert scale (1 = very unconfident, 3 = no opinion, 5 = very confident).56 57

	Section	Feedback	Response	Updated Text
3.9	Results	The authors state that "distinct differences" were noted between the EPIC-Neck and Usual Exercise Care groups. While the descriptive data (means and SDs) support this, the authors should clarify if any statistical significance testing (e.g., t-tests or non-parametric equivalents) was performed to support the use of the term "distinct differences." If not, it might be more appropriate to describe these as "descriptive differences."	Thank you for this comment. The comparison between EPIC-Neck and usual exercise care was included descriptively to explore potential contamination between study arms and to provide contextual information regarding concordance of usual care with EPIC-Neck components. The analysis was not designed or powered as a formal between-group comparison, and no inferential statistical testing was planned. To avoid implying formal statistical comparison, the wording has been revised from 'distinct differences' to 'descriptive differences' where appropriate.	**Original (Results – Delivery – Quantitative):**<br>Distinct differences were noted between EPIC-Neck and Usual Exercise Care. Usual Exercise Care had a mean fidelity score of 1.1 (SD 1.0; range 0.6–1.5), with only one component (improving self-efficacy) delivered in a manner consistent with the EPIC-Neck protocol (Table 4). Full participant-by-component fidelity scores for both study arms are provided in Supplementary File 1 – Table S5-6.<br><br>**Updated:**<br>Usual exercise care appointments contained few EPIC-Neck components, suggesting limited contamination between study arms. When scored against the EPIC-Neck checklist, usual exercise care had a mean concordance score of 1.1 (SD 1.0; range 0.6–1.5). These descriptive differences suggest that usual exercise care rarely incorporated EPIC-Neck components. Only one component (improving self-efficacy) was delivered in a manner consistent with the EPIC-Neck protocol (Table 4). Full participant-by-component checklist scores for both study arms are provided in Supplementary File 1 – Tables S5–S6.

VERSION 2 - REVIEW

**Reviewer**	**1**
**Name**	**Bagherian, Sajad**
**Affiliation**	**Shahrekord University**
**Date**	**31-Jul-2026**
**COI**	

This is a well-conducted multidimensional fidelity evaluation that makes an important contribution to the literature on complex intervention implementation in musculoskeletal physiotherapy. The authors have responded comprehensively to the previous round of reviewer comments, making substantial improvements to the manuscript.
